# A split, conditionally active mimetic of IL-2 reduces the toxicity of systemic cytokine therapy

**DOI:** 10.1038/s41587-022-01510-z

**Published:** 2022-10-31

**Authors:** Alfredo Quijano-Rubio, Aladdin M. Bhuiyan, Huilin Yang, Isabel Leung, Elisa Bello, Lestat R. Ali, Kevin Zhangxu, Jilliane Perkins, Jung-Ho Chun, Wentao Wang, Marc J. Lajoie, Rashmi Ravichandran, Yun-Huai Kuo, Stephanie K. Dougan, Stanley R. Riddell, Jamie B. Spangler, Michael Dougan, Daniel-Adriano Silva, David Baker

**Affiliations:** 1 Department of Biochemistry and Institute for Protein Design, University of Washington, Seattle, WA, USA.; 2 Department of Bioengineering, University of Washington, Seattle, WA, USA.; 3 Department of Cancer Immunology and Virology, Dana-Farber Cancer Institute, Boston, MA, USA.; 4 Department of Medicine, Division of Gastroenterology, Massachusetts General Hospital and Harvard Medical School, Boston, MA, USA.; 5 Department of Chemical & Biomolecular Engineering, Johns Hopkins University, Baltimore, MD, USA.; 6 Fred Hutchinson Cancer Research Center, Clinical Research Division, Seattle, WA, USA.; 7 Department of Biomedical Engineering, Johns Hopkins University, Baltimore, MD, USA.; 8 Department of Immunology, Harvard Medical School, Boston, MA, USA.; 9 Howard Hughes Medical Institute, University of Washington, Seattle, WA, USA.; 10 Present address: Monod Bio, Inc., Seattle, WA, USA.; 11 Present address: Outpace Bio, Seattle, WA, USA.; 12 Present address: Division of Life Science, The Hong Kong University of Science and Technology, Hong Kong, China.; 13 These authors contributed equally: Aladdin M. Bhuiyan, Huilin Yang, Isabel Leung, Elisa Bello

## Abstract

The therapeutic potential of recombinant cytokines has been limited by the severe side effects of systemic administration. We describe a strategy to reduce the dose-limiting toxicities of monomeric cytokines by designing two components that require colocalization for activity and that can be independently targeted to restrict activity to cells expressing two surface markers. We demonstrate the approach with a previously designed mimetic of cytokines interleukin-2 and interleukin-15—Neoleukin-2/15 (Neo-2/15)—both for *trans*-activating immune cells surrounding targeted tumor cells and for *cis*-activating directly targeted immune cells. In *trans*-activation mode, tumor antigen targeting of the two components enhanced antitumor activity and attenuated toxicity compared with systemic treatment in syngeneic mouse melanoma models. In *cis*-activation mode, immune cell targeting of the two components selectively expanded CD8^+^ T cells in a syngeneic mouse melanoma model and promoted chimeric antigen receptor T cell activation in a lymphoma xenograft model, enhancing antitumor efficacy in both cases.

Immunostimulatory cytokines are central regulators of the immune system and have great potential as immunotherapeutic agents^1,2^. However, their clinical use has been limited by severe toxicities resulting from systemic immune activation^2,3^. Fusions of cytokines to monoclonal antibodies or antibody fragments (immunocytokines) have been developed with the aim of improving efficacy and reducing systemic toxicity by targeting cytokine activity^4,5^. However, the success of this approach has been limited by the broad distribution of cytokine receptors^6,7^. Additional strategies to activate cytokines specifically at tumor sites include reducing the affinity of the cytokine moiety to improve targeting^8–10^, cytokine fusions to inhibitory moieties that can be cleaved by tumor-associated proteases^11–13^ and versions of oligomeric cytokines that are activated by colocalization of monomeric subunits^14,15^. However, for monomeric cytokines such as interleukin (IL)-2, the design of immunotherapeutics with effective activity on demand has remained elusive.

Human IL-2 (IL-2) is a potent pleiotropic cytokine approved for treatment of melanoma and renal cell carcinoma^16^, but its therapeutic potential is limited by preferential activation of CD25^+^ cells and severe dose-limiting toxicities associated with systemic IL-2 activity^3^. As a result, IL-2 has been at the center of cytokine engineering efforts, leading to the development of multiple new drugs currently in preclinical development or clinical trials^5,17^. Like most natural proteins, IL-2 has structural irregularities that translate into nonideal biochemical properties, such as low solubility and poor mutational robustness^18,19^, which hamper efforts to repurpose it as a conditionally active therapeutic agent. Recent advances in computational protein design^20^ have enabled the generation of highly stable functional proteins that fully recapitulate the activity of natural cytokines, but with improved biochemical and therapeutic properties^21,22^. The de novo designed IL-2 mimetic Neoleukin-2/15 (Neo-2/15) reproduces the immunostimulatory function of IL-2 and IL-15 by activation of IL-2Rβ and IL-2Rγ but is fully independent of the CD25 and CD215 receptors^22^ (other approaches have also been used to generate IL-2 variants with reduced CD25 affinity^23–25^). This mimetic induces potent immunotherapeutic effects with reduced toxicity when compared with natural IL-2, by avoiding undesired preferential activation of CD25^+^ immune cells^22^. A pegylated version (NL-201) has shown improved therapeutic effect in multiple preclinical models^26^ and is currently being tested in clinical trials^27^. Nevertheless, without additional localization strategies, systemic overactivation due to on-target, off-tumor activation of immune cells could still lead to toxicity at high doses. We reasoned that the high stability and robust folding of Neo-2/15 could be leveraged to create a conditionally active cytokine to safely and selectively target IL-2 mediated immune cell activation where needed. To this end, we set out to design two-component split versions of Neo-2/15 that are active only upon colocalization of the two disjointed fragments at the site of the tumor.

## Results

### Development of functional split Neo-2/15 variants

**Extended Data Fig. 1 |:**
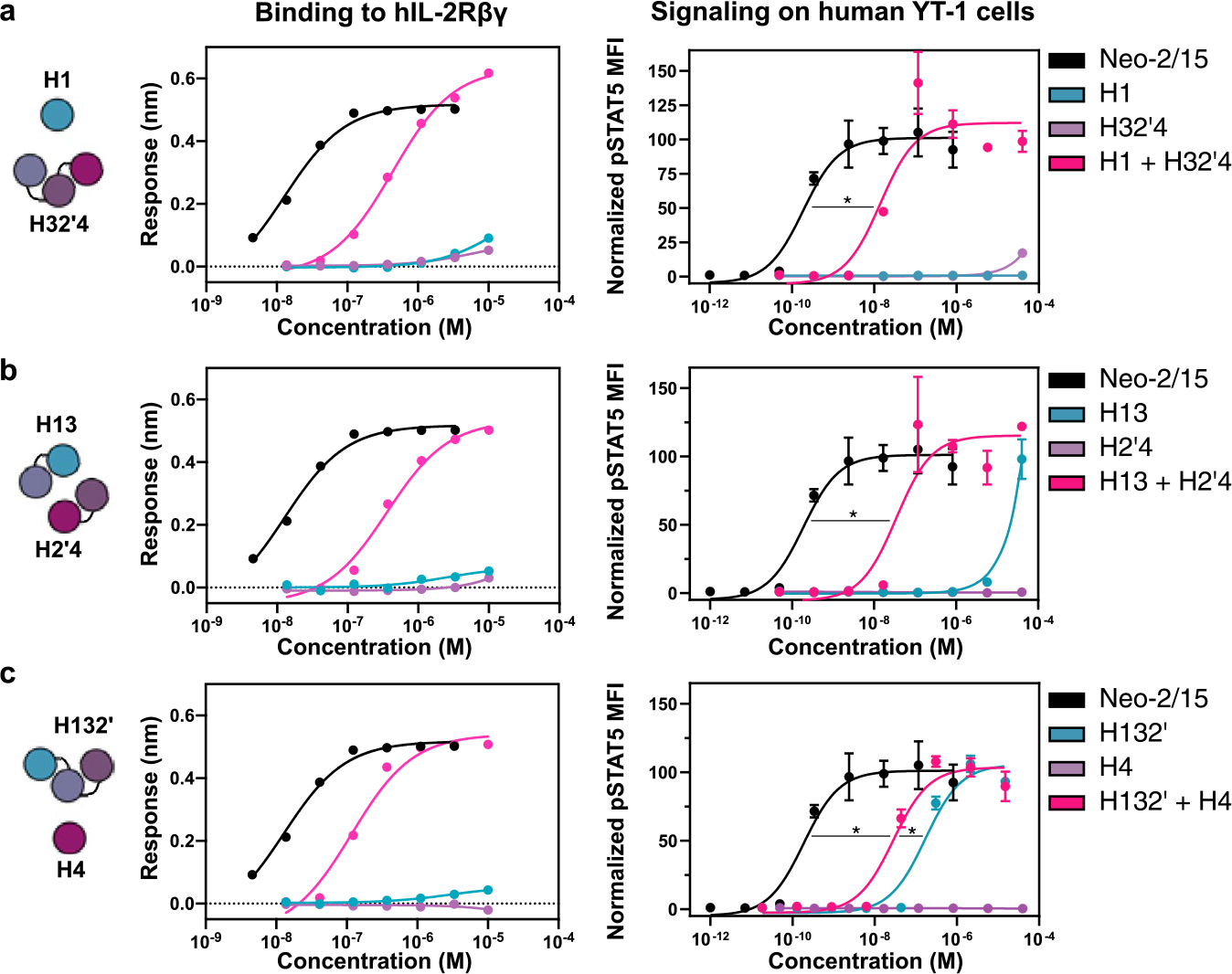
IL-2 receptor binding and immune cell signaling activity of Split Neo-2/15 pairs. Binding to immobilized IL-2Rβγ measured via biolayer interferometry (left) and STAT5 phosphorylation response (right) of YT-1 human NK cells for Neo-2/15 (black), one split fragment (blue), a second split fragment (purple) and the combination of the two split fragments (magenta). Results are shown for three split pairs: H1 + H32’4 (**a**); H13 + H2’4 (**b**); and H132’ + H4 (**c**). The H1 + H32’4 (Neo2A + Neo2B) pair demonstrates the optimal conditional activation profile. The calculated EC50 values for the STAT5 phosphorylation (**a-c**, right panels) are: Neo-2/15 0.187 nM; H1 + H32’4 14 nM; H13 + H2’4 34.6 nM; H132’ + H4 28.3 nM; H1 > 40 μM; H32’4 > 40 μM; H13 > 40 μM; H2’4 > 40 μM; H132’ 171 nM; H4 > 40 μM. * indicates different EC50 values with non-overlapping 95% confidence interval ranges. Experiments were performed in triplicate three times with similar results. Data are presented as mean values +/− s.d.

**Extended Data Fig. 2 |:**
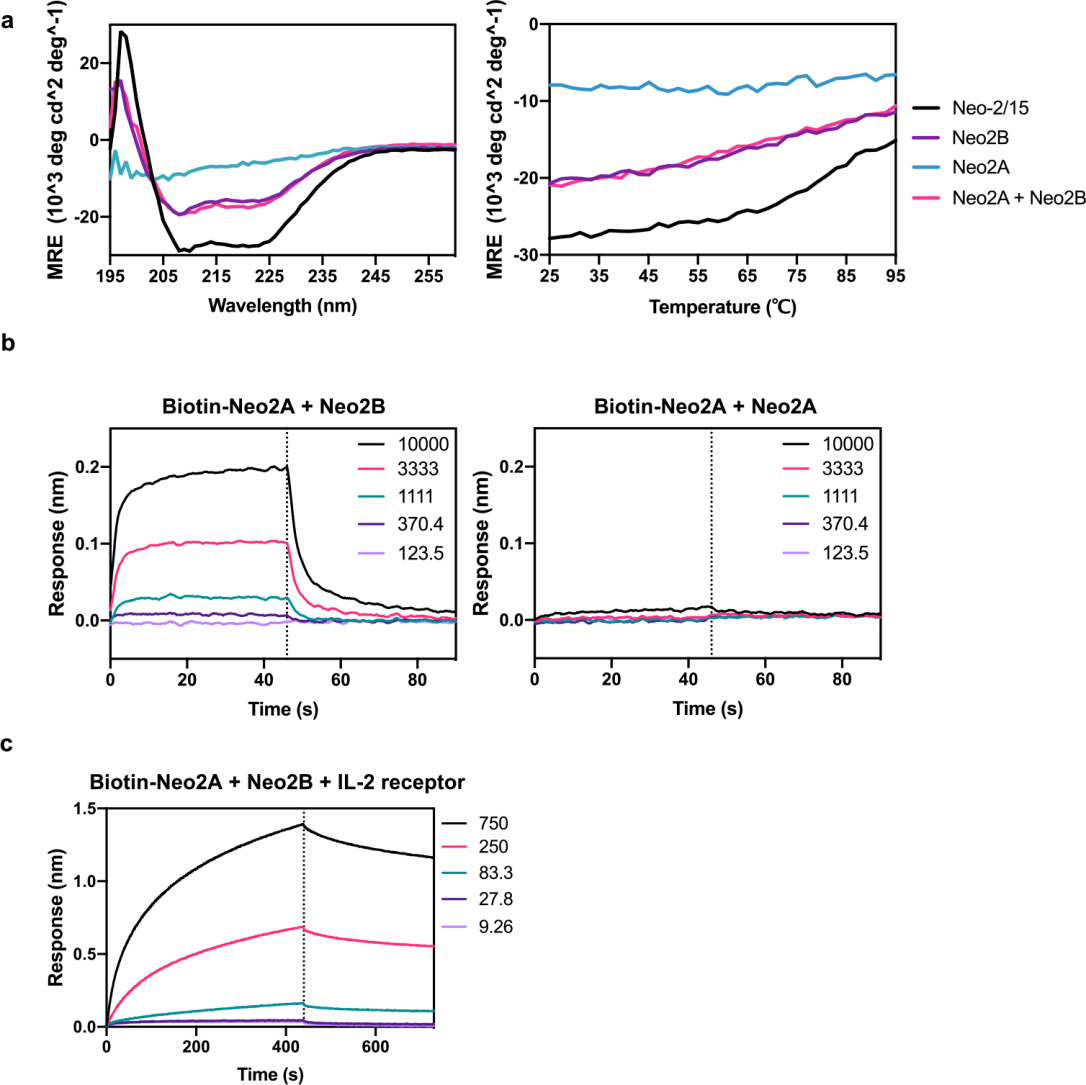
Biophysical analyses of split Neo-2/15 fragments Neo2A and Neo2B. **a**, Left: Circular dichroism spectra of Neo-2/15 (black) and the Split Neo-2/15 fragments individually (blue and purple) or in combination (magenta). Neo2B shows negative ellipticity at 210 and 222 nm, indicating an alpha helical secondary structure. Right: Thermal melts of each split fragment, monitored by their signal at 222 nm during heating from 25 °C to 95 °C (heating rate ~2 °C/min). **b**, Biolayer interferometry binding analysis of Neo2A binding to Neo2B. Biotinylated-Neo2A was immobilized on streptavidin-coated tips and analyzed for binding in the presence of serial dilutions of Neo2B (left panel) or Neo2A (right panel). The calculated KD between Neo2A and Neo2B is 4.6 μM. Colored lines represent the concentration of analytes in nM. **c**, Biolayer interferometry binding analysis of Neo2A binding to Neo2B in the presence of soluble hIL-2Rβ and hIL-2Rγ. Biotin-Neo2A was bound to Streptavidin-coated tips and analyzed for binding with serially diluted Neo2B in presence of the soluble IL-2 receptor subunits hIL-2Rβ and hIL-2Rγ. The calculated KD for the complex is 50.8 nM. Colored lines represent equimolar concentrations of Neo2B, hIL-2Rβ and hIL-2Rγ in nM.

**Fig. 1 |:**
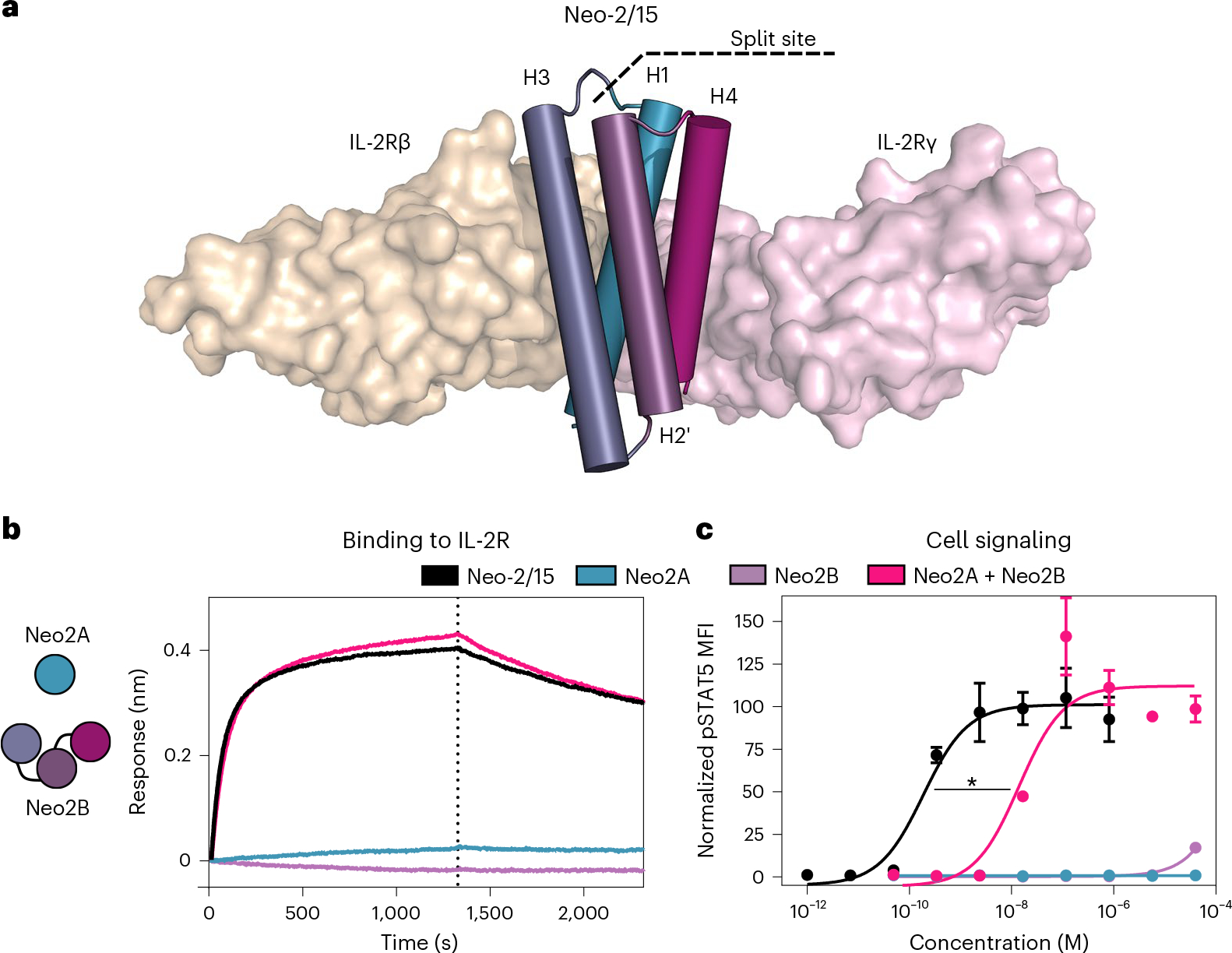
Neoleukin-2/15 can be split into two fragments that reconstitute its activity when combined. **a**, Splitting strategy for Neo-2/15. Structure of Neo2/15 (cylinder representation) and murine IL-2 receptors β and γ (beige and pink surface representations, respectively, PDB ID: 6dg5). Dashed line represents the selected split site between helix H1 (Neo2A), involved in the interface with both receptors, and fragment H32’4 (Neo2B). **b**, Binding of split Neo-2/15 fragments to the IL-2 receptor. Biolayer interferometry binding assay illustrating the binding kinetics of intact Neo-2/15 (black), Neo2A (blue), Neo2B (purple) and the combination of Neo2A + Neo2B (magenta) (1 μM concentration) binding to biotinylated human IL-2Rγ immobilized on an Octet streptavidin sensor in the presence of 250 nM soluble hIL-2Rβ. Full titrations are provided in Extended Data Fig. 1a. **c**, Signaling of split Neo-2/15 on human YT-1 cells. STAT5 phosphorylation in YT-1 cells following treatment with intact Neo-2/15 (black, EC_50_ = 1.87 × 10^−10^ M), Neo2A (blue, EC_50_ undetermined), Neo2B (purple, EC_50_ undetermined) or the combination of Neo2A + Neo2B (magenta, EC_50_ = 2.83 × 10^−8^ M). Treatment with the split pair reconstituted Neo-2/15 activity. * indicates different EC_50_ values with nonoverlapping 95% confidence interval ranges. Experiments were performed in triplicate three times, with similar results. Data are presented as mean ± s.d.

In the Neo-2/15 protein, helix H3 interacts with IL-2Rβ, H4 interacts with IL-2Rγ and H1 interacts with both receptors (Fig. 1a; the helical element numbering corresponds to that in IL-2; the sequence order in Neo-2/15 is H1-H3-H2’-H4; H2’ does not have an IL-2 counterpart). We aimed to separate one of these helices from the others so that receptor heterodimerization could be achieved only in the presence of both fragments. We tested three pairs of split versions for Neo-2/15: (1) H1 and H3–H2’–H4, (2) H1–H3 and H2’–H4, and (3) H1–H3–H2’ and H4. In all three cases, much stronger IL-2 receptor binding and signaling was observed when two fragments were combined than for either fragment alone (Fig. 1b,c and Extended Data Fig. 1). For the split pair H1 and H32’4, neither fragment alone showed any activity even at high concentrations (Extended Data Fig. 1a). We selected this pair for subsequent studies, and the H1 and H32’4 split fragments are henceforth referred to as Neo2A and Neo2B, respectively. Further characterization of the fragments revealed that Neo2A is unfolded in solution while Neo2B remains helical (Extended Data Fig. 2a). The two split fragments have low affinity for each other in the absence of the IL-2 receptor subunits (dissociation constant (*K*_d_) ~4.5 μM) but form a much higher-affinity complex in the presence of the soluble IL-2Rβ and IL-2Rγ (*K*_d_ = 50.8 nM), a desirable property for ensuring on-target reconstitution (Extended Data Fig. 2b,c).

### Split Neo-2/15 is selectively reconstituted on target cells

**Extended Data Fig. 3 |:**
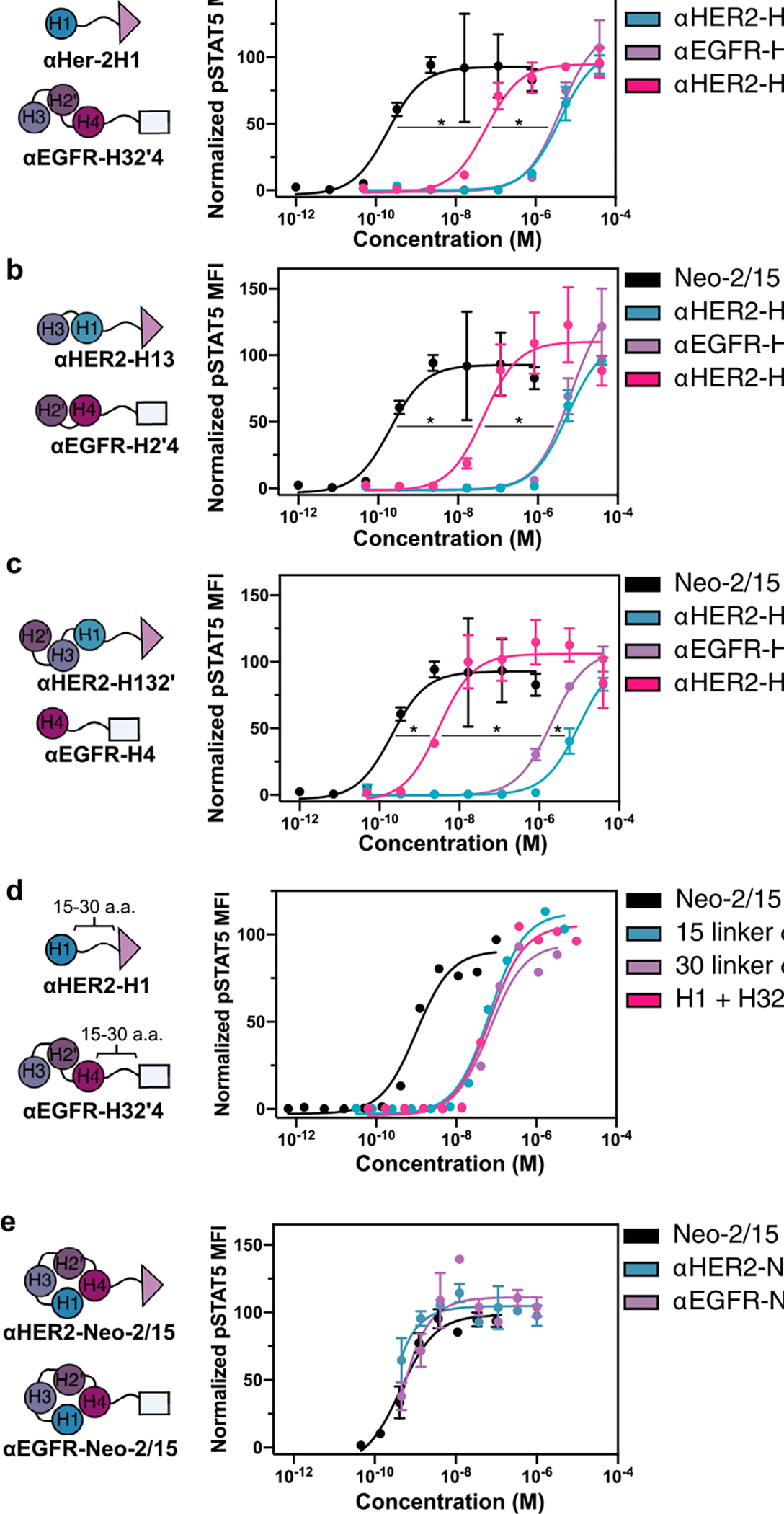
Immune cell signaling activity of targeted Split Neo-2/15 pairs. **a-c**, STAT5 phosphorylation response in YT-1 human NK cells following treatment with intact Neo-2/15 or split Neo-2/15 fragments fused to HER2- or EGFR-targeted DARPins. Results are shown for three split pairs: H1 + H32’4 (**a**); H13 + H2’4 (**b**); and H132’ + H4 (**c**). All Split Neo-2/15 variants remain functional after fusion to DARPin targeting domains. The calculated EC50 values are: (**a**) Neo-2/15 0.201 nM; ɑHER2-H1 4.09 μM; ɑEGFR-H132’ 4.20 μM; ɑHER2-H1 + ɑEGFR-H32’4 43.9 nM; (**b**) ɑHER2-H13 5.51 μM; ɑEGFR-H2’4 6.73 μM; ɑHER2-H13 + ɑEGFR-H2’4 43.9 nM; (**c**) ɑHER2-H132’ 9.87 μM; ɑEGFR-H4 2.03 μM; ɑHER2-H132’ + ɑEGFR-H4 3.07 nM; **d**, Increasing length of the linker separating the Neo-2/15 split fragments from the DARPin targeting domain from 15 to 30 amino acids does not affect activity. The calculated EC50 values are: Neo-2/15 0.99 nM; 15 residue linker ɑHER2-H1 + ɑEGFR-H32’4 68.3 nM; 30 residue linker ɑHER2-H1 + ɑEGFR-H32’4 68.1 nM; H1 + H32’4 67.8 nM. **e**, Intact Neo-2/15 fused to HER2- or EGFR-targeted DARPins retains full activity, as measured by STAT5 phosphorylation response in YT-1 human NK cells. Experiments were performed in triplicate three times with similar results. Data are presented as mean values +/− s.d. * indicates different EC50 values with non-overlapping 95% confidence interval ranges.

**Extended Data Fig. 4 |:**
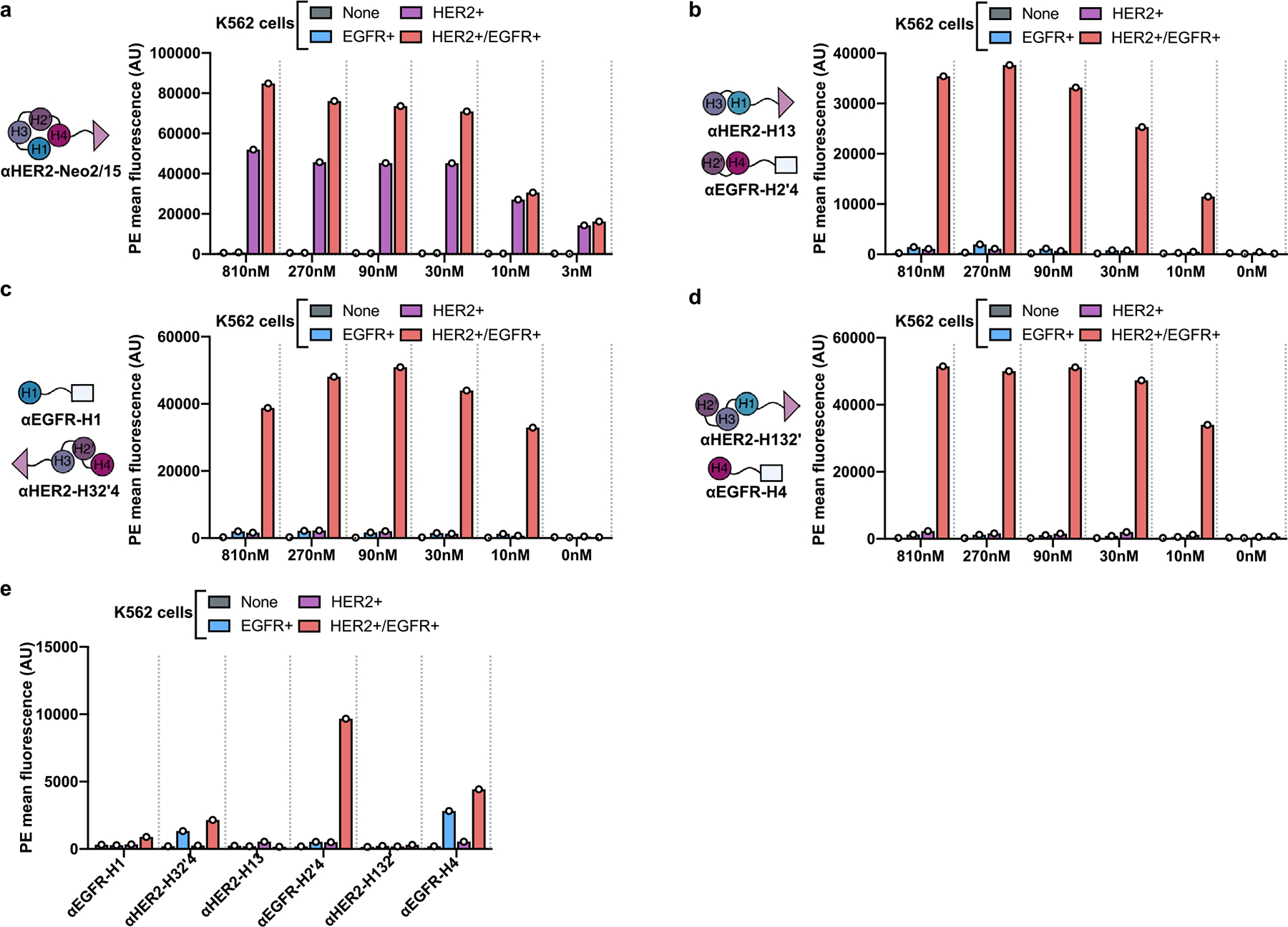
Reconstitution of Split Neo-2/15 targeted to the surface of transduced K562 cells. **a-c**, Dilution series of targeted Split Neo2/15 variants were evaluated by flow cytometry using the experimental setup shown in Fig. 2a. Reconstitution of IL-2 receptor binding activity is measured by recruitment of fluorescently-labeled hIL-2Rβγ (resulting from a mixture of IL-2Rβ, biotinylated IL-2Rγ, and PE-conjugated streptavidin). Data are shown for HER2-targeted intact Neo-2/15 (**a**) and three split pairs: ɑHER2-H13 + ɑEGFR-H2’4 (b); ɑEGFR-H1 + ɑHER2-H32’4 (that is, ɑEGFR-Neo2A and ɑHER2-Neo2B (**c**); and ɑHER2-H132’ + ɑEGFR-H4 (**d**). **e**, Activity of the individual split Neo-2/15 fragment fusion proteins (810 nM concentration) was tested to evaluate the potential for off-target activity. The dilution series were performed in a single replicate (n = 1). All data are presented as mean values.

**Fig. 2 |:**
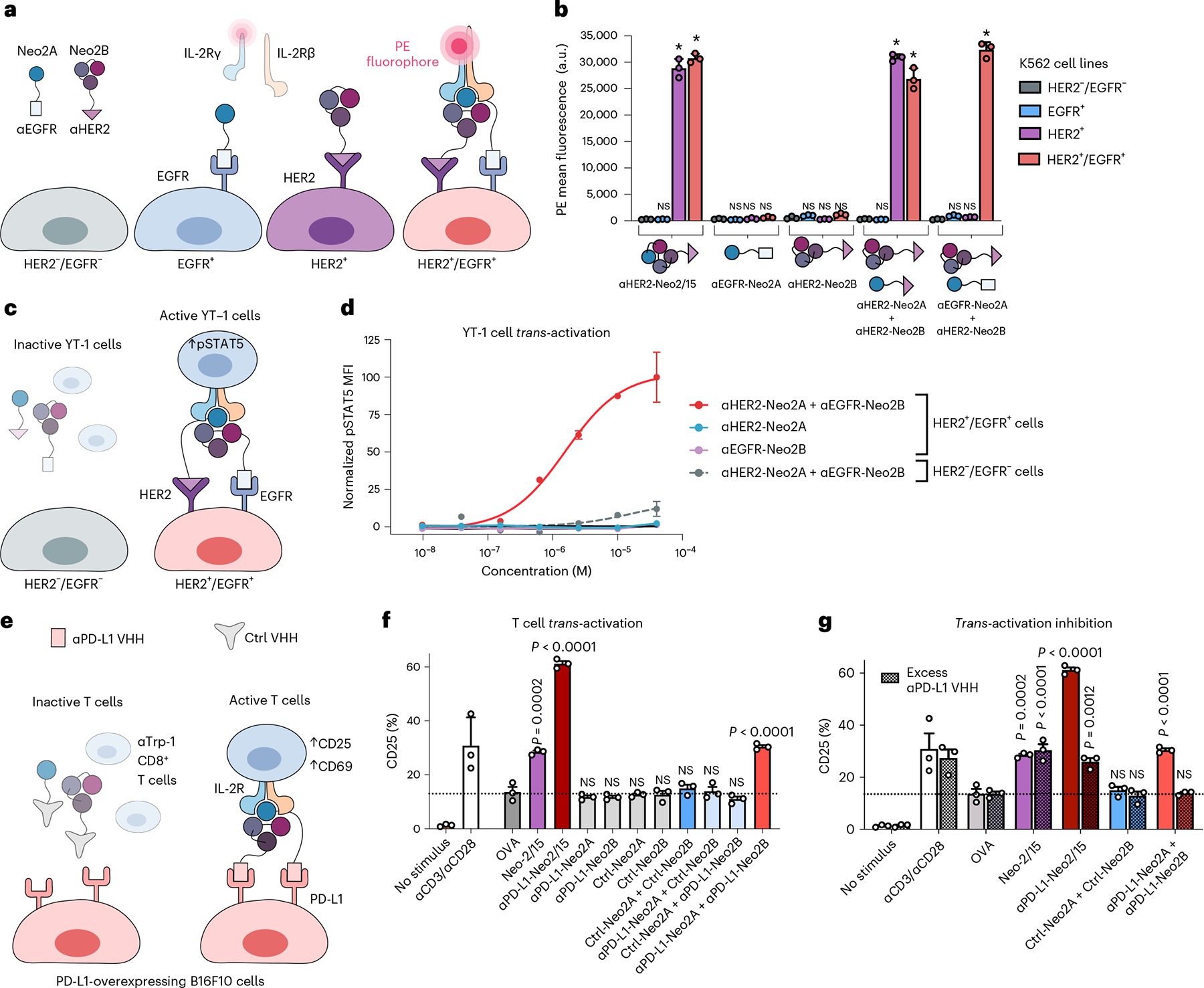
*Trans*-activation of immune cells through targeted reconstitution of split Neo-2/15 on the surface of tumor cells. **a**,**b**, In vitro assay for reconstitution of Split Neo-2/15 on the surface of engineered K562 cells. **a**, Neo2A and Neo2B split proteins were fused to anti-EGFR and anti-HER2 DARPin-targeting domains. K562 cells (gray) and engineered K562 cell lines transduced with HER2-eGFP (purple), EGFR-iRFP (blue) or both (pink) were mixed in an equivalent ratio then incubated with targeted split Neo-2/15 proteins at 10 nM. **b**, Reconstitution of Neo-2/15 binding activity on the cell surface was measured by recruitment of phycoerythrin (PE)-labeled soluble hIL-2Rβγ. *P < 0.0001. **c**,**d**, Assay performed to measure *trans*-presentation of split Neo-2/15 on the surface of K562 tumor cells to human YT-1 NK cells. **c**, Untransduced HER2^−^/EGFR^−^ K562 cells (off-target) or HER2^+^/EGFR^+^ K562 cells (on-target) were cocultured with YT-1 cells in a 20:1 ratio (K562:YT-1) in the presence of ɑHER2-Neo2A and ɑEGFR-Neo2B fusion proteins. **d**, YT-1 cell activation was analyzed by measurement of STAT5 phosphorylation. Strong signaling was observed for on-target K562 cells incubated with ɑHER2-Neo2A + ɑEGFR-Neo2B (EC_50_ = 1.57 × 10^−6^). The remaining groups showed weak signaling (undetermined EC_50_). **e**,**f**, Assay used to detect *trans*-activation of antigen-specific αTrp-1 CD8^+^ T cells by split Neo-2/15 on the surface of B16F10 melanoma cells overexpressing PD-L1 in coculture. **e**, Split Neo-2/15 proteins were fused to either a nanobody (VHH) with irrelevant specificity (Ctrl) or an αPD-L1 nanobody. **f**, *Trans*-activation of T cells was measured by expression of the activation marker CD25. All samples were incubated with an ɑCD28 antibody to provide costimulation. αCD3 was used as a positive control for T cell activation, and OVA peptide to quantify basal CD25 expression levels. **g**, B16 and CD8^+^ T cells were cocultured in the presence of activating proteins. Addition of excess soluble ɑPD-L1 nanobody (VHH) to competitively inhibit binding of targeted split fusion proteins to the B16 cell surface resulted in attenuated T cell activation. All data in the figure are presented as mean ± s.d. One-way ANOVA comparisons against the negative control group. NS, no statistical significance; a.u., arbitrary units.

We next sought to evaluate whether selective targeting of split Neo2/15 components to the surface of cells could reconstitute activity at submicromolar concentrations through colocalization. We fused the fragments to designed ankyrin repeat proteins (DARPins) that target the extracellular domains of HER2 (G3 DARPin^28^) and EGFR (E01 DARPin^29^), two well-known tumor-associated antigens. Intact Neo-2/15 and the split fragments thereof retained their function after either N- or C-terminal fusion to the targeting domains and irrespective of linker length (Extended Data Fig. 3a–e). The targeted split fragments were added to a mixture of four K562 cell lines engineered to express EGFR-iRFP, HER2-eGFP, both or neither^30^ (Fig. 2a), and reconstitution of Neo-2/15 activity on the surface of target cells was measured by recruitment of the soluble IL-2Rβγ complex (Fig. 2a). IL-2Rβγ was specifically recruited to HER2^+^ cells when both Neo2A and Neo2B split fragments were targeted to HER2 (αHER2-Neo2A + αHER2-Neo2B), with similar activity to HER2-targeted intact Neo-2/15 (αHER2-Neo2/15), demonstrating colocalization-dependent IL-2 receptor binding (Fig. 2b and Extended Data Fig. 4a). IL-2Rβγ was also recruited with high selectivity to the surface of double-positive HER2^+^/EGFR^+^ when one split fragment was targeted to HER2 and the other to EGFR (Fig. 2b and Extended Data Fig. 4b), demonstrating the feasibility of this approach in targeting two different antigens on the same cell surface (AND logic-gated activity). Similar selectivity was observed for other split pair variants (Extended Data Fig. 4c,d) but, at high concentrations, one of the single fragments showed residual receptor-binding capacity (Extended Data Fig. 4e).

### Split Neo-2/15 *trans*-activates immune cells

**Extended Data Fig. 5 |:**
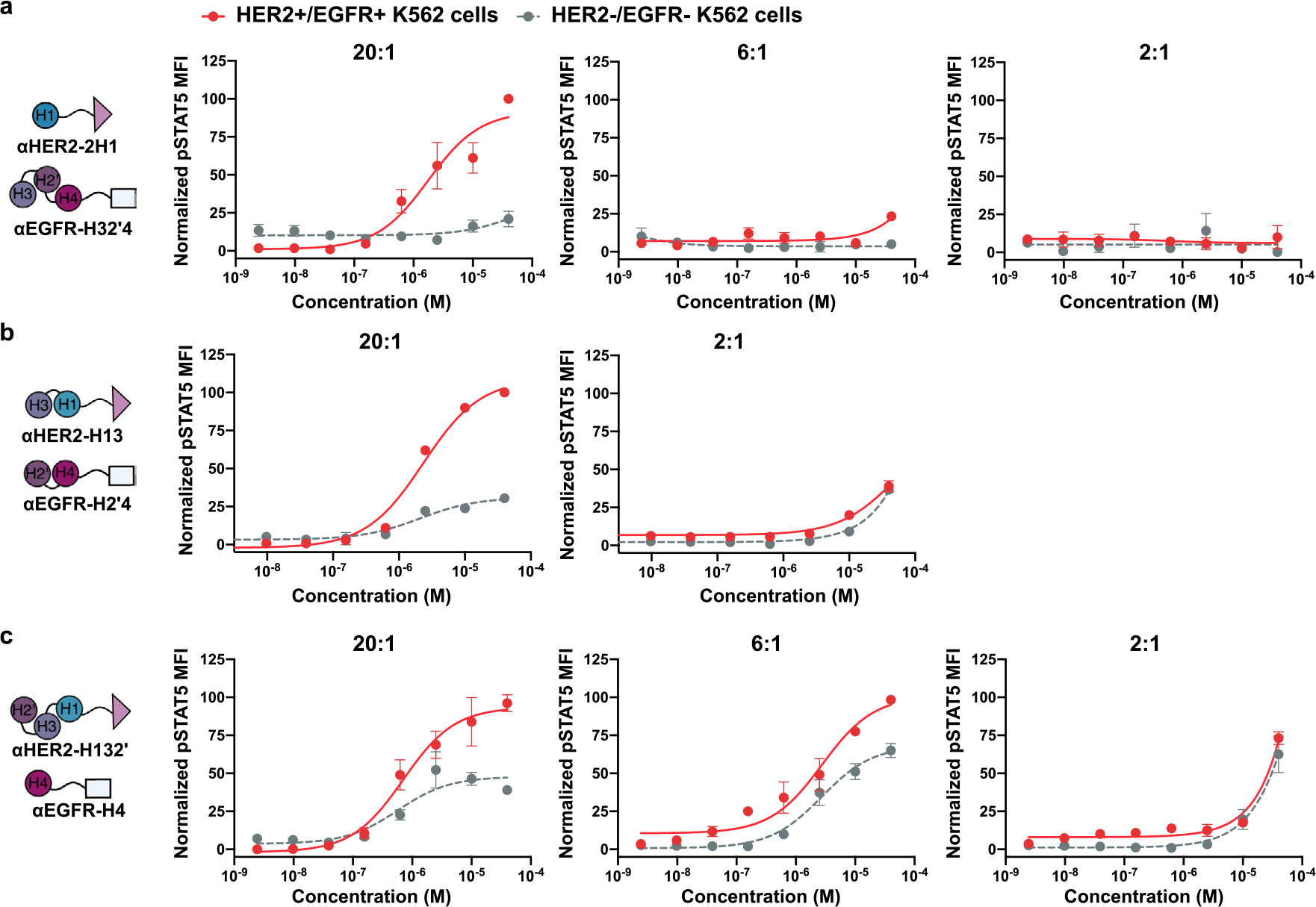
Trans-presentation of split-Neo-2/15 on the surface of K562 to YT-1 cells has limited potency in the absence of an immunological synapse. Untransduced HER2-/EGFR-K562 (off-target) cells or double-positive HER2 + /EGFR + K562 (on-target) cells were cocultured with YT-1 human NK cells in varying K562:YT-1 cell ratios in the presence of ɑHER2-Neo2A and ɑEGFR-Neo2B or other split Neo-2/15 fragment pairs at varying concentrations. STAT5 phosphorylation of YT-1 cells was observed for high K562:YT-1 cell ratios, demonstrating trans-activation of immune cells from the surface of targetexpressing cells. The experiments were performed in triplicate three times with similar results. Data are presented as mean values +/− s.d.

To evaluate whether the targeted split Neo-2/15 proteins could *trans*-activate immune cells when bound to target tumor cells, we first targeted the Neo2A and Neo2B split fragments to the double-positive HER2^+^/EGFR^+^ K562 cell line, described above, in the presence of an IL-2-responsive human natural killer (NK) cell line^31^ (YT-1) and assessed STAT5 phosphorylation as a readout for IL-2 signaling (Fig. 2c). Selective activation was observed for the targeted Neo2A–Neo2B pair (Fig. 2d and Extended Data Fig. 5a) but required a high ratio (20:1) of tumor cells to immune cells. We hypothesized that more potent *trans*-activation could occur for antigen-specific immune cells because the immune synapse between the immune cell and tumor cell would bring them into close proximity for an extended period of time, allowing the targeted cytokine more efficiently to dimerize the receptor subunits and activate the immune cell. To test this hypothesis, we fused each of the split Neo-2/15 fragments to the αPD-L1 nanobody B3^32,33^ and targeted them to B16F10 (also referred to herein as B16) melanoma cells overexpressing PD-L1 ^34^. Tumor cells were cocultured overnight with melanoma antigen-specific αTrp-1 CD8^+^ T cells (Fig. 2e) in the presence of an αCD28 antibody to provide costimulation. The split Neo-2/15 fragments targeted to the surface of B16 cells efficiently *trans*-activated the antigen-specific T cells (Fig. 2f and Supplementary Fig. 1a). This activation was dependent on targeting: fusions to a control nanobody with irrelevant specificity (1B7, which recognizes a *Toxoplasma gondii* kinase^35^) did not activate T cells, and addition of an excess of soluble ɑPD-L1 nanobody to outcompete cell surface binding considerably reduced *trans*-activation (Fig. 2g and Supplementary Fig. 1b).

### Split Neo-2/15 reduces toxicity in murine models

**Fig. 3 |:**
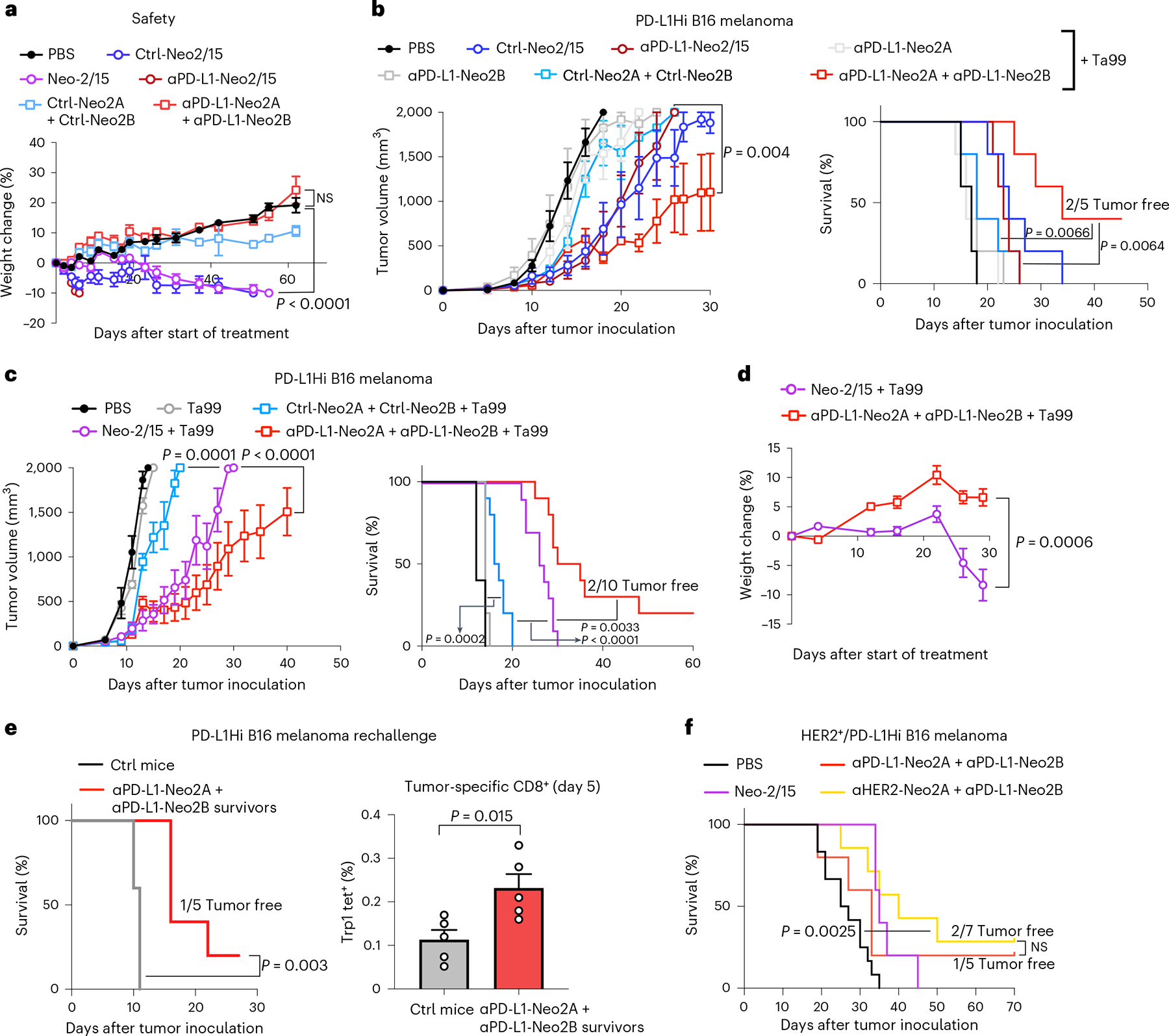
Targeting split Neo-2/15 to tumors increases safety and enhances antitumor efficacy. **a**, Safety study in immunocompetent mice (*n* = 5 per group) treated with targeted Neo-2/15 and targeted split Neo-2/15. “Ctrl” indicates fusion to an irrelevant nanobody as untargeted control. Mice were treated daily with equivalent doses of the indicated proteins (2.6 nmol). Weight change was monitored to evaluate toxicity of the dosed molecules. One-way ANOVA was used to compare weights at day 60. **b**, Efficacy study in immunocompetent mice (*n* = 5 per group) bearing PD-L1-overexpressing B16 melanoma cells. Mice were administered daily with therapeutic doses of the test items: αPD-L1-Neo2/15 and Ctrl-Neo2/15 at 430 nmol or targeted split Neo-2/15 fragments at 8 nmol. All groups were cotreated with Ta99 biweekly. Unpaired two-tailed *t*-tests of tumor growth values at day 22. **c**, Efficacy study in mice bearing B16 PD-L1-overexpressing melanoma cells in the flank (*n* = 5 for PBS and Ta99 groups, *n* = 10 for other groups). Neo-2/15 was dosed daily at 2.6 nmol and split Neo-2/15 fusions at 8 nmol daily. **d**, Weight change of the mice in the study shown in **c** comparing the toxicity of Neo2/15 and the αPD-L1 split Neo-2/15 fusions. **c**,**d**, Unpaired two-tailed Student’s *t*-tests were performed to compare tumor volume and weight change at days 19 and 29. **e**, Surviving αPD-L1 split Neo-2/15-treated mice were rechallenged alongside control mice receiving a primary challenge (*n* = 5). CD8^+^ T cells from treated mice were analyzed for recognition of tumor cells by the presence of anti-Trp1-MHCI tetramer (right). **f**, Survival curves for immunocompetent mice bearing B16 melanoma cells in the flank overexpressing murine PD-L1 and human HER2 and dosed daily with the following test items: PBS (*n* = 12), Neo-2/15 (2.6 nmol, *n* = 5), ɑPD-L1-Neo2A + ɑPD-L1-Neo2B (8 nmol, *n* = 5), ɑHER2-Neo2A + ɑPD-L1-Neo2B (8 nmol, *n* = 7). Statistical analysis for survival curves was performed by Mantel–Cox log rank. Data presented as mean ± s.e.m.

We next sought to evaluate the safety profile and therapeutic activity of split Neo-2/15. IL-2 immunotherapy induces toxicities such as pulmonary edema and weight loss in mice^3,22^. We previously showed that treatment with Neo-2/15 has lower systemic toxicity than IL-2 in preclinical models^3,22^. We hypothesized that the conditional activity of split Neo-2/15 could further expand the therapeutic window by preventing systemic activity of the individual fragments until colocalized to the desired site. We treated healthy C57BL/6 J mice with equimolar doses of Neo-2/15, targeted and untargeted Neo-2/15 fusion proteins (ɑPD-L1-Neo2/15 and Ctrl-Neo2/15) and targeted and untargeted split Neo-2/15 fusion proteins (Fig. 3a) at a daily dose of 2.6 nmol (equivalent to 30 μg of Neo-2/15, higher than previously reported and anticipated to show moderate toxic effects^22^). Signs of toxicity were evident in mice treated with Neo-2/15 and Ctrl-Neo2/15 at 23 days post treatment due to the high dose (Fig. 3a and Supplementary Fig. 2). The ɑPD-L1-Neo-2/15 fusion protein was found to be toxic just days into treatment, possibly due to accumulation by unwanted localization to PD-L1-expressing cells such as myeloid dendritic cells and brown adipocytes^32^. As anticipated, neither the untargeted nor targeted split Neo-2/15 fragment combinations showed any signs of toxicity at this dose.

### Targeted split Neo-2/15 induces potent antitumor effects

We evaluated the antitumor effects of split Neo-2/15 in syngeneic murine models of PD-L1-overexpressing B16 melanoma tumors^35^. First, we compared the therapeutic effect of intact Neo-2/15 fusion proteins (untargeted or targeted to PD-L1) with that of the split Neo-2/15 fusion protein (untargeted or targeted to PD-L1). Therapeutic molecules were administered at the highest nontoxic dose—that is, intact Neo-2/15 fusions controls (ɑPD-L1-Neo-2/15 and Ctrl-Neo-2/15) at 12 μg per mouse per day (430 pmol), whereas split fragments were dosed at a ~20-fold-higher molar ratio (200 μg per mouse per day, 8 nmol). All proteins were administered intraperitoneally except for the Neo2B fusions, which were administered subcutaneously in the flank opposite to that of the tumor to prevent potential association of split molecules before injection. Molecules were administered either as single-agent therapeutics (Supplementary Fig. 3a) or in combination with TA99 antibody (which targets surface Trp1 on melanocytes), which is widely used in combination with IL-2 in the poorly immunogenic B16 melanoma model^36^ (Fig. 3b). Survival and tumor growth were evaluated for all groups. As expected, individual fragments did not show any antitumor therapeutic activity. The antitumor efficacy of the PD-L1-targeted split fragments (ɑPD-L1-Neo2A + ɑPD-L1-Neo2B) was superior to that of the untargeted split fragments (Ctrl-Neo2A + Ctrl-Neo2B), as well as to that of the targeted and untargeted intact Neo-2/15 fusion proteins, resulting in extended survival and complete tumor clearance in two mice (Fig. 3b). Consistent with these efficacy results, ɑPD-L1-Neo2A + ɑPD-L1-Neo2B therapy resulted in expansion and activation of CD8^+^ T cell expansion in peripheral blood (Supplementary Fig. 3c,d). Intact Neo-2/15 fusions (ɑPD-L1-Neo-2/15 and Ctrl-Neo-2/15) did not show a potent antitumor effect due to dose-limiting toxicity, as predicted by the safety studies presented in Fig. 3a, and performed similarly regardless of targeting, in agreement with previous reports, showing that efficacy of a targeted, fully active IL-2-mimetic molecule is mainly mediated by systemic activity^6^.

We next carried out a more extensive comparison of the antitumor efficacy of intact Neo-2/15 compared with untargeted and targeted split Neo-2/15 (Fig. 3c). The ɑPD-L1-Neo2A + ɑPD-L1-Neo2B molecules showed little toxicity and exhibited superior efficacy and safety compared with intact Neo-2/15 (Fig. 3d). This enhanced therapeutic effect was probably a result of increased on-tumor accumulation of reconstituted active Neo-2/15 due to a higher tolerated dose and reduced systemic effects when administered as a targeted split molecules. In the case of mice treated with untargeted Ctrl-Neo2A + Ctrl-Neo2B at the high 8-nmol dose, we observed signs of systemic toxicity, further highlighting the benefit of targeting conditionally active cytokines to concentrate their activity at the tumor site. Remarkably, among all treatment groups, the cohort treated with targeted split Neo-2/15 (ɑPD-L1-Neo2A + ɑPD-L1-Neo2B) was the only one in which some mice achieved complete tumor remission. We evaluated the durability of this effect by rechallenging the ‘cured’ mice with new B16 tumors (Fig. 3e). Rechallenged mice showed improved survival (one mouse again achieved complete remission) and had higher numbers of tumor-specific (anti-Trp1) CD8^+^ T cells compared with mice receiving a primary challenge, showing modest evidence of immunologic memory consistent with other IL-2 based therapeutics. Overall, these results demonstrate the therapeutic potential of the targeted split Neo-2/15 molecule, highlighting its ability to overcome dose-limiting toxicities often observed in cytokine-based therapeutics.

### AND logic-gated split Neo-2/15 shows in vivo activity

**Extended Data Fig. 6 |:**
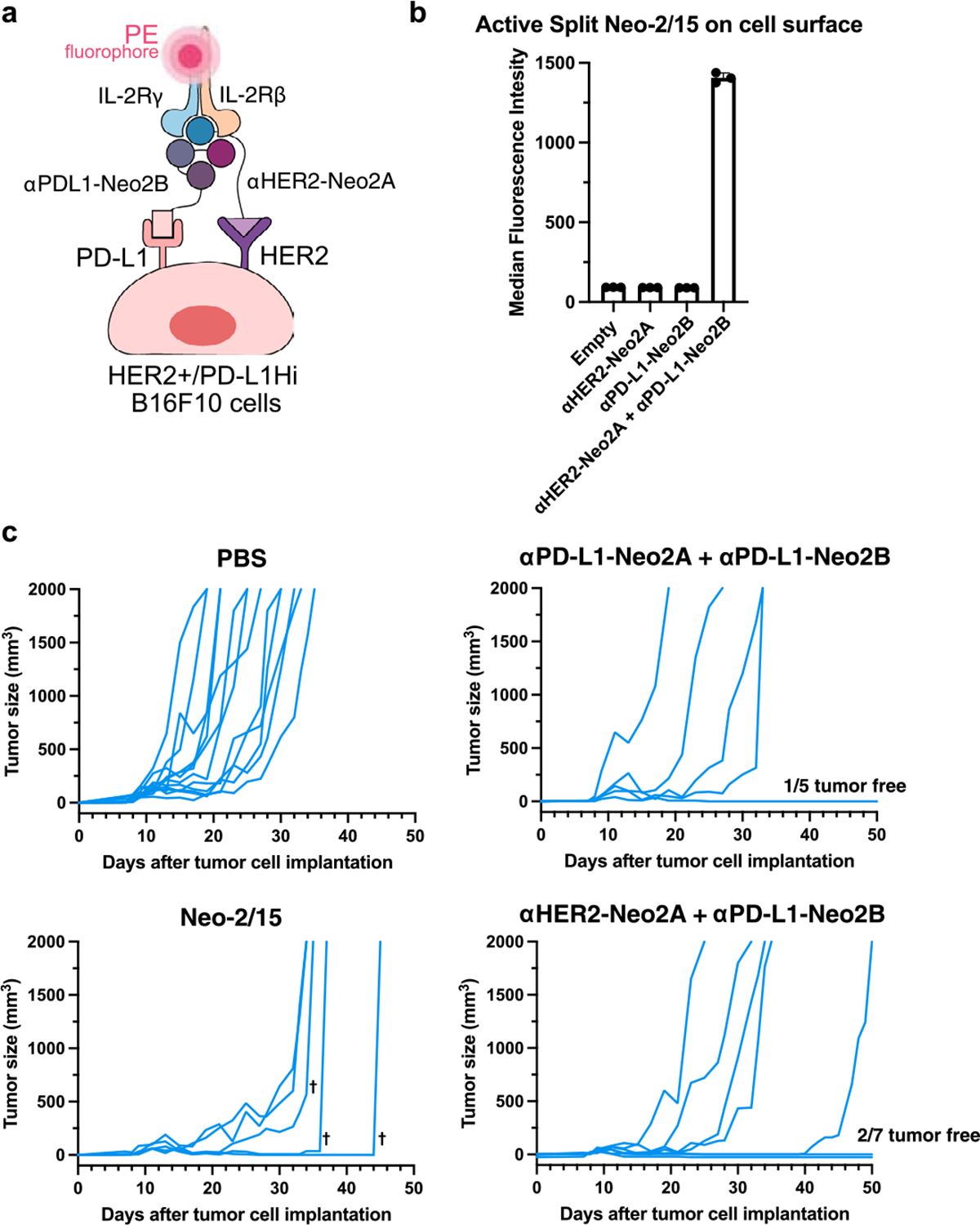
Split Neo-2/15 shows “AND” logic-gated targeted activity on B16F10 tumor cells overexpressing HER2 and PD-L1. **a**, Depiction of the *in vitro* assay to determine reconstitution of Split Neo-2/15 binding activity on the surface of B16F10 melanoma cells overexpressing human HER2 and murine PD-L1. Reconstitution of Neo-2/15 binding activity on the cell surface is measured by recruitment of PE-labeled hIL-2Rβγ. Data are presented as mean values +/− s.d. **b**, Quantification of Split Neo-2/15 reconstitution on the surface of Her2+/PD-L1Hi B16F10 melanoma cells (n = 3). Combination of αHER2-Neo2A and αPD-L1-Neo2B effectively reconstituted Split Neo-2/15 activity, demonstrating AND logic-gated activity on the engineered B16F10 cells. **c**, Individual tumor growth data for the efficacy study in mice bearing B16 melanoma cells overexpressing murine PD-L1 and human HER2 shown in Fig. 3f. PBS (n = 12), Neo-2/15 (2.6 nmol, n = 5), ɑPD-L1-Neo2A + ɑPD-L1-Neo2B (8 nmol, n = 5), ɑHER2-Neo2A + ɑPD-L1-Neo2B (8 nmol, n = 7). $\dot{\text{T}}$ indicates surviving mice euthanized due to toxicity when the body condition score of the mice was less than or equal to two in accordance with IACUC Standard Procedure.

A key advantage of a two-component conditionally active system is that activity can be targeted to cells that express a specific combination of two surface markers. We set out to explore the feasibility of this approach in mouse tumor models. To do so, we transfected PD-L1-overexpressing B16 melanoma tumors with the full extracellular domain of human HER2 as a second surface marker (Supplementary Fig. 4). We confirmed that the targeted split constructs bound and reconstituted IL-2 activity on the surface of this newly engineered cell line by measuring recruitment of the soluble IL-2Rβγ complex (Extended Data Fig. 6a,b). Immunocompetent mice were then inoculated with these cells and treated with various Neo-2/15 constructs: intact Neo-2/15, the previously tested PD-L1-targeted split fragments (ɑPD-L1-Neo2A + ɑPD-L1-Neo2B) and a combination of split fragments targeting PD-L1 and HER2 (ɑHER2-Neo2A + ɑPD-L1-Neo2B). Because HER2 is a human protein, TA99 was not administered as cotherapy to reduce spontaneous immunoreactivity against the tumor cell. All groups showed antitumor activity (Fig. 3f and Extended Data Fig. 6c) and, remarkably, both cohorts including split fragments had complete tumor remission with no major signs of toxicity. On the other hand, some Neo-2/15-treated mice showed signs of systemic toxicity (Extended Data Fig. 6c). These results demonstrate that the split system can elicit logic-gated immunotherapeutic activity on tumor cells that express a specific combination of two surface antigens.

### Split Neo-2/15 activates at high surface antigen density

**Extended Data Fig. 7 |:**
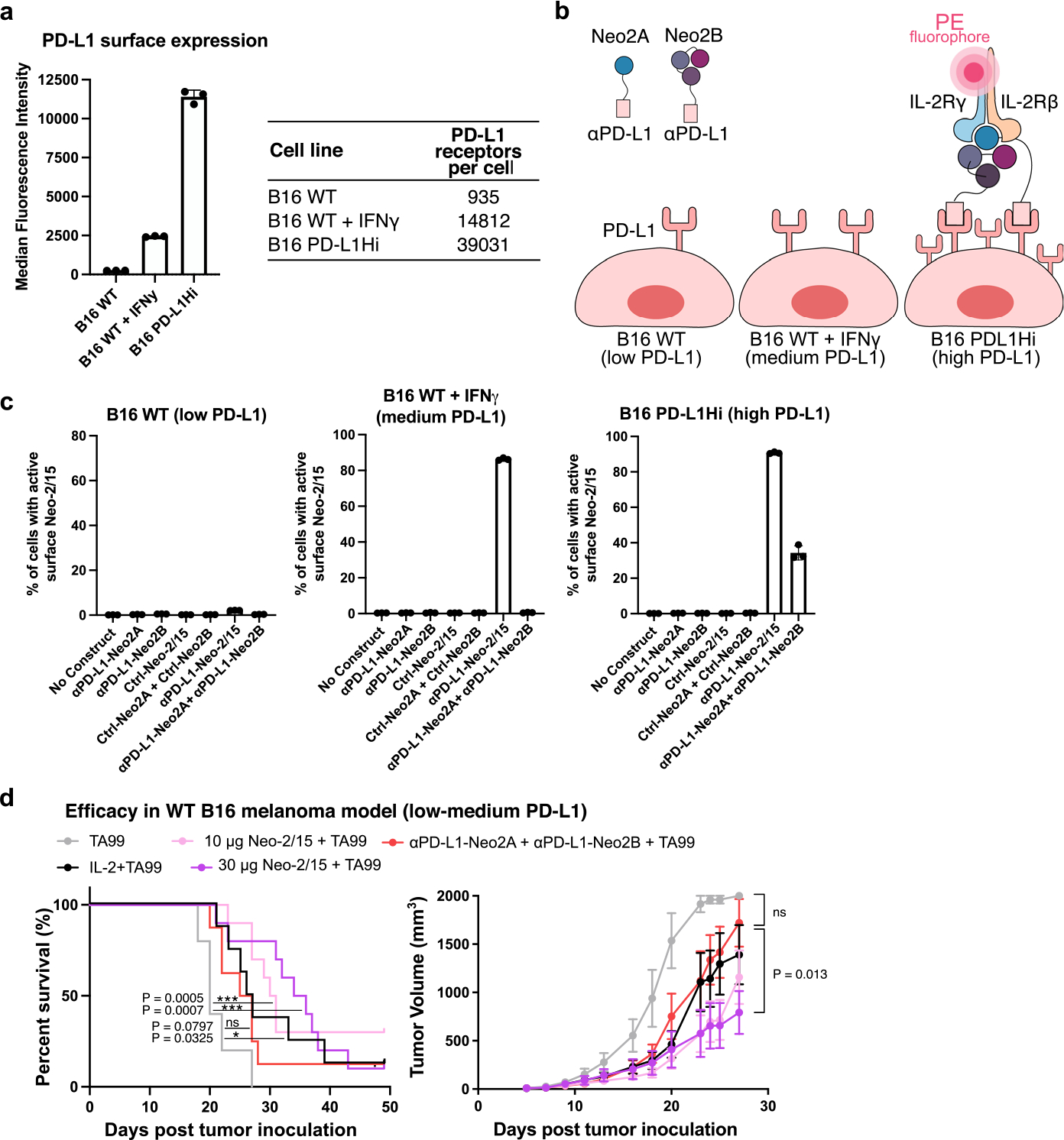
Activity of targeted Split Neo-2/15 is restricted to cells with high surface PD-L1 expression. **a**, Quantitation of murine PD-L1 expression on B16F10 melanoma cell lines. B16F10 WT melanoma cells are low PD-L1 expressors, B16F10 WT stimulated with IFNγ are medium expressors, engineered B16 PD-L1Hi cells are high expressors. Quantitation was performed using QuantiBRITE phycoerythrin beads. n = 3 technical replicates. Data are presented as mean values +/− s.d. **b**, Depiction of the *in vitro* assay to determine reconstitution of Split Neo-2/15 binding activity on the surface of B16F10 melanoma cells with different levels of PD-L1 expression. PD-L1-targeted Neo2A and Neo2B fragments are only reconstituted on the surface of B16 cells with high PD-L1 receptor counts. Reconstitution of Neo-2/15 binding activity on the cell surface is measured by recruitment of PE-labeled hIL-2Rβγ. **c**, Quantification of Split Neo-2/15 binding activity on the surface of B16F10 melanoma cells with different levels of PD-L1 expression. Active Neo-2/15 on the cell surface is measured by recruitment of PE-labeled hIL-2Rβγ. Intact αPD-L1-Neo2/15 showed IL-2Rβγ binding activity on the surface of cells that were intermediate and high expressors, whereas the αPD-L1-Neo2A + αPD-L1-Neo2B were only able to reconstitute on high expressor cells. n = 3 technical replicates. Data are presented as mean values +/− s.d. **d**, Efficacy study in C57BL/6 J mice (n = 5/group) bearing WT B16F10 melanoma cells in the flank. Mice were dosed daily with therapeutic doses of mIL-2 (13 μg/mouse), Neo-2/15 (10 μg/mouse and 30 μg/mouse) and αPD-L1-Neo2A + αPD-L1-Neo2B at 200 μg/mouse. All groups were co-treated with TA99 bi-weekly starting on day 3. Survival analyses performed via log-rank Mantel-Cox test. Tumor growth analyses were by unpaired, two-tailed t test. 3 early ulcerated tumors (<500 mm^3^) removed from this analysis.* indicates P < 0.05; ** indicates P < 0.01, *** indicates P < 0.001, ns = non significant. Data are presented as mean values +/− s.e.m.

The activation mechanism of split Neo-2/15 requires sufficient expression of surface markers on target cells for the two components to interact. We set out to understand in more detail the target antigen expression levels required to reconstitute active split Neo-2/15. First, we evaluated the activity of ɑPD-L1-Neo-2/15 and split Neo-2/15 on multiple B16F10 cell lines expressing different levels of PD-L1: B16 wild type (WT), B16 WT stimulated with IFN-γ (B16 cells upregulate PD-L1 in response to IFN-γ^32^) and engineered B16-overexpressing PD-L1. We quantified PD-L1 surface receptor levels for each cell line and confirmed that B16 WT (no IFN-γ) had low PD-L1 expression, B16 WT (+IFN-γ) had intermediate PD-L1 expression and engineered B16 cells had high PD-L1 expression (Extended Data Fig. 7a). Intact Neo-2/15 targeted to PD-L1 showed IL-2Rβγ-binding activity on the surface of cells that were intermediate and high PD-L1 expressors, whereas the split system showed activity only on high-expressor cells with surface receptor counts of 40,000 (Extended Data Fig. 7b,c). This steeper dependence on target receptor abundance is expected given that multiple binding events are required for reconstitution, and this feature of the split system can be exploited to target cells with high antigen density (that is, tumor cells) while sparing those with low antigen density (that is, healthy cells). To confirm these findings in vivo, we tested the efficacy in the syngeneic mouse B16F10 WT tumor model, which exhibits low to intermediate PD-L1 expression under physiological circumstances depending on factors such as endogenous expression of IFN-γ. As expected, mIL-2 and Neo-2/15 were efficacious due to their systemic activity (as shown previously^22^) whereas targeted split Neo-2/15 had less potent antitumor activity due to the low amount of surface PD-L1 in this model (Extended Data Fig. 7d).

**Extended Data Fig. 8 |:**
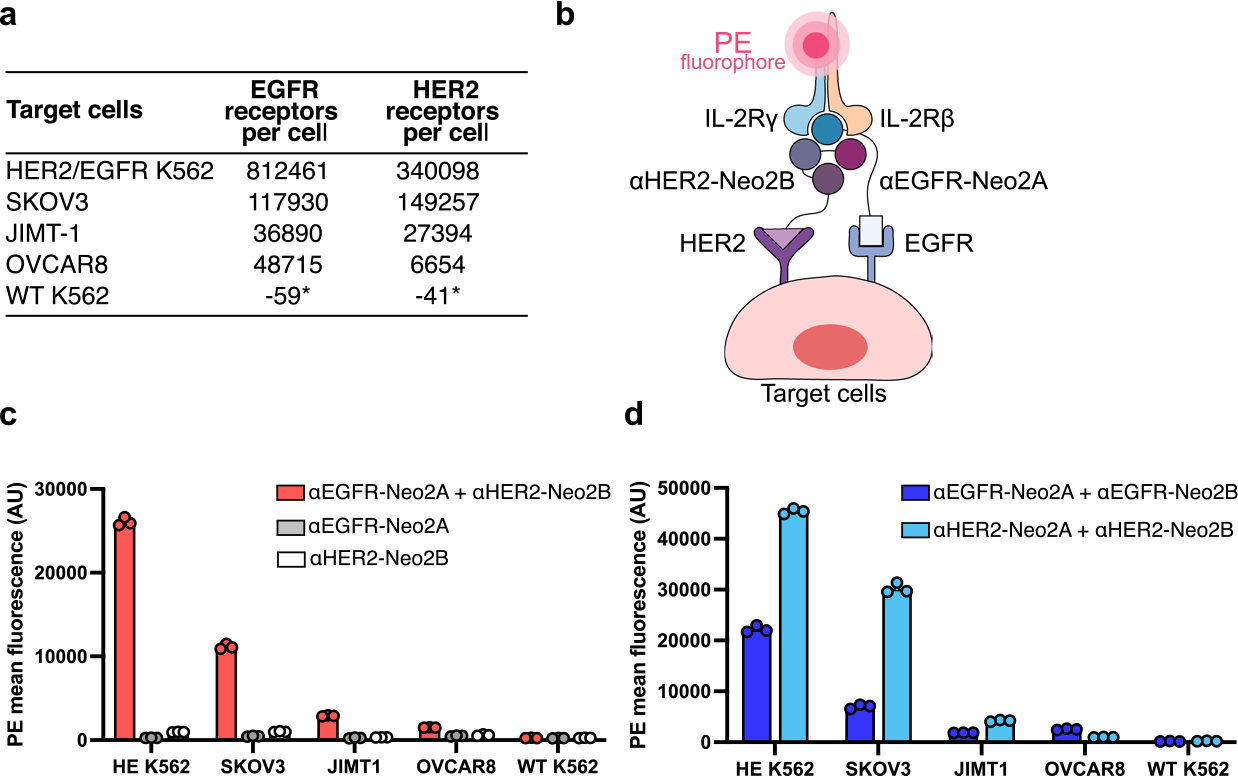
AND logic-gated targeted Split Neo-2/15 is active on human tumor cell lines with a wide range of surface receptor expression. **a**, Quantitation of human HER2 and EGFR surface expression levels on multiple target cell lines (SKOV3, JIMT-1, OVCAR8, WT K562 and engineered K562 overexpressing HER2 and EGFR). The quantitation was performed using QuantiBRITE phycoerythrin beads. (*) Indicates data reported in ref. ^30^. **b**, Depiction of the *in vitro* assay performed to determine reconstitution of AND logic-gated Split Neo-2/15 binding activity on the surface of target cell lines with diverse expression of EGFR and HER2. Reconstitution of Neo-2/15 binding activity on the cell surface is measured by recruitment of PE-labeled hIL-2Rβγ. **c**, Quantification of AND logic-gated Split Neo-2/15 activity on the surface of multiple human tumor cell lines. Combination αEGFR-Neo2A and αHER2-Neo2B effectively reconstituted Split Neo-2/15 activity, demonstrating AND logic-gated activity on the human tumor cell lines. n = 3 technical replicates. **d**, Split Neo-2/15 also showed activity when both Neo2A and Neo2B fragments were targeted to the same surface tumor antigen, that is, EGFR or HER2. n = 3 technical replicates. Data are presented as mean values +/− s.d

To determine whether nonengineered tumor lines express sufficiently high levels of tumor-associated surface antigens, we quantified HER2 and EGFR receptor levels on three human cell lines (Extended Data Fig. 8a) and targeted the split system to HER2 and EGFR (αHER2-Neo2A + αEGFR-Neo2B) (Extended Data Fig. 8b). Reconstitution of Neo-2/15 activity was measured by recruitment of the soluble IL-2Rβγ complex on the surface of target cells and was found to correlate with receptor amount (Extended Data Fig. 8b,c). Thus, the split system is effective for targeting of antigens at densities observed on human tumor cells.

### *Cis*-activation of immune cells with split Neo-2/15

**Extended Data Fig. 9 |:**
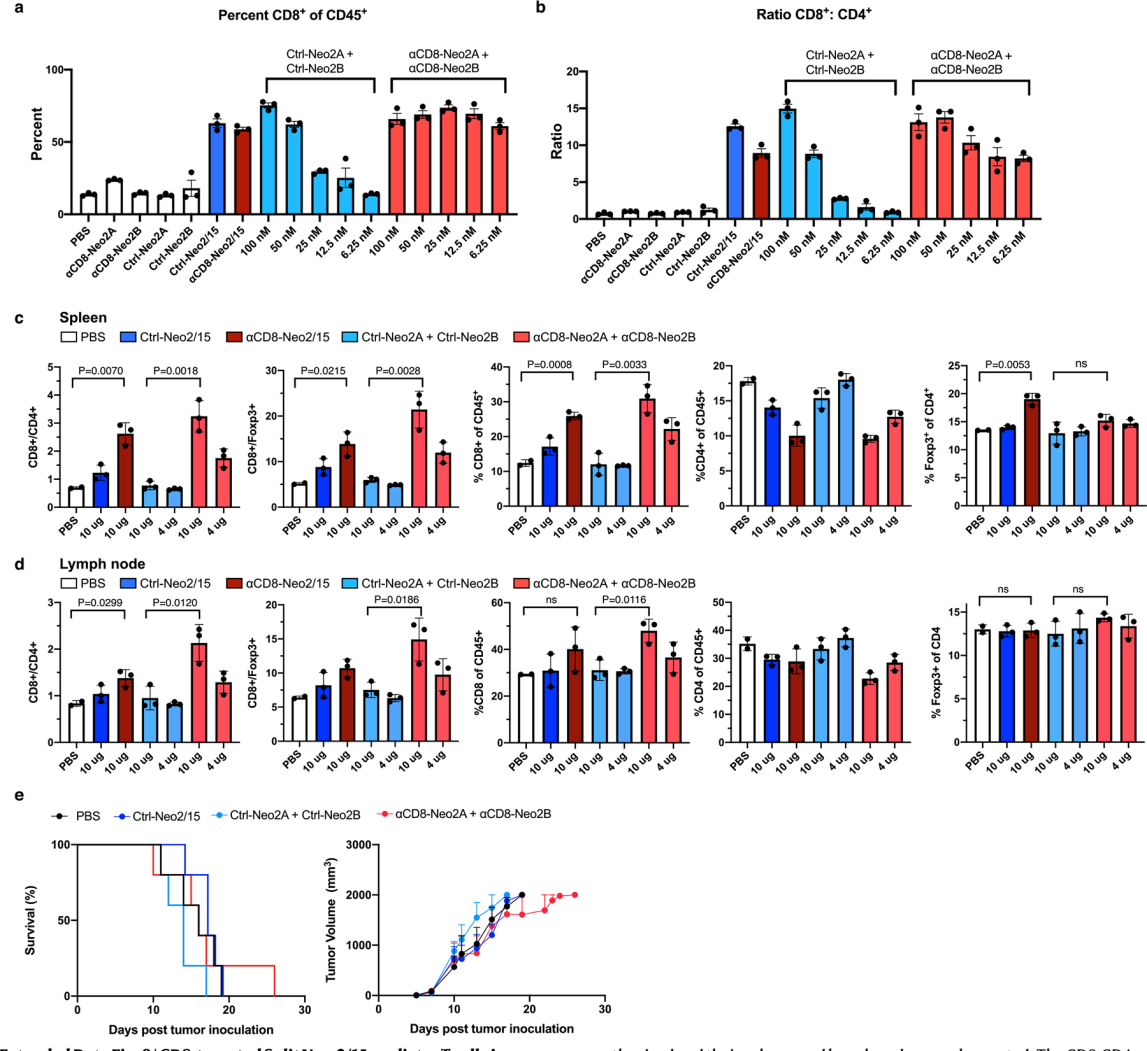
CD8-targeted Split Neo-2/15 mediates T cell *cis*-activation. **a-b**, Splenocytes from WT C57BL/6 J mice (n = 3) were cultured with the indicated Neo-2/15 and Split Neo-2/15 fusion proteins at the indicated concentrations (control constructs were used at 50 nM). After four days, cells were harvested and analyzed by flow cytometry. The change in the ratio of CD8:CD4 T cells was quantified to measure selective expansion of CD8 + T cells by the targeted constructs (**b)**. **c-d**, Healthy FoxP3-GFP mice (n = 3 mice, n = 2 for PBS control) were dosed daily with the indicated constructs. Intact Neo-2/15 fusions, Neo2B fusions, and PBS were administered intraperitoneally, while Neo2A constructs were given subcutaneously. After 5 days, the mice were euthanized and their spleens and lymph nodes were harvested. The CD8:CD4 ratios in the spleen cells (**c**) and lymph node cells (**d**) were quantified by flow cytometry to assess the extent of selective expansion of CD8 + T cells *in vivo*. Unpaired two-tailed Student’s *t*-test. ns = non significant. **e**, Anti-tumor efficacy of CD8-specific Split Neo-2/15 in a syngeneic mouse model of B16 melanoma. This efficacy experiment was carried out with the same conditions and in parallel to the study shown in Fig. 4c. Mice shown here were not co-treated with Ta99. Data in panels b-d are presented as mean values +/− s.d.; a and e (right panel) are presented as mean values +/− s.e.m.

**Fig. 4 |:**
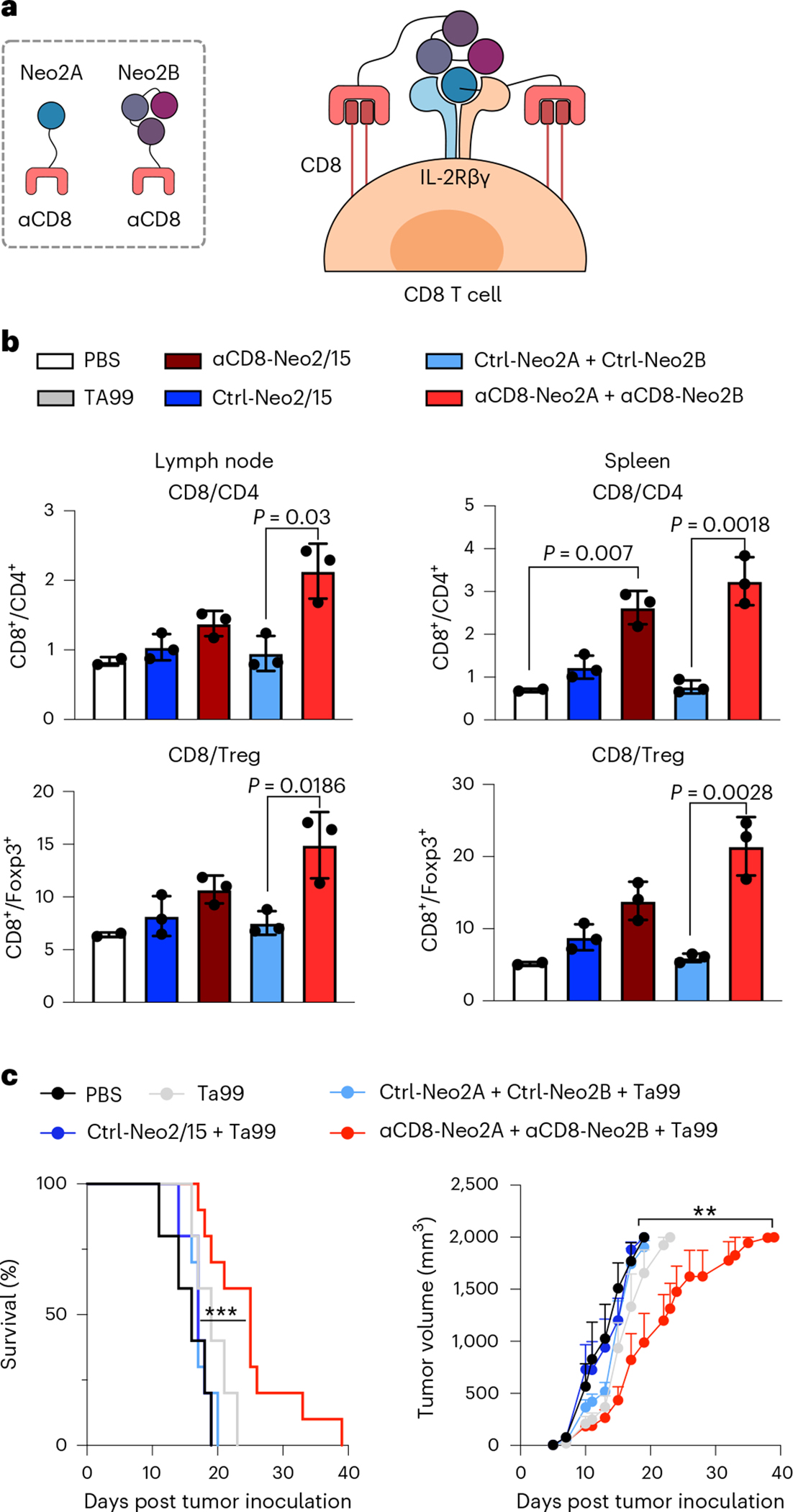
*Cis*-targeting split Neo-2/15 to CD8 specifically expands CD8^+^ T cells in murine models. **a**, *Cis*-activation mechanism of split Neo-2/15 targeted to CD8 (via fusion to an ɑCD8 VHH) on the surface of T cells. **b**, ɑCD8 split Neo2/15 promotes specific CD8^+^ T cell proliferation in healthy mice. Proteins were administered daily to nontumor-bearing Foxp3-GFP mice (*n* = 3; *n* = 2 for PBS control) for 5 days at 12 μg per mouse per day (500 pmol) for intact Neo-2/15 fusions and 10 μg per mouse per day (500 pmol) for split Neo-2/15 fusions. Cell populations of spleen and both inguinal lymph nodes collected at day 6 were analyzed by flow cytometry. Unpaired two-tailed Student’s *t*-test was used to evaluate statistical significance between groups. **c**, Efficacy study in C57BL/6 J mice bearing WT B16 melanoma treated with ɑCD8 split Neo-2/15 and TA99. Test items were dosed daily at 500 pmol each, starting on day 5. Ta99 was administered biweekly starting on day 3 (150 μg per mouse). Tumor growth (right) and mouse survival (left) are shown (*n* = 10 mice per group for ɑCD8-targeted split Neo-2/15-treated mice, *n* = 5 for controls). Left: ***P = 0.006, Mantel−Cox log rank comparing Ctrl-Neo2A + Ctrl-Neo2B with ɑCD8-Neo2A + ɑCD8-Neo2B. Right: unpaired two-tailed *t*-test comparing Ctrl-Neo2A + Ctrl-Neo2B with ɑCD8-Neo2A + ɑCD8-Neo2B at day 19. **P = 0.049. **b**,**c**, Data presented as mean ± s.e.m.

The results above illustrate the potential of split Neo-2/15 for targeting solid tumors to locally expand immune cell populations (*trans*-activation of immune cells). Some immunotherapeutic strategies benefit from cytokine-mediated systemic amplification of specific immune cell subtypes to promote antitumor effects (*cis*-activation)—for instance, potentiating pre-existing cytotoxic CD8^+^ T cells^37^ or chimeric antigen receptor T (CAR-T) cells in adoptive cell therapy applications^38^. Whereas unwanted activation and expansion of CD25^+^ regulatory T cells (Tregs) has limited many IL-2 based immunotherapies^7,39^, Neo-2/15 is CD25-independent and is thus not biased to preferentially activate CD25^+^ Tregs^22^, which is one of its main advantages as a cancer therapeutic over IL-2. To specifically target CD8^+^ cytotoxic T cells, we fused split Neo-2/15 proteins to an ɑCD8 nanobody-targeting domain^40^ (Fig. 4a). First, we confirmed the in vitro capacity of the resulting ɑCD8 split Neo-2/15 to selectively expand CD8^+^ T cells in cultures of splenocytes (containing both CD8^+^ and CD4^+^ T cells; Extended Data Fig. 9a,b). We then dosed ɑCD8 split Neo-2/15, ɑCD8 intact Neo-2/15 and untargeted controls in healthy Foxp3-GFP mice and assessed the extent of selective expansion of CD8^+^ T cells over other T cell subtypes such as Tregs (Fig. 4b and Extended Data Fig. 9c,d). We observed increased CD8^+^ frequency in lymph node and spleen in animals treated with ɑCD8-Neo2/15 and ɑCD8 split Neo-2/15. The cohort treated with targeted split Neo2/15 induced greater CD8^+^ T cell-specific proliferation compared with that treated with targeted intact Neo-2/15, probably due to reduced off-target effects of the conditionally active molecule. We then evaluated the antitumor efficacy of CD8-specific split Neo-2/15 in the B16 mouse model of melanoma. Intact Neo-2/15 and split fragment fusion proteins were dosed both as single agents (Extended Data Fig. 9e) and in combination with Ta99 (Fig. 4c). Consistent with our targeted activation studies, we observed delayed tumor growth and improved survival in mice treated with ɑCD8 split Neo-2/15 compared with both untreated mice and those treated with the untargeted split pair.

**Extended Data Fig. 10 |:**
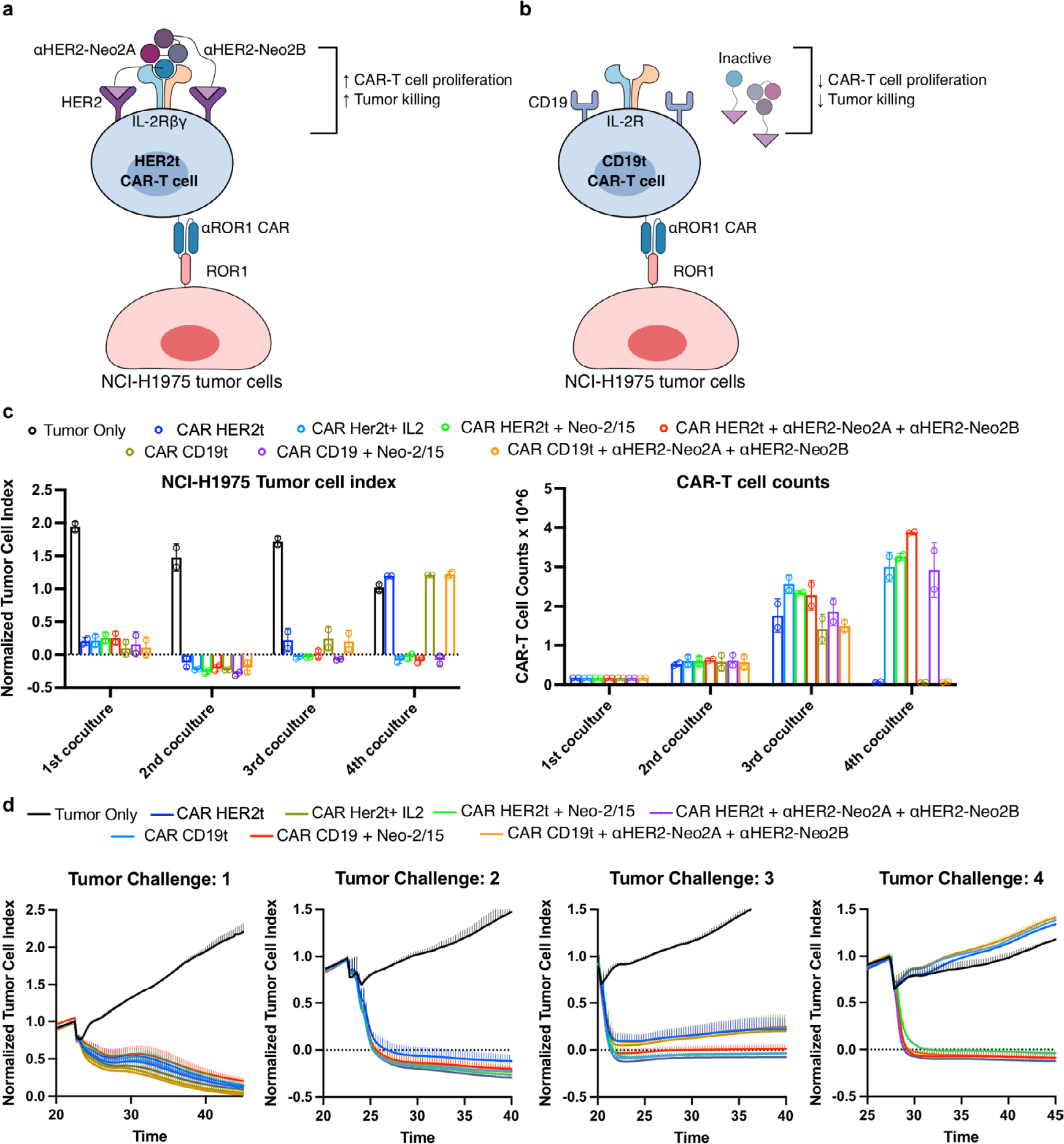
CAR-T cells elicit antitumor activity *in vitro* in the presence of cis-targeted Split Neo-2/15. **a-b**, Depiction of the assay performed to determine CAR-T cell proliferation and anti-tumor activity *in vitro*. αROR1 CAR-T cells were cocultured with NCI-H1975 tumor cells in presence or absence of *cis*-targeted Split Neo-2/15. CAR-T cells expressing HER2 as transfection marker (CAR HER2t) are effectively *cis*-activated by a combination of αHER2-Neo2A and αHER2-Neo2B, which promotes proliferation and anti-tumor activity (**a**). CAR-T cells expressing CD19 as transfection marker (CAR CD19t) are not *cis*-activated by a combination of αHER2-Neo2A and αHER2-Neo2B (**b**). **c**, End-point NCI-H1975 tumor cell counts (left) and CAR-T cell counts (right) after each round of co-culture (40 hours). Cells were co-cultured four consecutive times. n = 2 independent cell co-cultures. Data are presented as mean values +/− s.d. **d**, Kinetic read of NCI-H1975 tumor cell index on each co-culture tumor challenge shown in (**c**, left).

**Fig. 5 |:**
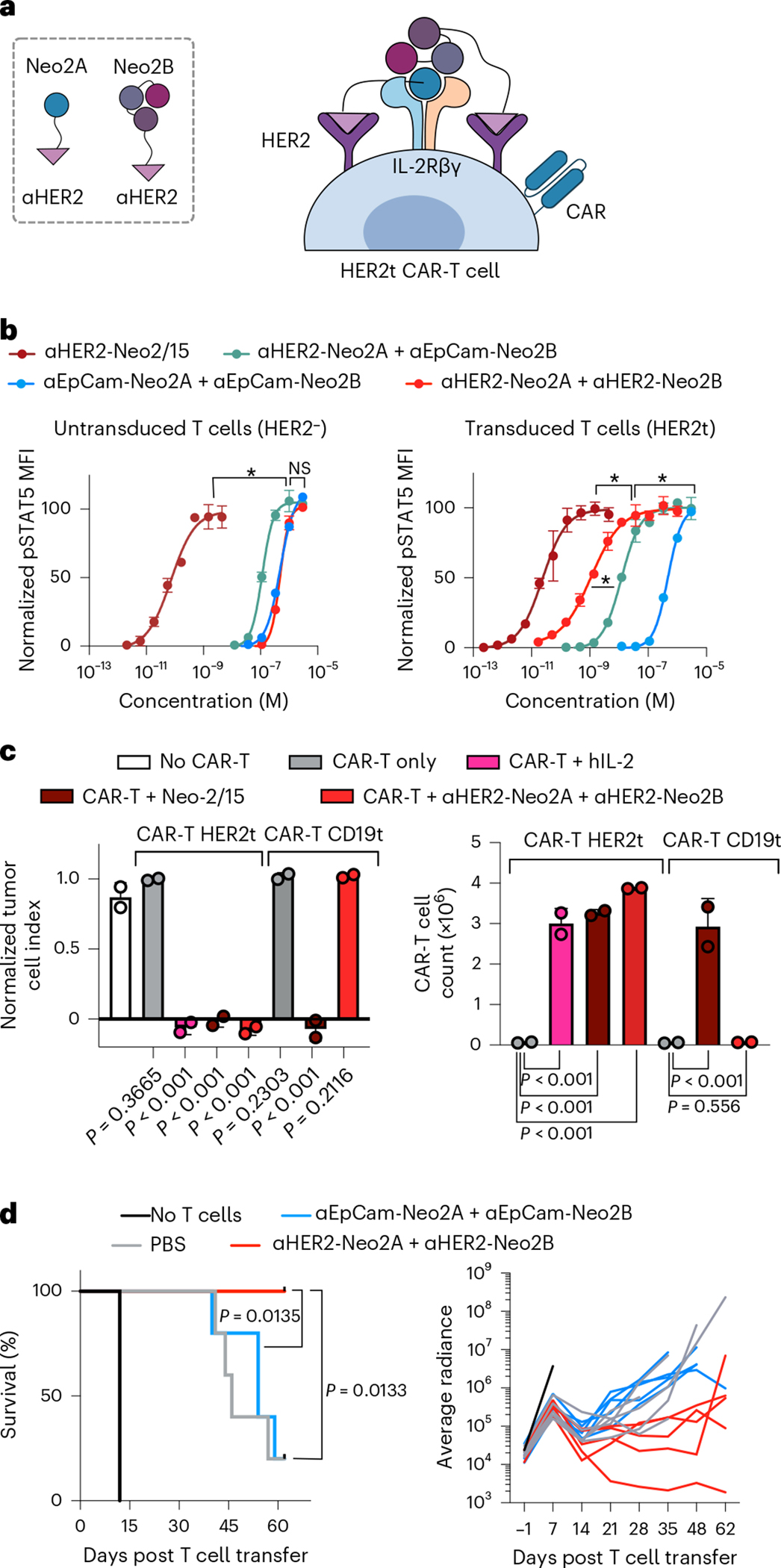
*Cis*-targeting split Neo-2/15 to CAR-T cells enhances CAR-T proliferation and tumor killing. **a**, *Cis*-activation mechanism of split Neo-2/15 targeted to HER2 expressed as a transduction marker on the surface of CAR-T cells. **b**, STAT5 phosphorylation of untransduced HER2^−^ primary CD8^+^ T cells (left) or transduced HER2^+^ CD8^+^ T cells expressing the ɑCD19 CAR (right) (*n* = 2 cell samples). An EpCam-binding DARPin (Ec1) was fused to split Neo-2/15 fragments as an untargeted control. Data presented as mean ± s.d. * Indicates different EC_50_ values with nonoverlapping 95% confidence interval ranges. **c**, In vitro coculture of CAR-T cells and NCI-H1975 (ROR1^+^) tumor cells in the presence of *cis*-targeted split Neo-2/15 costimulation. Anti-ROR1 CAR-T cells were engineered to express either HER2 (HER2t, on-target cells) or CD19 (CD19t, off-target control cells) as a transduction marker. CAR-Ts were cocultured with tumor cells for a total of four consecutive stimulations in the presence of the test items. End-point data showing tumor cell index (left) and CAR-T cell proliferation (right) after 35 h on the fourth stimulation of CAR-T cells with tumor cells. *n* = 2 independent cell cocultures. Statistical analysis performed with one-way ANOVA comparing each test group with control group (left: no CAR-T group; right: CAR-T-only groups). Data presented as mean ± s.d. **d**, Efficacy of ɑCD19 CAR-T cells and cotreatment with *cis*-targeted split Neo-2/15 fragments in a lymphoma xenograft model. Both HER2- and EpCam-targeted (negative control) split Neo2/15 fragments were dosed at 7.5 mg/kg (*n* = 5 mice per group). Data presented as mean ± s.d. **a**–**d**, Statistical analysis for survival curves was performed by Mantel− Cox log rank.

CAR-T therapies have achieved success in a subset of patients with B cell malignancies, but many patients do not respond to these treatments and additional challenges remain for their clinical application in solid tumors^41,42^. For instance, insufficient CAR-T cell expansion is correlated with treatment failure in hematological malignancies^43^, and poor CAR-T cell accumulation at the tumor site is believed to lessen effectiveness in the treatment of epithelial cancers^44,45^. We hypothesized that highly specific targeting of split Neo-2/15 to CAR-T cells would result in improved therapy through the selective expansion of these cells and reduced interaction with other immune cells that can lead to toxicity by immune overactivation. We engineered CAR-T cells to coexpress truncated HER2 as a transduction marker for use as a target for the split Neo-2/15 αHER2 fusion proteins described above (Fig. 5a). Split Neo-2/15 fragments fused to an EpCam-binding DARPin (Ec1)^46^ were used as untargeted controls. In vitro, ɑHER2 split Neo-2/15 molecules selectively activated IL-2 signaling leading to STAT5 phosphorylation in CAR-transduced HER2^+^ cells, whereas ɑHER2-fused intact Neo-2/15 activated either transduced (HER2^+^) or untransduced (HER2^−^) cells (Fig. 5b). To evaluate whether this selective activation also led to improved tumor-killing capacity, we cocultured αROR1 CAR-T cells expressing either surface HER2 (HER2t) or a control CD19 transduction marker (CD19t, negative control) with NCI-H1975 tumor cells, in the presence or absence of *cis*-targeted split Neo-2/15 proteins. HER2t CAR-T cells elicited potent tumor killing and proliferation in the presence, but not the absence, of *cis*-targeted split Neo-2/15 (Fig. 5c and Extended Data Fig. 10). In contrast, control CD19t CAR-T cells did not exhibit tumor killing in the presence of the split system, demonstrating that *cis*-targeting is required to sustain potent CAR-T activity.

To investigate the therapeutic potential of the *cis*-targeting approach we tested *cis*-targeted split Neo-2/15 in a lymphoma xenograft model (Raji). Cotreatment with αCD19 CAR-T cells and targeted split Neo2/15 showed improved tumor control and prolonged survival compared with control mice treated only with CAR-T and compared with the untargeted split Neo2/15 (Fig. 5d and Supplementary Fig. 5). Taken together, these *cis*-activation studies illustrate the potential of our targeted conditional activation approach for use in cancer immunotherapy.

## Discussion

We describe a conditionally active cytokine system designed to overcome residual off-target activity that is frequently observed with systemic cytokine and immunocytokine treatment^6,7^. To our knowledge, no split IL-2-like molecule with designed two-component conditional activity has previously been reported. The two components have no activity on their own, allowing for targeting and localization of cytokine activity to the desired site of action. The bipartite nature of our strategy allows for specific targeting of cells that express two surface receptors of interest while sparing cells that express only one receptor (that is, AND logic^30,47^). A further advantage of the split system is that, because multiple surface binding events are required for activation, it can distinguish tumor cells expressing high levels of target antigens from those expressing lower levels more effectively than intact targeted cytokines. Targeting the two split components to the tumor (to either one or two surface antigens) to elicit *trans*-activation resulted in an increased therapeutic index and led to complete remission in syngeneic melanoma tumor models. Targeting these components to specific immune cells to elicit *cis*-activation enhances antitumor immunotherapeutic effects in syngeneic melanoma and lymphoma xenograft models in mice.

For this study we selected human HER2, human EGFR, murine PD-L1 and murine CD8 as target antigens, but the split components can in principle be fused to any high-affinity-targeting domains that engage clinically validated targets highly expressed in the tumor microenvironment for *trans*-activation approaches^48,49^ or lymphocyte antigens for immune cell *cis*-activation^50,51^. Beyond cancer immunotherapy, the modularity of the split cytokine system could enable targeting immune cell subsets of interest, such as Tregs for treatment of autoimmune disorders^52,53^. Further development of this technology toward clinical applications will require optimization of the administration route, dosing regime and pharmacokinetics. As shown here, both components can either be mixed and dosed together or administered separately; likewise in a clinical setting, possibilities include both a single injection of both split parts with proper formulation or separate intravenous administration of the two split parts at two distinct sites (for example, both arms).

In conclusion, the development of split Neo-2/15 addresses the two current main engineering challenges in regard to IL-2 therapeutics (CD25 bias and undesired systemic activity). Our study demonstrates how the robust folding and high stability of de novo designed proteins can be exploited to create conditionally active signaling molecules that function robustly in preclinical models and reduce systemic toxicity. More generally, this approach opens the door to new applications in which a de novo designed protein can be leveraged to develop highly specific therapeutic proteins that are conditionally activated by target proteins of interest.

## Methods

### Synthetic gene construction

The designed protein sequences were codon optimized for *Escherichia coli* expression and ordered as synthetic genes in pET29b + *E. coli* expression vectors (Genscript). Each synthetic gene was inserted at the NdeI and XhoI sites of the vector, including an N-terminal hexahistidine tag followed by a TEV protease cleavage site and the addition of a stop codon at the C terminus. VHH fusion proteins were inserted in pCMV/R for mammalian cell expression cloned between NheI and AvrII sites and including an N-terminal signal peptide (MDSKGSSQKGSRLLLLLVVSNLLLPQGVLAGSDG) (Genscript). Amino acid sequences are listed in Supplementary Tables 1–3.

### Protein expression and purification in *E. coli*

All DARPin protein fusions H132’ and H32’4 were expressed in *E. coli* strain Lemo21(DE3) (NEB) and VHH fusion proteins in *E. coli* strain SHuffle T7 (NEB). Bacteria were transformed with a pET29b^+^ plasmid encoding the synthesized gene of interest. Cells were grown for 24 h in lysogeny broth medium supplemented with kanamycin. Cells were inoculated at a ratio of 1:50 ml in Studier TBM-5052 autoinduction medium supplemented with carbenicillin or kanamycin, grown at 37 °C for 2–4 h and then grown at 18 °C for an additional 18 h. Cells were harvested by centrifugation at 4,000*g* and 4 °C for 15 min and resuspended in 30 ml of lysis buffer (20 mM Tris-HCl pH 8.0, 300 mM NaCl, 30 mM imidazole, 1 mM PMSF, 0.02 mg ml^−1^ DNAse). Cell resuspensions were lysed by sonication for 2.5 min (5-s cycles). Lysates were clarified by centrifugation at 24,000*g* at 4 °C for 20 min and passed through 2 ml of Ni-NTA nickel resin (Qiagen, no. 30250) pre-equilibrated with wash buffer (20 mM Tris-HCl pH 8.0, 300 mM NaCl, 30 mM imidazole). The resin was washed twice with ten column volumes (CV) of wash buffer and then eluted with 3 CV of elution buffer (20 mM Tris-HCl pH 8.0, 300 mM NaCl, 300 mM imidazole). Eluted proteins were concentrated using Ultra-15 Centrifugal Filter Units (Amicon) and further purified with a Superdex 75 Increase 10/300 GL (GE Healthcare) size-exclusion column in Tris-buffered saline (25 mM Tris-HCl pH 8.0, 150 mM NaCl). Fractions containing monomeric protein were pooled and concentrated, concentration measured by absorbance at 280 nm (NanoDrop), snap-frozen in liquid nitrogen and stored at −80 °C. Proteins used in animal studies were further purified to remove endotoxin using NoEndo High Capacity Spin Columns (Protein Ark). Endotoxin levels were measured with an Endosafe LAL Cartridge, PTS201F 0.1 EU ml^−1^ sensitivity (Charles River) in an Endosafe nexgen-MCS (Charles River).

### Protein production in 293–6E cells

Nanobody (VHH) fusion proteins used in animal studies were expressed in 293–6E cells. Four liters of 293–6E cells were transfected with pCMV/R plasmids encoding the gene of interest using linear polyethylenimine. Cultures were harvested when average viability was ≤90% (4–6 days). Protein expression was determined in the culture supernatant, and total cell lysate was analyzed by reducing and nonreducing SDS–polyacrylamide gel electrophoresis (SDS–PAGE). His-tagged proteins were purified from clarified culture supernatant via Ni-NTA affinity chromatography. Proteins were then dialyzed overnight into PBS pH 7.4, concentrated using Ultra-15 Centrifugal Filter Units (Amicon) and analyzed by reducing and nonreducing SDS–PAGE and analytical size-exclusion chromatography. Finally, concentration was measured by absorbance at 280 nm (NanoDrop) and aliquots were snap-frozen in liquid nitrogen and stored at −80 °C. Expression and purification were performed by Proteos.

### Peptide synthesis

Peptides H1 (Neo2A), H13, H2’4 and H4 were synthesized to >85% purity by Genscript. For Biolayer interferometry assays measuring the affinity between Neo2A and Neo2B, the Neo2A peptide was biotinylated at the N terminus and a flexible linker added to avoid steric hindrance (Biotin-GGGSGGGSPKKKIQLHAEHALYDALMILNIVKTNS).

### Biolayer interferometry

Binding data were collected in an Octet RED96 (ForteBio) and processed using ForteBio Data Analysis Software v.9.0.0.10. Biotinylated human IL-2Rγ (Acro Biosystems, no. ILG-H85E8) was immobilized on streptavidin-coated biosensors (SA ForteBio) at 5 μg ml^−1^ in binding buffer HBS:EP^+^ (Cytiva) + Blotting Grade Blocker Non Fat Dry Milk (BioRad) until the signal reached 0.5 nm. After loading the IL-2Rγ receptor onto the biosensor, baseline measurement was performed by dipping the biosensors in binding buffer only (60 s), followed by monitoring of binding kinetics by dipping the biosensors in wells containing the target analyte protein (association step) and then dipping them back into baseline/buffer (dissociation). For the association step, analyte proteins were diluted from concentrated stocks into binding buffer to the indicated final concentration. Human IL-2Rβ (Acro Biosystems, no. CD2-H5221) was added in solution at the indicated concentration. The experiments using Biotin-Neo2A were performed following the same method, except that Biotin-Neo2A was loaded on the streptavadin sensors and human IL-2Rγ-Fc (Acro Biosystems, no. ILG-H5256) was also added in solution during association.

### Circular dichroism

Far-ultraviolet circular dichroism measurements were carried out with AVIV spectrometer model 420 in PBS (pH 7.4) in a cuvette of 1-mm path length at a protein concentration of ~0.20 mg ml^−1^ (unless otherwise mentioned in the text). Temperature melts were performed at 25–95 °C and monitored absorption signal at 222 nm (steps of 2 °C min^−1^, 30 s of equilibration by step). Wavelength scans (195–260 nm) were collected at 25 and 95 °C, and again at 25 °C after rapid refolding (~5 min).

### Cell lines and cell culture

YT-1 cells were provided by Yodoi^31^ and cultured in RPMI complete medium (Gibco): RPMI 1640 medium supplemented with 10% fetal bovine serum (FBS), 2 mM l-glutamine, 1% nonessential amino acids, 1 mM sodium pyruvate, 15 mM HEPES and 1% penicillin/streptomycin. WT K562 cells were purchased from American Type Culture Collection (ATCC), and engineered K562 cell lines expressing EGFR-iRFP, Her2-eGFP or both (provided by Lajoie^30^) were cultured in RPMI complete medium. WT B16F10 melanoma cells (purchased from ATCC), PD-L1-overexpressing B16 melanoma cells^35^ and Her2^+^/PD-L1Hi B16F10 melanoma cells were cultured in RPMI medium supplemented with 10% FBS, 2 mM l-glutamine, 1% penicillin/streptomycin, 1% nonessential amino acids, 1 mM sodium pyruvate (Gibco) and 0.1 mM β-mercaptoethanol. Cell lines SKOV3, JIMT-1 and OVCAR8 were purchased from ATCC and cultured in RPMI complete medium. NCI-H1975 cells were purchased from ATCC and cultured in RPMI + 5% FBS + 10 U ml^−1^ penicillin/streptomycin. Cells were trypsinized and split every 3 days to avoid confluency. Cell line 293–6E (human embryonic kidney) was provided by Proteos and cultured in F17 supplemented with 0.1% Pluronic F-68, 4 mM GlutaMAX and 25 μg ml^−1^ G418. All cells were maintained at 37 °C in a humidified incubator with 5% CO_2_. Murine IFN-ɣ was purchased from Peprotech and used at the indicated concentrations to induce PD-L1 expression on B16F10 cell lines. Cells used for in vivo experiments had been passaged for <2 months, were negative for known mouse pathogens and were implanted at >95% viability.

### YT-1 cell STAT5 phosphorylation studies

Approximately 2 × 10^5^ YT-1 cells were plated in each well of a 96-well plate and resuspended in RPMI complete medium containing serial dilutions of targeted or untargeted intact or split Neo-2/15 proteins. Cells were stimulated for 20 min at 37 °C and immediately fixed by the addition of formaldehyde to 1.5% and 10 min incubation at room temperature. After fixation, cells were permeabilized by resuspension in ice-cold 100% methanol for 30 min on ice. Fixed and permeabilized cells were washed twice with PBSA buffer (PBS pH 7.2 containing 0.1% bovine serum albumin) and then incubated with Alexa Fluor 647-conjugated anti-STAT5 pY694 antibody (BD Biosciences) diluted 1:50 in PBSA buffer for 2 h at room temperature. Cells were washed twice in PBSA buffer, and Alexa Fluor 647 was measured on a CytoFLEX flow cytometer (Beckman Coulter). Dose–response curves were fitted to a logistic model and half-maximal effective (EC_50_) concentrations calculated using GraphPad Prism data analysis software, after subtraction of mean fluorescence intensity (MFI) of unstimulated cells and normalization to maximum signal intensity. Experiments were conducted in triplicate and performed three times, with similar results. The gating strategy is shown in Supplementary Fig. 6.

### In vitro reconstitution of split Neo-2/15 on target cells

The four K562 cell lines (engineered K562 tumor cell lines transduced for expression of EGFR-iRFP, HER2-eGFP, both or neither) were mixed in equivalent ratios. Cell mixtures were washed with flow buffer (20 mM Tris pH 8.0, 150 mM NaCl, 1 mM MgCl_2_, 1 mM CaCl_2_ and 1% BSA) and aliquoted into V-bottom plates with 200,000 cells per well. Serially diluted split Neo-2/15 fusion proteins were made from concentrated stocks, added to cells in a 50-μl volume and incubated for 30 min. Subsequently, cells were washed with 150 μl of flow buffer and incubated with a mixture of biotinylated human IL-2Rγ (Acro Biosystems, no. ILG-H85E8), human IL-2R03B2 (Acro Biosystems, no. CD2-H5221) and a fluorescent streptavidin–phycoerythrin conjugate (SA-PE, Invitrogen) for 15 min. Data were acquired on a LSRII cytometer (BD Biosciences). The gating strategy is shown in Supplementary Fig. 7. The same protocol was used to measure targeted split Neo-2/15 activity on cell lines SKOV3, JIMT-1, OVCAR8 and B16F10 acquired on a fluorescent activated cell sorter (FACS) Celesta cytometer (BD Biosciences).

### YT-1:K562 cell *trans*-activation assays

Approximately 2 × 10^5^ YT-1 cells at the indicated ratio (K562:YT-1 20:1, 6:1 or 2:1) (either untransduced HER2^−^/EGFR^−^ K562 cells or transduced double-positive HER2^+^/EGFR^+^ K562 cells) were used in each well for *trans*-activation studies. K562 cells were plated in each well of a 96-well plate and resuspended in RPMI complete medium containing serial dilutions of split Neo-2/15 proteins. Cells were incubated for 30 min at 37 °C and then washed once with PBSA buffer. YT-1 cells were added to each well and cocultured with K562 cells for 30 min at 37 °C. Cells were stained with BV421-conjugated anti-CD132 antibody (BD Biosciences, no. 566222; Clone AG184, 1:50 dilution) for 30 min at 4 °C and washed once with PBSA buffer. After washing, these were immediately fixed by the addition of formaldehyde to 1.5% and 10-min incubation at room temperature. After fixation, cells were permeabilized by resuspension in ice-cold 100% methanol for 30 min on ice. Fixed and permeabilized cells were washed twice with PBSA buffer then incubated with Alexa Fluor 647-conjugated anti-STAT5 pY694 antibody (BD Biosciences) diluted 1:50 in PBSA buffer for 2 h at room temperature. Alexa Fluor 647 fluorescence on YT-1 cells (gated based on CD132 expression) was measured on a CytoFLEX flow cytometer (Beckman Coulter). Dose–response curves were fitted to a logistic model and EC_50_ values calculated using GraphPad Prism data analysis software after subtraction of MFI of unstimulated cells and normalization to maximum signal intensity. Experiments were conducted in duplicate and performed twice, with consistent results. The gating strategy is shown in Supplementary Fig. 8.

### In vitro T cell:B16 *trans*-activation experiments

B16 cells overexpressing PD-L1 were treated overnight with 20 ng ml^−1^ murine IFN-γ (Preprotech), washed, coated with 1.0 μM desired protein (that is, OVA, Neo-2/15, Ctrl-Neo2A and so on) for 10 min, washed and resuspended repeatedly and replated in a U-bottom plate. Mouse trp1-specific CD8 T cells (JAX Stock, no. 030958) were isolated via a negative isolation kit (Stemcell, no. 19853) and plated under the listed conditions either alone or in coculture with B16 cells at a 1:1 ratio for 1 day. To confirm *trans*-activation, 1.0 μM αPD-L1 or Ctrl VHH was added at the start of coculture. T cells were then harvested 24–36 h later and analyzed by flow cytometry for expression of activation markers CD25 (Biolegend, no. 102017) and CD69 (Biolegend, no. 104508). T cells were gated separately from B16 cells via FSC/SSC gating and CD8 staining (Biolegend, no. 100728). All antibodies were used at 1:100 dilution.

### Engineering of B16F10 melanoma cells overexpressing mouse PD-L1 and human HER2

B16F10 PD-L1hi cells were transduced with retrovirus to express human HER2 (extracellular domain IV and transmembrane domain). HER2^+^ cells were identified by staining with anti-HER2 antibody (24D2, Biolegend, no. 324405, 1:200 dilution) and FACS sorted twice to achieve a pure population. The gating strategy is shown in Supplementary Fig. 4.

### Safety study in murine models

As shown in Fig. 3a and Supplementary Fig. 2: Immunocompetent female 6–8-week-old C57BL/6J mice (*n* = 5 per group) were treated daily with the targeted Neo-2/15 and targeted split Neo-2/15 molecules at equivalent doses (2.6 nmol, equivalent to 30 μg per mouse of Neo2/15). Weight change and survival were monitored to evaluate toxicity of the dosed molecules. Mice were euthanized if they lost 10% of body weight. Spleen and lungs were harvested following euthanasia. Organs from mice in cohorts Neo-2/15, Ctrl-Neo2/15 and αPD-L1-Neo2/15 were obtained and analyzed on different days, when the euthanasia criteria were met. Organs from mice in the PBS and split Neo-2/15 cohorts that did not lose weight and did not meet euthanasia criteria were harvested on day 60 at the conclusion of the study. Spleens and lungs were weighed to assess the potential toxicity of treatment. Spleens were subjected to immunophenotyping by flow cytometry to quantify expansion of CD8^+^ T cells using the following antibodies: Alexa Fluor 488 anti-CD25 (clone PC61, Biolegend, no. 102018); PE anti-CD69 (H1.2F3, Biolegend, no. 104508); Pacific Blue anti-CD4 (GK1.5, Biolegend, no. 100428); Brilliant Violet 711 anti-CD45 (30-F11, Biolegend, no. 103147); and Brilliant Violet 785 anti-CD8a (53–6.7, Biolegend, no. 100749). All antibodies were used at 1:100 dilution. All animal protocols were approved by the Dana-Farber Cancer Institute Committee on Animal Care (protocol nos. 14–019 and 14–037) and are in compliance with the NIH/NCI ethical guidelines for tumor-bearing animals.

### Syngeneic murine melanoma model experiments

All animal protocols were approved by the Dana-Farber Cancer Institute Committee on Animal Care (protocol nos. 14–019 and 14–037) and are in compliance with the NIH/NCI ethical guidelines for tumor-bearing animals. At day 0, 6–8-week-old C57BL/6 J mice (JAX Stock, no. 000664) were inoculated with 80–90% confluent WT or engineered B16F10 cells (500,000 cells per mouse). Starting at day 5, mice were treated daily with the listed test items. Neo2A and Neo2B fusion proteins were injected individually following one of two regimens as indicated per experiment. Mice were monitored for survival, weight change and symptoms of toxicity including pallor, notable weight loss and fatigue. Mice were euthanized if they lost 10% of body weight or their tumors ulcerated or reached 2,000 mm^3^ in volume, which is the maximal permitted tumor size for these studies.

Experiments shown in Fig. 3b and Supplementary Fig. 3: Five mice per group were included in these two studies. For the study shown in Fig. 3b, all groups were cotreated biweekly with TA99 starting on day 3 (150 μg per mouse). Mice in the study shown in Supplementary Fig. 3 were not cotreated with TA99. The two studies were carried out in parallel. Starting on day 5 after tumor cell inoculation, mice were administered daily with therapeutic doses of αPD-L1-Neo2/15 and Ctrl-Neo2/15 at 430 nmol (12 μg per mouse) (intraperitoneally), or targeted split Neo-2/15 fragments were administered at 8 nmol (200 μg per mouse) (Neo2A fusions given intraperitoneally and Neo2B fusions given subcutaneously in the opposite flank to the tumor). Mice in the αPD-L1-Neo2/15 treatment cohort showed symptoms of toxicity (pallor, notable weight loss, fatigue) at day 12 and so their treatment schedule was modified. They then received treatment daily from days 5–12, no treatment on days 13 and 14 and then treatment every other day starting at day 15. Peripheral blood was collected at day 15 of treatment and analyzed by flow cytometry using the following antibodies: Alexa Fluor 488 anti-CD25 (clone PC61, Biolegend, no. 102018); PE anti-CD69 (H1.2F3, Biolegend, no. 104508); Pacific Blue anti-CD4 (GK1.5, Biolegend, no. 100428); Brilliant Violet 711 anti-CD45 (30-F11, Biolegend, no. 103147); and Brilliant Violet 785 anti-CD8a (53–6.7, Biolegend, no. 100749). All antibodies were used at 1:100 dilution.

Experiments shown in Fig. 3c,d: Five mice were used in the PBS and TA99 groups, ten mice in all other groups. Starting on day 5 after tumor cell inoculation, mice were administered daily with each test item at therapeutic doses (Neo-2/15 at 2.6 nmol (30 μg per mouse) and split Neo-2/15 fusions at 8 nmol (~200 μg per mouse)). The indicated groups were cotreated with Ta99 mAb administered biweekly starting on day 3 (150 μg per mouse).

Rechallenge experiments shown in Fig. 3e: Five surviving, tumor-free mice from the αPD-L1 split Neo-2/15 + TA99-treated cohorts in several experiments (including the studies shown in Fig. 3b,c) were pooled into one group. The mice had cleared their tumors 2–5 months before the start of the experiment, as shown in Fig. 3e. Five age-matched B16 naïve C57BL/6J mice were designated as controls. On day 0, mice were inoculated with B16 F10 cells overexpressing PD-L1 (1,000,000 cells per mouse, twice the amount of a usual primary challenge). On day 5, mice were bled and peripheral blood analyzed by flow cytometry for the presence of tumor-specific Trp1^+^ CD8 T cells via anti-Trp1-MHCI tetramer staining as previously described^34^.

Experiments shown in Fig. 3f and Extended Data Fig. 6c: At day 0, mice were inoculated in the flank with 80–90% confluent B16F10 cells overexpressing both PD-L1 and HER2 (500,000 cells per mouse). Twelve mice were included in the PBS control group, five in the Neo-2/15-treated group and seven each in groups ɑPD-L1-Neo2A + ɑPD-L1-Neo2B and ɑHER2-Neo2A + ɑPD-L1-Neo2B. Starting on day 5 after tumor cell inoculation, mice were administered daily with each test item at therapeutic doses (Neo-2/15 at 2.6 nmol (30 μg per mouse) and split Neo-2/15 fusions at 8 nmol (~200 μg per mouse)). Neo2A fusions were administered subcutaneously interscapularly while PBS, Neo-2/15 and Neo2B were injected intraperitoneally. The mice in these treatment cohorts did not receive TA99 mAb. Mice in the Neo2/15 treatment cohort showed symptoms of toxicity (pallor, notable weight loss, fatigue) after 1 month of daily dosing, and thus surviving mice were euthanized when their body condition score was less than or equal to 2, in accordance with IACUC Standard Procedure.

Experiments shown in Extended Data Fig. 7d: Five mice per group were included in this study. Starting on day 5 after tumor cell inoculation, mice were administered daily with therapeutic doses of mIL-2 (13 μg per mouse) (Preprotech, no. 212–12), Neo-2/15 (10 and 30 μg per mouse) and αPD-L1-Neo2A + αPD-L1-Neo2B at 8 nmol (200 μg per mouse). Neo2B fusions were given intraperitoneally and Neo2A fusions subcutaneously (in the scruff of the neck). All groups were cotreated with TA99 αTrp-1 melanoma-specific antibody, administered biweekly starting on day 3 (150 μg per mouse). Three early ulcerated tumors (<500 mm^3^) were removed from this analysis. Experiments shown in Fig. 4c: Starting on day 5 after tumor cell inoculation, mice were administered daily with: PBS (five mice), Ctrl-Neo-2/15 (500 pmol per mouse, 5 mice), Ctrl-Neo2A + Ctrl-Neo2B (500 pmol per mouse, 5 mice) and αCD8-Neo2A + αCD8-Neo2B (500 pmol per mouse, 10 mice). Neo2B fusions were given intraperitoneally and Neo2A fusions subcutaneously (in the scruff of the neck). All groups were cotreated with TA99 αTrp-1 melanoma-specific antibody, administered biweekly starting on day 3 (150 03BCg per mouse).

### Quantibrite surface receptor quantification

The antibody-binding capacity of molecules EGFR (AY13, Biolegend, no. 352903), EpCAM (9C4, Biolegend, no. 324205), HER2 (24D2, Biolegend, no. 324405) and murine PD-L1 (MIH7, Biolegend, no. 155404) on the surface of K562, B16F10, SKOV3, JIMT-1 and OVCAR8 cells was determined using Quantibrite beads (BD Biosciences) according to the manufacturer’s protocols. Flow cytometry data were analyzed using FlowJo v.10. All antibodies were used at 1:200 dilution. The gating strategy is shown in Supplementary Fig. 9.

### In vitro CD8 *cis*-targeting experiments with splenocytes

Splenocytes were harvested from a WT C57BL/6 J mouse and plated together with split Neo-2/15 fusion test items at the indicated concentrations (negative-control, single-split construct wells were plated at 50 nM) in a 96-well plate in RPMI complete medium. After 4 days, cells were harvested and stained for flow cytometry. The antibodies used for staining were: APC anti-CD45 (clone 30-F11, Biolegend, no. 103111); Zombie NIRTM Fixable Viability Kit (Biolegend, no. 423105); Pacific Blue anti-B220 (RA3–6B2, Biolegend, no. 103227); and Brilliant Violet 421 anti-CD4 (GK1.5, Biolegend, no. 100443). All antibodies were used at 1:100 dilution.

### In vivo T cell *cis*-targeting in Foxp3-GFP mice

Split Neo-2/15 fusion proteins (or intact Neo2 constructs) were administered daily to nontumor-bearing Foxp3-GFP mice (JAX Stock, no. 006772) for 5 days. Neo2A fusions were injected subcutaneously in the scruff of the neck while Neo2B fusions, PBS and intact Neo-2/15 constructs were injected intraperitoneally. Intact Neo-2/15 fusions were dosed at 500 pmol (12 μg per mouse per day). Split Neo-2/15 fusions were dosed at 500 pmol (10 μg per mouse per day) and 250 pmol (4 μg per mouse per day). On day 6 mice were euthanized, spleen and both inguinal lymph nodes collected and homogenized into single-cell suspensions (and spleen resuspended briefly in ACK lysis buffer to lyse red blood cells) and cell populations investigated by flow cytometry using the following antibodies: APC anti-CD45 (clone 30-F11, Biolegend, no. 103111); Brilliant Violet 421 anti-CD4 (GK1.5, Biolegend, no. 100443); and PE anti-CD8a (53–6.7, Biolegend, no. 100707). All antibodies were used at 1:100 dilution.

### CAR-T cell in vitro STAT5 phosphorylation assay

Primary human CD8 T cells were obtained from healthy donors, with written informed consent for research protocols approved by the Institutional Review Board of the Fred Hutchinson Cancer Research Center (FHCRC). Peripheral blood mononuclear cells were isolated using lymphocyte separation medium (Corning). T cells were isolated using EasySep CD8 negative isolation kits (Stemcell Technologies). CAR-T cells were generated by stimulation of thawed CD8 with anti-CD3/CD28 Dynabeads (Gibco) at a ratio of three beads to one T cell, in medium supplemented with 50 IU ml^−1^ (3.1 ng ml^−1^) IL-2. The following day, T cells were transduced with lentivirus encoding anti-CD19 CAR by spinoculation for 90 mins at 800*g* or left untransduced. Beads were removed 5 days post stimulation. CAR^+^ cells were FACS sorted by HER2 transduction marker on day 8. On day 10, CAR-T cells or untransduced T cells were cultured at the indicated concentrations of Neo2 variants in 96-well plates for 12 min. T cells were then methanol fixed, permeabilized and stained for pSTAT5 for flow cytometry using pSTAT5-APC antibody (A17016B.Rec, Biolegend, no. 936902) at 1:200 dilution. The gating strategy is shown in Supplementary Fig. 10.

### Repeated tumor-killing assay by CAR-T cells

NCI-H1975 cells were seeded at 1.1 × 10^5^ per well in octuplet for each condition in 96-well E-plates (Agilent) and incubated overnight in the xCelligence Esight instrument (Agilent). Tumor cell growth was monitored in real time using electric impedance measurement. The following day, medium was decanted from the E-plate and 2.2 × 10^5^ anti-ROR1 CAR-T cells with either a HER2 or CD19 transduction marker were plated in medium or medium supplemented with 30 nM ɑHER2-Neo2A + ɑHER2-Neo2B, 30 nM ɑHER2-Neo-2/15 or 50 IU of hIL-2. Anti-ROR1 CAR-T cells (Clone R12), with IgG4 hinge CD28 transmembrane domain and 41BB CD3z costimulatory domains, were generated as previously described^54^. Forty-eight hours post CAR-T cell addition, CAR-T cells were removed from the E-plate by vigorous pipetting and transferred to an Eppendorf tube. Cells were counted, pelleted by centrifugation and resuspended in medium or medium supplemented with 30 nM ɑHER2-Neo2A + ɑHER2-Neo2B, 30 nM HER2-Neo-2/15 or 50 IU of hIL-2. CAR-T cells were then transferred to a fresh E-plate seeded with NCI-H1975 tumor cells. Replating was performed for a total of four times.

### In vivo lymphoma xenograft treatment with CAR-T cell study

Six-to-eight-week-old female NSG mice were obtained from the Jackson Laboratory, with five mice housed per cage. The temperature of the housing room was 68–75 °F and humidity 40–60%. Mice were provided with cellulose pellet bedding (Performance BioFresh), irradiated diamond twists as enrichment and nesting material (Envigo) and irradiated laboratory diet no. 5058. Water bottles were filled with acidified water. Raji tumor cells (0.5 × 10^6^) transduced with a lentiviral vector encoding the (ffLuc)-eGFP fusion gene were injected into the tail veins of NSG mice. CAR-T cells were produced as described above and expanded as previously described^55^ post FACS sorting. Seven days after tumor injection, lentiviral-transduced anti-CD19 HER2^+^ CAR-T cells (0.8 × 10^6^) were infused intravenously into mice. Either ɑHER2-Neo2A + ɑHER2-Neo2B or ɑEpCam-Neo2A + ɑEpCam-Neo2B, at 7.5 mg kg^−1^, was injected into the peritoneum on days 1–3, 6–10 and 13–15 post T cell injection. The FHCRC Institutional Animal Care and Use Committee approved all mouse experiments.

### Statistical analysis

Comparison of fitted curves in cellular phospho-STAT5 signaling assays was performed by measuring differences in EC_50_ values, which were considered statistically significant if their 95% confidence intervals did not overlap. For all bar plots, error bars represent ±s.d. and individual data points are shown (as dots) for experiments where *n* < 5. Comparisons of weight loss and tumor growth in tumor-bearing mice were performed using unpaired two-tailed Student’s *t*-test. Comparisons of the survival of tumor-bearing mice were performed using the log-rank Mantel–Cox test (95% confidence interval). Unless otherwise noted, the remaining results were analyzed by one-way ANOVA; if significant (95% confidence interval), post hoc *t*-tests were performed comparing groups and *P* values adjusted for multiple comparisons are reported. Statistical tests were performed with GraphPad Prism 9.0 data analysis software.

### Software

Protein visualization and protein structure images were generated using PyMOL 2.0. All flow cytometry data were analyzed using FlowJo v.9 and v.10. Statistical tests were performed with GraphPad Prism 8.0 and 9.0 data analysis software.

### Reporting summary

Further information on research design is available in the Nature Research Reporting Summary linked to this article.

## Extended Data

## Supplementary Material

Supplementary Info

## Data Availability

The original experimental data that support the findings of this work are available from the corresponding authors upon request. Plasmids encoding the proteins described in this article are available from the corresponding authors upon request. The original Neo-2/15 crystallographic structure can be accessed at the Protein Data Bank database, accession no. 6dg5.
