# Supplementary material for: A split, conditionally active mimetic of IL-2 reduces the toxicity of systemic cytokine therapy
Source: Nat Biotechnol. Author manuscript; Available in PMC 2023 Oct 1. (PMC10110466; doi:10.1038/s41587-022-01510-z)
Supplement: Supplementary Info [file NIHMS1859807-supplement-Supplementary_Info.pdf]

nature biotechnology

Article https://doi.org/10.1038/s41587-022-01510-z

A split, conditionally active mimetic of IL-2 reduces the toxicity of systemic cytokine therapy

In the format provided by the authors and unedited

Supplementary Information

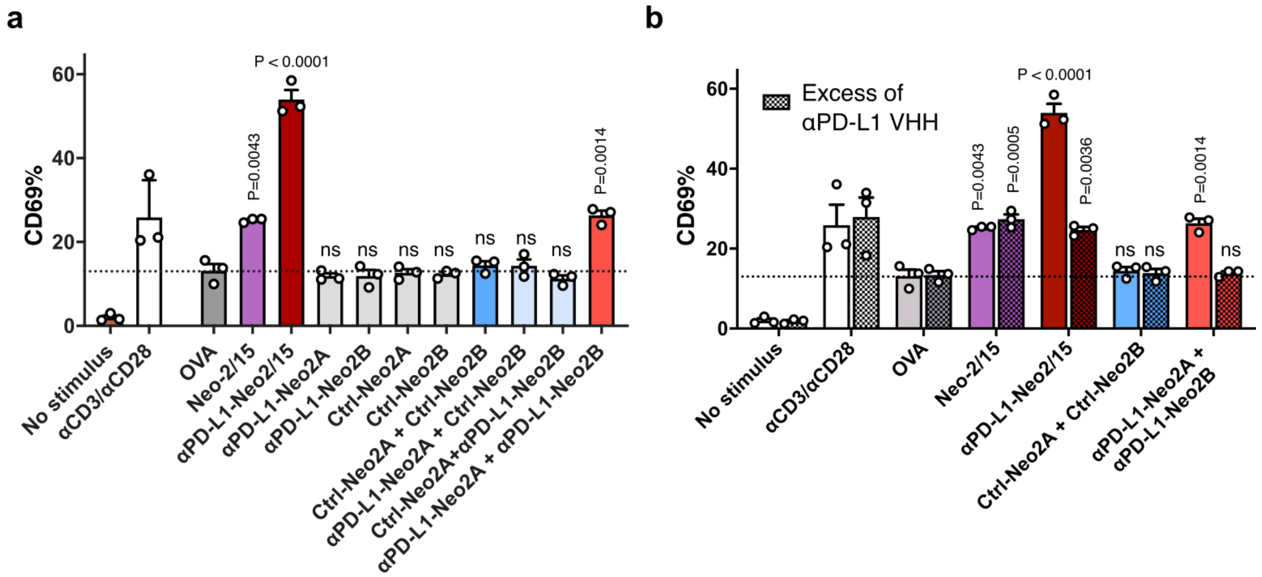

Supplementary Figure 1. CD69 upregulation data from CD8+ T cell trans-activation study shown in Fig. 2e. a, PD-L1-overexpressing B16 melanoma cells and αTrp-1 CD8+ T cells were co-cultured in the presence of the listed activating proteins at 1.0 μM concentrations. CD69 expression was measured to assess T cell activation. αCD3 and αCD28 antibodies were used as positive control for T cell activation. OVA peptide was used to quantify basal CD69 expression levels in co-culture conditions. All samples were incubated with an αCD28 antibody to provide a co-stimulatory signal. **b**, Addition of excess soluble αPD-L1 nanobody (VHH) to competitively inhibit binding of targeted split fusion proteins to the B16 cell surface reduced T cell activation, confirming trans-activation of immune cells from the surface of tumor cells. The experiments were performed in triplicate three times with similar results. One-way ANOVA comparisons against the OVA control group were performed to evaluate statistical significance. ns indicates no statistical significance. All data are presented as mean values +/- SD.

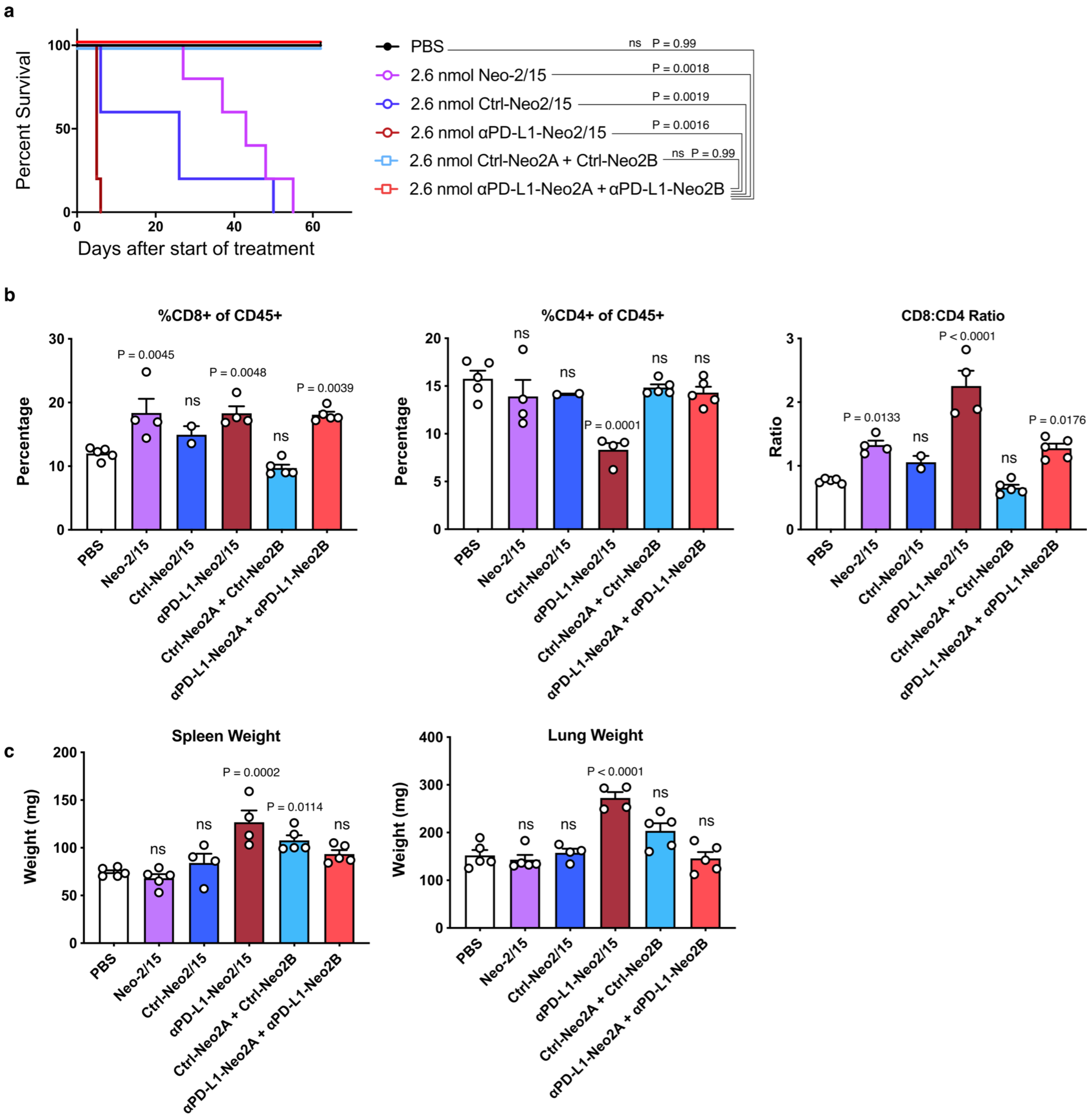

Supplementary Figure 2. Supplementary data for *in vivo* safety study presented in Figure 3a. Immunocompetent C57BL/6J mice (n=5/group) were treated with targeted Neo-2/15 and targeted Split Neo-2/15. Mice were treated daily with equivalent doses of the indicated proteins (2.6 nmol). Spleen and lungs were harvested when the euthanasia criteria were met. Organs from the mice in the PBS and split cohorts were obtained on day 60, at the conclusion of the study. **a**, Survival curve. Statistical analysis was performed by Mantel−Cox log rank. **b**, Spleens were subjected to immunophenotyping by flow cytometry. **c**, The spleens and lungs were weighed to assess potential toxicity of treatment. Wet lung masses are reported. ns = non significant, unpaired two-tailed Student’s t-test against the PBS group. Data in panels b-c are presented as mean values +/- SD.

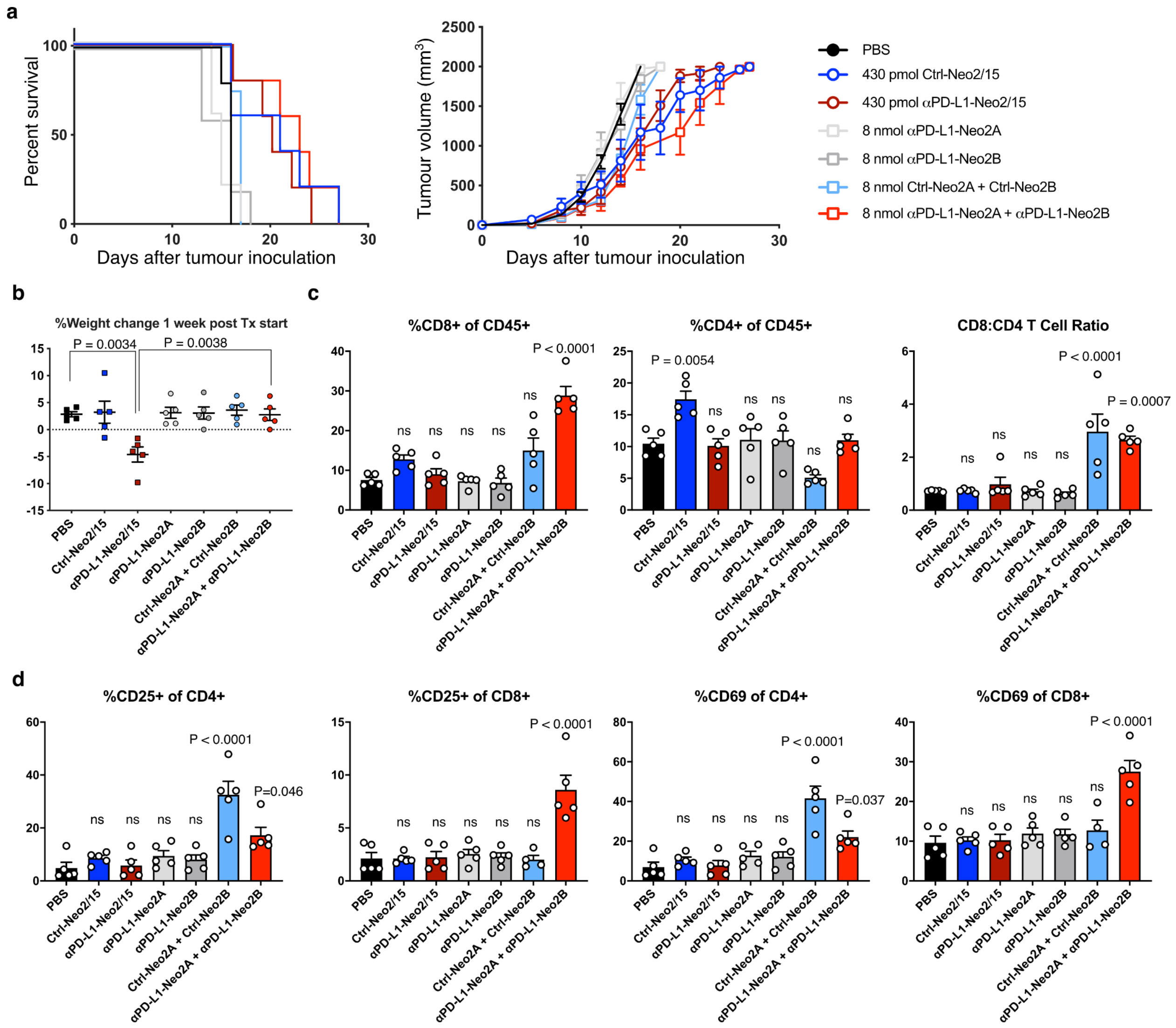

Supplementary figure 3. Supplementary data and peripheral blood analysis for the efficacy study presented in Figure 3b. C57BL/6J mice (n=5/group) bearing PD-L1-overexpressing B16 melanoma tumors in the flank were treated with the Neo-2/15 and Split Neo-2/15 fusion proteins as indicated. **a**, This efficacy experiment was carried out identically (and in parallel) to the efficacy experiment shown in Figure 3b. Data shown here were collected from mice that were not co-treated with TA99. Statistical analysis was performed by Mantel−Cox log rank. Right panel, data are presented as mean values +/- SEM. **b**, Weight change after one week of treatment for mice in the efficacy study shown in Figure 3b. **c**, Peripheral blood cell analysis of mice from the study shown in Figure 3b. Peripheral blood was collected at day 15 of treatment and analyzed by flow cytometry. Relative expansion of CD8+ T cells was measured to quantify *in vivo* activity of the Neo-2/15 and Split Neo-2/15 constructs. One-way ANOVA was performed to evaluate statistical significance. **d,** Expression of surface CD25 and CD69 on cells analyzed in (**c**) was measured to evaluate Split Neo-2/15-mediated T cell activation. One-way ANOVA was performed to evaluate statistical significance. b-d, Data are presented as mean values +/- SEM. ns indicates no statistical significance. Reported P-values compare each group to PBS group.

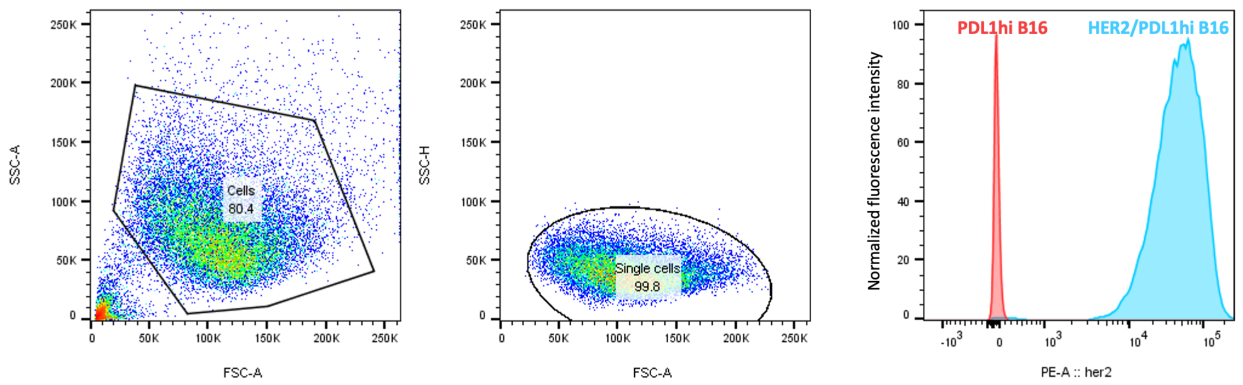

Supplementary Figure 4. Flow cytometry gating strategy for B16F10 cell lines overexpressing mouse PD-L1 and the full extracellular domain of human HER2.

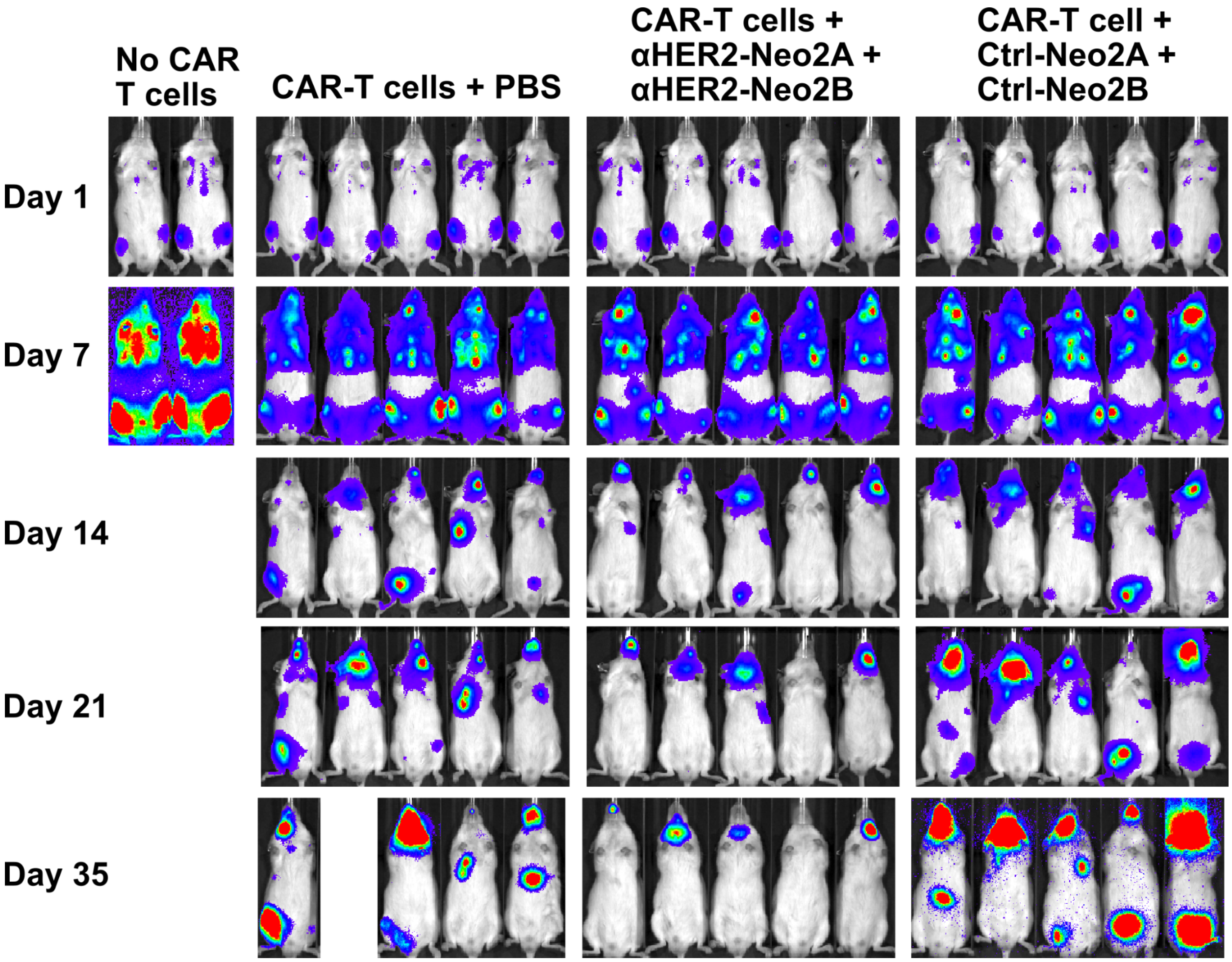

Supplementary figure 5. Tumor growth following co-therapy with anti-CD19 CAR-T cells and targeted Split Neo-2/15 in a lymphoma model shown in Figure 5d. NSG mice inoculated with $0.5x10^6$ RAJI tumor cells were treated with $0.8x10^6$ anti-CD19 CAR-T cells 7 days post-tumor inoculation. αHER2-Neo2A + αHER2-Neo2B or control αEpCam-Neo2A + αEpCam-Neo2B were injected intraperitoneally at 7.5mg/kg daily from day 1 to 3, day 6 to 10 and day 13 to 15 after CAR-T cell injection. RAJI tumor cells were transduced with (ffLuc)-eGFP to assess tumor growth via bioluminescence imaging. The experiment was performed once.

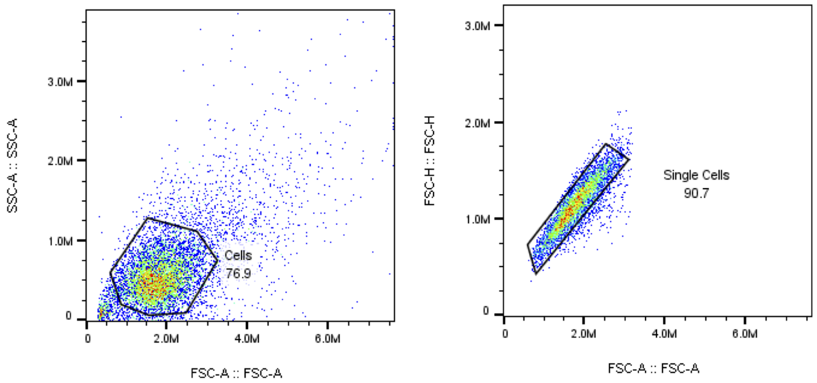

Supplementary Figure 6. Flow cytometry gating strategy for *in vitro* YT-1 cell STAT5 phosphorylation experiments.

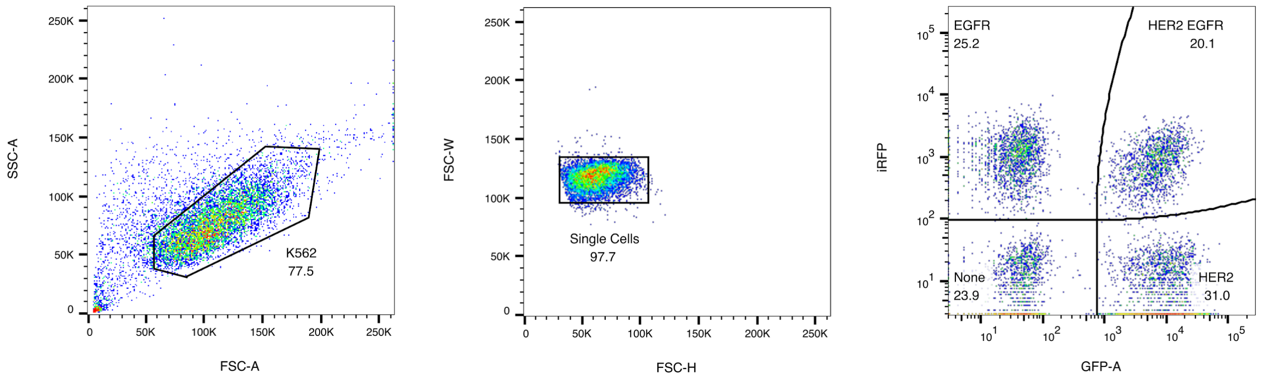

Supplementary Figure 7. Flow cytometry gating strategy for *in vitro* K562 cell targeting experiments. Four distinct K562 cell lines were mixed before the assay. The K562 cell lines were engineered to express EGFR-iRFP, HER2-eGFP, both, or neither.

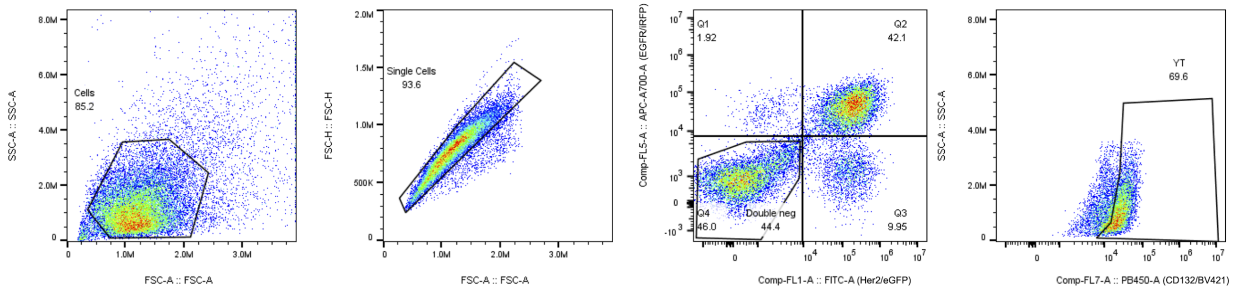

Supplementary Figure 8. Flow cytometry gating strategy for YT-1:K562 cell trans-activation assays. Two distinct K562 cell lines (double positive and double negative) were mixed with YT-1 cells before the assay. The K562 cell lines were engineered to express both EGFR-iRFP and HER2-eGFP or neither. K562 cells were gated using the iRFP and GFP channels. YT-1 cells lines were gated using a BV421-conjugated CD132 antibody.

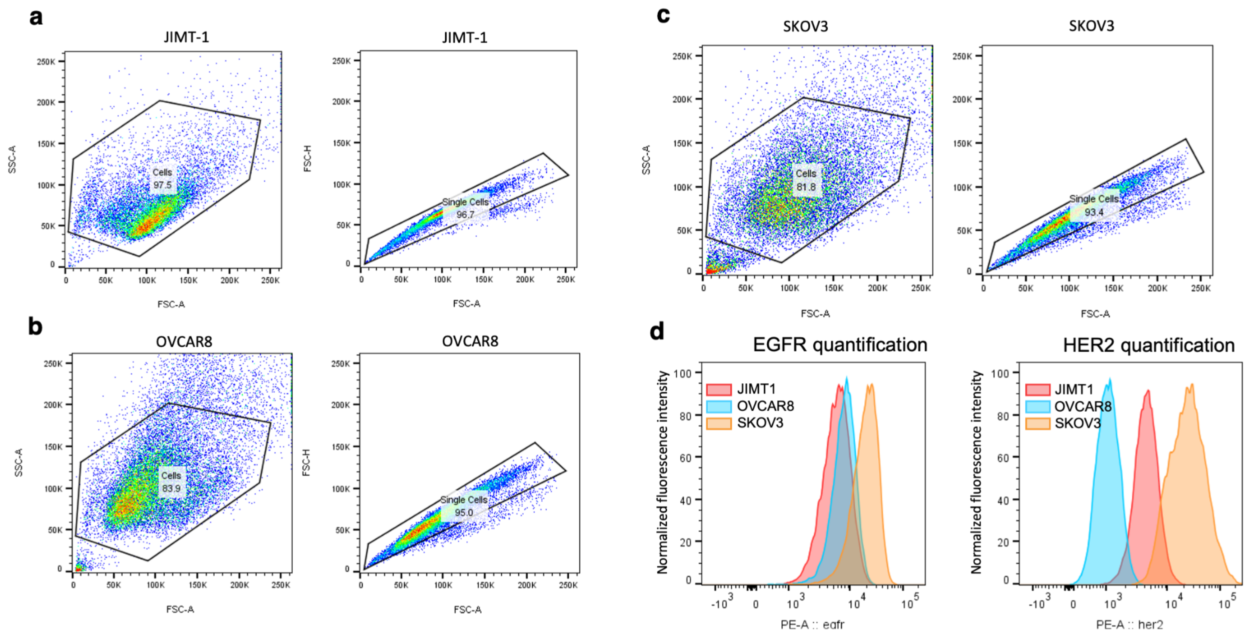

Supplementary Figure 9. Flow cytometry gating strategy for HER2 and EGFR surface receptor quantification on WT human cell lines. Gating strategy for JIMT-1 (a), OVCAR8 (b) and SKOV3 (c). d, Characterization of EGFR (left) and HER2 (right) surface receptor levels on each cell line.

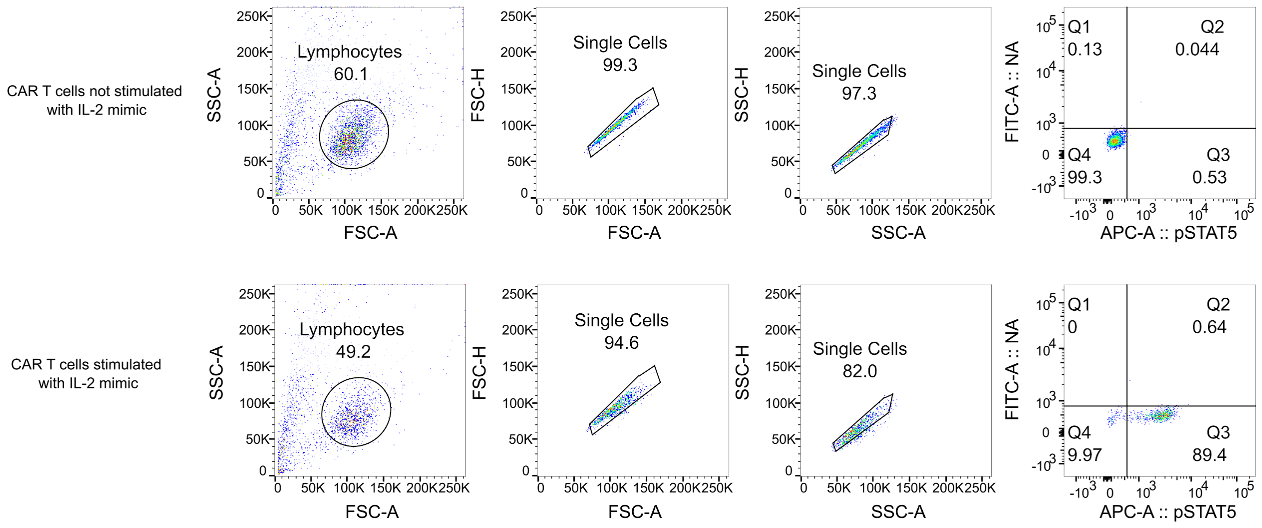

Supplementary Figure 10. Flow cytometry gating strategy for i*n vitro* CAR-T cells STAT5 phosphorylation experiments.

Supplementary Table 1. Split Neoleukin-2/15 variants sequences

Split Neoleukin-2/15 variants	Sequence
H1	PKKKIQLHAEHALYDALMILNIVKTNS
H3-H2'-H4	TNSPPAEEKLEDYAFNFELILEEIARLFESGDQKDEAEKAKRMKEWMKRIKTTASEDEQEEMANAIITILQSWIFS
H1-H3-H2'	PKKKIQLHAEHALYDALMILNIVKTNSPPAEEKLEDYAFNFELILEEIARLFESGDQKDEAEKAKRMKEWMKRIKTTAS
H4	TTASEDEQEEMANAIITILQSWIFS
H1-H3	PKKKIQLHAEHALYDALMILNIVKTNSPPAEEKLEDYAFNFELILEEIARLFESGD
H2'-H4	DQKDEAEKAKRMKEWMKRIKTTASEDEQEEMANAIITILQSWIFS

Supplementary Table 2. Targeting domains amino acid sequences

```
>αEGFR_DARPin_E01
DLGKKLLEAARAGQDDEVRILMANGADVNADDTWGWTPLHLAAYQGHLEIVEVLLKNGADVNAY
DYIGWTPLHLAADGHLEIVEVLLKNGADVNASDYIGDTPLHLAAHNGHLEIVEVLLKHGADVNA
QDKFGKTAFDISIDNGNEDLAEILQKLN

>αHER2_DARPin_G3
DLGKKLLEAARAGQDDEVRILMANGADVNAKDEYGLTPLYLATAHGHLEIVEVLLKNGADVNAV
DAIGFTPLHLAAFIGHLEIAEVLLKHGADVNAQDKFGKTAFDISIGNGNEDLAEILQKLN

>αPD-L1VHH_B3
QVQLVETGGGLVQPGGSLRLSCTASGFTFSMHAMTWYRQAPGKQRELVAVITSHGDRANYTDSV
RGRFTISRDNTKNMVYLQMNSLKPEDTAVYYCNVPRYDSWGQGTQVTVSSGG

>CtrlVHH_1B7
QVQLVETGGGLVQPGESLRLSCVASGFTLDHSAVGWFRQVPGKEREKLLCINANGVSLDYADSI
KGRFTISRDNAKNTVYLQMNDLKPEDTATYSCAATREFCSAYVFLYEHWGQGTQVTVSS

>αEPcam_DARPin_Ec1
DLGKKLLEAARAGQDDEVRILVANGADVNAYFGTTPLHLAAAHGRLEIVEVLLKNGADVNAQDV
WGITPLHLAAYNGHLEIVEVLLKYGADVNAHDTRGWTPLHLAAINGHLEIVEVLLKNVADVNAQ
DRSGKTPFDLAIDNGNEDIAEVLQKAAKLN
```

Supplementary Table 3. Fusion proteins amino acid sequences

>Split_Neo215_H132’
(MGSHHHHHHGSGSENLYFQGSG)PKKKIQLHAEHALYDALMILNIVKTNSPPAEEKLEDYAFN
FELILEEIARLFESGDQKDEAEKAKRMKEWMKRIKTTAS
>Neo2B_Split_Neo215_H32’4
(MGSHHHHHHGSGSENLYFQGSG)TNSPPAEEKLEDYAFNFELILEEIARLFESGDQKDEAEKA
KRMKEWMKRIKTTASEDEQEEMANAIITILQSWIFS

>αHER2_Neo2/15
(MGSHHHHHHGSGSENLYFQGSGSG)DLGKKLLEAARAGQDDEVRILMANGADVNAKDEYGLTP
LYLATAHGHLEIVEVLLKNGADVNAVDAIGFTPLHLAAFIGHLEIAEVLLKHGADVNAQDKFGK
TAFDISIGNGNEDLAEILQKLN(**GSGGSGGGSGGSGSG)**PKKKIQLHAEHALYDALMILNIVKT
NSPPAEEKLEDYAFNFELILEEIARLFESGDQKDEAEKAKRMKEWMKRIKTTASEDEQEEMANA
IITILQSWIFS

>αHER2_Neo2A
(MGSHHHHHHGSGSENLYFQGSGSG)DLGKKLLEAARAGQDDEVRILMANGADVNAKDEYGLTP
LYLATAHGHLEIVEVLLKNGADVNAVDAIGFTPLHLAAFIGHLEIAEVLLKHGADVNAQDKFGK
TAFDISIGNGNEDLAEILQKLN(**GSGGSGGGSGGSGSG)**PKKKIQLHAEHALYDALMILNIVKT
NS

>αHER2_Neo2B
(MGSHHHHHHGSGSENLYFQGSGSG)DLGKKLLEAARAGQDDEVRILMANGADVNAKDEYGLTP
LYLATAHGHLEIVEVLLKNGADVNAVDAIGFTPLHLAAFIGHLEIAEVLLKHGADVNAQDKFGK
TAFDISIGNGNEDLAEILQKLN(**GSGGSGGGSGGSGSG)**TNSPPAEEKLEDYAFNFELILEEIA
RLFESGDQKDEAEKAKRMKEWMKRIKTTASEDEQEEMANAIITILQSWIFS

>αHER2_Neo2A-30linker
(MGSHHHHHHGSGSENLYFQGSGSG)DLGKKLLEAARAGQDDEVRILMANGADVNAKDEYGLTP
LYLATAHGHLEIVEVLLKNGADVNAVDAIGFTPLHLAAFIGHLEIAEVLLKHGADVNAQDKFGK
TAFDISIGNGNEDLAEILQKLN(**GGSGGSGGGSGGSGGGGSGGSGGGSGGGGS)**PKKKIQLHAE
HALYDALMILNIVKTNS

>αHER2_H132’
(MGSHHHHHHGSGSENLYFQGSGSG)DLGKKLLEAARAGQDDEVRILMANGADVNAKDEYGLTP
LYLATAHGHLEIVEVLLKNGADVNAVDAIGFTPLHLAAFIGHLEIAEVLLKHGADVNAQDKFGK
TAFDISIGNGNEDLAEILQKLN(**GSGGSGGGSGGSGSG)**PKKKIQLHAEHALYDALMILNIVKT
NSPPAEEKLEDYAFNFELILEEIARLFESGDQKDEAEKAKRMKEWMKRIKTTAS

>αHER2_H13
(MGSHHHHHHGSGSENLYFQGSGSG)DLGKKLLEAARAGQDDEVRILMANGADVNAKDEYGLTP
LYLATAHGHLEIVEVLLKNGADVNAVDAIGFTPLHLAAFIGHLEIAEVLLKHGADVNAQDKFGK
TAFDISIGNGNEDLAEILQKLN(**GSGGSGGGSGGSGSG)**PKKKIQLHAEHALYDALMILNIVKT
NSPPAEEKLEDYAFNFELILEEIARLFESG

>αHER2_H4
(MGSHHHHHHGSGSENLYFQGSGSG) DLGKKLLEAARAGQDDEVRILMANGADVNAKDEYGLTP
LYLATAHGHLEIVEVLLKNGADVNAVDAIGFTPLHLAAFIGHLEIAEVLLKHGADVNAQDKFGK
TAFDISIGNGNEDLAEILQKLN (**GSGGSGGGSGGSGSG)** TTASEDEQEEMANAIITILQSWIFS

>αHER2_H2'4
(MGSHHHHHHGSGSENLYFQGSGSG) DLGKKLLEAARAGQDDEVRILMANGADVNAKDEYGLTP
LYLATAHGHLEIVEVLLKNGADVNAVDAIGFTPLHLAAFIGHLEIAEVLLKHGADVNAQDKFGK
TAFDISIGNGNEDLAEILQKLN (**GSGGSGGGSGGSGSG)** DQKDEAEKAKRMKEWMKRIKTTASE
DEQEEMANAIITILQSWIFS

>αEGFR_Neo2/15
(MGSHHHHHHGSGSENLYFQGSGGG) PKKKIQLHAEHALYDALMILNIVKTNSPPAEEKLEDYA
FNFELILEEIARLFESGDQKDEAEKAKRMKEWMKRIKTTASEDEQEEMANAIITILQSWIFS (**G
SGGSGGGSGGSGSGGSGGG)** DLGKKLLEAARAGQDDEVRILMANGADVNADDTWGWTPLHLAAY
QGHLEIVEVLLKNGADVNAYDYIGWTPLHLAADGHLEIVEVLLKNGADVNASDYIGDTPLHLAA
HNGHLEIVEVLLKHGADVNAQDKFGKTAFDISIDNGNEDLAEILQKL

>αEGFR_Neo2A
(MGSHHHHHHGSGSENLYFQGSGGG) PKKKIQLHAEHALYDALMILNIVKTNS (**GSGGSGGGSG
GSGSGGSGGG)** DLGKKLLEAARAGQDDEVRILMANGADVNADDTWGWTPLHLAAYQGHLEIVEV
LLKNGADVNAYDYIGWTPLHLAADGHLEIVEVLLKNGADVNASDYIGDTPLHLAAHNGHLEIVE
VLLKHGADVNAQDKFGKTAFDISIDNGNEDLAEILQKLN

>αEGFR_Neo2B
(MGSHHHHHHGSGSENLYFQGSGGG) TNSPPAEEKLEDYAFNFELILEEIARLFESGDQKDEAE
KAKRMKEWMKRIKTTASEDEQEEMANAIITILQSWIFS (**GSGGSGGGSGGSGSGGSGGG)** DLGK
KLLEAARAGQDDEVRILMANGADVNADDTWGWTPLHLAAYQGHLEIVEVLLKNGADVNAYDYIG
WTPLHLAADGHLEIVEVLLKNGADVNASDYIGDTPLHLAAHNGHLEIVEVLLKHGADVNAQDKF
GKTAFDISIDNGNEDLAEILQKLN

>αEGFR_Neo2B_30linker
(MGSHHHHHHGSGSENLYFQGSGGG) TNSPPAEEKLEDYAFNFELILEEIARLFESGDQKDEAE
KAKRMKEWMKRIKTTASEDEQEEMANAIITILQSWIFS (**GGSGGSGGGSGGSGGGGSGGSGGGS
GGGGS)** DLGKKLLEAARAGQDDEVRILMANGADVNADDTWGWTPLHLAAYQGHLEIVEVLLKNG
ADVNAYDYIGWTPLHLAADGHLEIVEVLLKNGADVNASDYIGDTPLHLAAHNGHLEIVEVLLKH
GADVNAQDKFGKTAFDISIDNGNEDLAEILQKLN

>αEGFR_H13
(MGSHHHHHHGSGSENLYFQGSGGG) PKKKIQLHAEHALYDALMILNIVKTNSPPAEEKLEDYA
FNFELILEEIARLFESG (**GSGGSGGGSGGSGSGGSGGG)** DLGKKLLEAARAGQDDEVRILMANG
ADVNADDTWGWTPLHLAAYQGHLEIVEVLLKNGADVNAYDYIGWTPLHLAADGHLEIVEVLLKN
GADVNASDYIGDTPLHLAAHNGHLEIVEVLLKHGADVNAQDKFGKTAFDISIDNGNEDLAEILQ
KLN

>αEGFR_H132'
(MGSHHHHHHGSGSENLYFQGSGGG) PKKKIQLHAEHALYDALMILNIVKTNSPPAEEKLEDYA

FNFELILEEIARLFESGDQKDEAEKAKRMKEWMKRIKTTAS**(GSGGSGGGSGGSGSGGSGGG)**D
LGKKLLEAARAGQDDEVRILMANGADVNADDTWGWTPLHLAAYQGHLEIVEVLLKNGADVNAYD
YIGWTPLHLAADGHLEIVEVLLKNGADVNASDYIGDTPLHLAAHNGHLEIVEVLLKHGADVNAQ
DKFGKTAFDISIDNGNEDLAEILQKLN

>αEGFR_H2’4
(MGSHHHHHHGSGSENLYFQGSGGG)DQKDEAEKAKRMKEWMKRIKTTASEDEQEEMANAIITI
LQSWIFS**(GSGGSGGGSGGSGSGGSGGG)**DLGKKLLEAARAGQDDEVRILMANGADVNADDTWG
WTPLHLAAYQGHLEIVEVLLKNGADVNAYDYIGWTPLHLAADGHLEIVEVLLKNGADVNASDYI
GDTPLHLAAHNGHLEIVEVLLKHGADVNAQDKFGKTAFDISIDNGNEDLAEILQKLN

>αEGFR_H4
(MGSHHHHHHGSGSENLYFQGSGGG)TTASEDEQEEMANAIITILQSWIFS**(GSGGSGGGSGGS**
GSGGSGGG)DLGKKLLEAARAGQDDEVRILMANGADVNADDTWGWTPLHLAAYQGHLEIVEVLL
KNGADVNAYDYIGWTPLHLAADGHLEIVEVLLKNGADVNASDYIGDTPLHLAAHNGHLEIVEVL
LKHGADVNAQDKFGKTAFDISIDNGNEDLAEILQKLN

>αPD-L1_Neo2A
(MGSHHHHHHGSGSENLYFQGSGSG)PKKKIQLHAEHALYDALMILNIVKTNS**(GGGSGGSGGG**
SGGSGSG)QVQLVETGGGLVQPGGSLRLSCTASGFTFSMHAMTWYRQAPGKQRELVAVITSHGD
RANYTDSVRGRFTISRDNTKNMVYLQMNSLKPEDTAVYYCNVPRYDSWGQGTQVTVSSGG

>αPD-L1-Neo2B
(MGSHHHHHHGSGSENLYFQGSGSG)QVQLVETGGGLVQPGGSLRLSCTASGFTFSMHAMTWYR
QAPGKQRELVAVITSHGDRANYTDSVRGRFTISRDNTKNMVYLQMNSLKPEDTAVYYCNVPRYD
SWGQGTQVTVSSGG**(GGGSGGSGGGSGGSGSG)**TNSPPAEEKLEDYAFNFELILEEIARLFESG
DQKDEAEKAKRMKEWMKRIKTTASEDEQEEMANAIITILQSWIFS

>αPD-L1-Neo2/15
(MGSHHHHHHGSGSENLYFQGSGSG)QVQLVETGGGLVQPGGSLRLSCTASGFTFSMHAMTWYR
QAPGKQRELVAVITSHGDRANYTDSVRGRFTISRDNTKNMVYLQMNSLKPEDTAVYYCNVPRYD
SWGQGTQVTVSSGG**(GGGSGGSGGGSGGSGSG)**PKKKIQLHAEHALYDALMILNIVKTNSPPAE
EKLEDYAFNFELILEEIARLFESGDQKDEAEKAKRMKEWMKRIKTTASEDEQEEMANAIITILQ
SWIFS

>CtrlVHH_Neo2A
(MGSHHHHHHGSGSENLYFQGSGSG)PKKKIQLHAEHALYDALMILNIVKTNS**(GSGGSGGGSG**
GSGSG)QVQLVETGGGLVQPGESLRLSCVASGFTLDHSAVGWFRQVPGKEREKLLCINANGVSL
DYADSIKGRFTISRDNAKNTVYLQMNDLKPEDTATYSCAATREFCSAYVFLYEHWGQGTQVTVS
S

>CtrlVHH-Neo2B
(MGSHHHHHHGSGSENLYFQGSGSG)QVQLVETGGGLVQPGESLRLSCVASGFTLDHSAVGWFR
QVPGKEREKLLCINANGVSLDYADSIKGRFTISRDNAKNTVYLQMNDLKPEDTATYSCAATREF
CSAYVFLYEHWGQGTQVTVSS**(GSGGSGGGSGGSGSG)**TNSPPAEEKLEDYAFNFELILEEIAR
LFESGDQKDEAEKAKRMKEWMKRIKTTASEDEQEEMANAIITILQSWIFS

>CtrlVHH-Neo2/15
(MGSHHHHHHGSGSENLYFQGSGSG) QVQLVETGGGLVQPGESLRLSCVASGFTLDHSAVGWFR QVPGKEREKLLCINANGVSLDYADSIKGRFTISRDNAKNTVYLQMNDLKPEDTATYSCAATREF CSAYVFLYEHWGQGTQVTVSS (**GSGGSGGGSGGSGSG)** PKKKIQLHAEHALYDALMILNIVKTN SPPAEEKLEDYAFNFELILEEIARLFESGDQKDEAEKAKRMKEWMKRIKTTASEDEQEEMANAI ITILQSWIFS

>αEpCAM_Neo2A
(MGSHHHHHHGSGSENLYFQGSGGS) DLGKKLLEAARAGQDDEVRILVANGADVNAYFGTTPLH LAAAHGRLEIVEVLLKNGADVNAQDVWGITPLHLAAYNGHLEIVEVLLKYGADVNAHDTRGWTP LHLAAINGHLEIVEVLLKNVADVNAQDRSGKTPFDLAIDNGNEDIAEVLQKAAKLN (**GGSGGSG GGSGGSGSG)** PKKKIQLHAEHALYDALMILNIVKTNS

>αEpCAM_Neo2B
(MGSHHHHHHGSGSENLYFQGSGGS) DLGKKLLEAARAGQDDEVRILVANGADVNAYFGTTPLH LAAAHGRLEIVEVLLKNGADVNAQDVWGITPLHLAAYNGHLEIVEVLLKYGADVNAHDTRGWTP LHLAAINGHLEIVEVLLKNVADVNAQDRSGKTPFDLAIDNGNEDIAEVLQKAAKLN (**GGSGGSG GGSGGSGSG)** TNSPPAEEKLEDYAFNFELILEEIARLFESGDQKDEAEKAKRMKEWMKRIKTTA SEDEQEEMANAIITILQSWIFS
