# Supplementary material for: A split, conditionally active mimetic of IL-2 reduces the toxicity of systemic cytokine therapy
Source: Nat Biotechnol. 2022 Oct 31;41(4):532–40. doi: 10.1038/s41587-022-01510-z (PMC10110466; doi:10.1038/s41587-022-01510-z)
Supplement: Supplementary file 2 [file 41587_2022_1510_MOESM2_ESM.pdf]

Corresponding author(s): David Baker, Daniel-Adriano Silva, Michael Dougan, Jamie B. Spangler

Last updated by author(s): 08/21/2022

Reporting Summary

Nature Portfolio wishes to improve the reproducibility of the work that we publish. This form provides structure for consistency and transparency in reporting. For further information on Nature Portfolio policies, see our Editorial Policies and the Editorial Policy Checklist.

Statistics

For all statistical analyses, confirm that the following items are present in the figure legend, table legend, main text, or Methods section.

n/a	Confirmed	
☐	☒	The exact sample size (*n*) for each experimental group/condition, given as a discrete number and unit of measurement
☐	☒	A statement on whether measurements were taken from distinct samples or whether the same sample was measured repeatedly
☐	☒	The statistical test(s) used AND whether they are one- or two-sided<br>*Only common tests should be described solely by name; describe more complex techniques in the Methods section.*
☒	☐	A description of all covariates tested
☒	☐	A description of any assumptions or corrections, such as tests of normality and adjustment for multiple comparisons
☐	☒	A full description of the statistical parameters including central tendency (e.g. means) or other basic estimates (e.g. regression coefficient) AND variation (e.g. standard deviation) or associated estimates of uncertainty (e.g. confidence intervals)
☐	☒	For null hypothesis testing, the test statistic (e.g. *F*, *t*, *r*) with confidence intervals, effect sizes, degrees of freedom and *P* value noted<br>*Give P values as exact values whenever suitable.*
☒	☐	For Bayesian analysis, information on the choice of priors and Markov chain Monte Carlo settings
☒	☐	For hierarchical and complex designs, identification of the appropriate level for tests and full reporting of outcomes
☒	☐	Estimates of effect sizes (e.g. Cohen's *d*, Pearson's *r*), indicating how they were calculated

Our web collection on statistics for biologists contains articles on many of the points above.

Software and code

Policy information about availability of computer code

Data collection	Biolayer interferometry data were acquired on an OctetRED96 (ForteBio). Far-ultraviolet circular dichroism measurements were carried out with an AVIV spectrometer model 420. In vitro K562 targeting experiments data were acquired on a LSR II Flow cytometer (BD Biosciences). In vitro SKOV3, OVCAR8, JIMT1 and B16F10 targeting experiments data were acquired on a FACSCelesta cytometer (BD Biosciences). Data for In-vitro Stat5 phosphorylation experiments were acquired with a CytoFLEX Flow cytometer (Beckman-Coulter). Flow cytometry data for B16F10 melanoma studies were collected on a SP6800 Spectral Analyzer (Sony Biotechnology). Data for in vitro CAR-T cell in vitro Stat5 phosphorylation were acquired with LSRFortessa™ (BD Biosciences). Cell electrical impedance measurements for in vitro tumor (NCI-H1975 cells) killing experiments was acquired with an xCelligence Esight instrument (Agilent).
Data analysis	Biolayer interferometry data were analyzed using ForteBio Data Analysis Software version 9.0.0.10 and plotted using GraphPad Prism (version 8 and 9). Cell cytometry data were analyzed using FlowJo (v9 and v10 software. All protein structure images were generated using PyMOL 2.0.

For manuscripts utilizing custom algorithms or software that are central to the research but not yet described in published literature, software must be made available to editors and reviewers. We strongly encourage code deposition in a community repository (e.g. GitHub). See the Nature Portfolio guidelines for submitting code & software for further information.

Data

Policy information about availability of data

All manuscripts must include a data availability statement. This statement should provide the following information, where applicable:

- Accession codes, unique identifiers, or web links for publicly available datasets
- A description of any restrictions on data availability
- For clinical datasets or third party data, please ensure that the statement adheres to our policy

The original experimental data that supports the findings of this work are available from the corresponding authors upon request. Plasmids encoding the proteins described in this article are available from the corresponding authors upon request. The original Neo-2/15 crystallographic structure can be accessed at the Protein Data Bank database, accession number 6dg5.

Field-specific reporting

Please select the one below that is the best fit for your research. If you are not sure, read the appropriate sections before making your selection.

☒ Life sciences ☐ Behavioural & social sciences ☐ Ecological, evolutionary & environmental sciences

For a reference copy of the document with all sections, see nature.com/documents/nr-reporting-summary-flat.pdf

Life sciences study design

All studies must disclose on these points even when the disclosure is negative.

Sample size

In vitro Stat5 experiments: Sample size was based on historical data which revealed statistical significance in previous experiments (1-3).

In vitro B16 trans-presentation assay: Sample size was based on historical data for in vitro T cell-tumor cell co-culture assays which revealed significance in previous experiments (1).

In vitro splenocyte assay: Sample size was based on historical data which revealed statistical significance in previous experiments (1).

Safety study: Sample size was based on historical data for similar IL-2 and Neo-2/15 toxicity experiments (1) which revealed statistical significance in previous experiments.

In vivo CD8 expansion experiments: Sample size was based on historical data for Neo-2/15 in vivo expansion experiments (1) which revealed statistical significance in previous experiments.

Melanoma in vivo survival experiments: Sample size was based on historical data for similar efficacy experiments treating with Neo-2/15 (1) which revealed statistical significance in previous experiments.

Melanoma re-challenge experiment: Sample size was based on historical data which revealed statistical significance in previous experiments.

CAR-T cell experiments: Sample size was based on historical data which revealed statistical significance in prior accepted high impact manuscripts within the CD19 CAR field such as the following:
• Qin et al., CAR T cells targeting BAFF-R can overcome CD19 antigen loss in B cell malignancies, Science Translational Medicine, 2019. (Figure 2, 3, 4C, D, 5D, E)
• Liu et al., Inclusion of Strep-Tag II in design of antigen receptors for T cell immunotherapy, Nature Biotechnology, 2016 (Figure 1 D, 2D, 3E - G)
• Mansilla-Soto et al., HLA-independent T cell receptors for targeting tumors with low antigen density, Nature Medicine, 2022 (Figure 4 Panel A, B, D, G)

(1) Silva, D.-A. et al. De novo design of potent and selective mimics of IL-2 and IL-15. Nature 565, 186–191 (2019).
(2) Mitra, S. et al. Interleukin-2 activity can be fine tuned with engineered receptor signaling clamps. Immunity 42, 826–838 (2015).
(3) Ring, A. M. et al. Mechanistic and structural insight into the functional dichotomy between IL-2 and IL-15. Nat. Immunol. 13, 1187–1195 (2012).

Data exclusions

Biolayer Interferometry binding studies: No data were excluded from the analyses.

In vitro Stat5 experiments: No data were excluded from the analyses.

In vitro B16 trans-presentation assay: No data were excluded from the analysis.

In vitro splenocyte assay: No data were excluded from the analysis.

Safety study: No data were excluded from the analysis.

In vivo CD8 expansion experiments: No data were excluded from the analyses.

Melanoma in vivo survival experiments: No data were excluded from the analyses.

Melanoma re-challenge experiment: No data were excluded from the analyses.

CAR-T cell experiments: No data were excluded from the analyses.

Replication

Biolayer Interferometry binding studies: all binding experiments were performed more than 3 times with different batches of purified protein. All replication attempts resulted in similar results.

In vitro Stat5 experiments: All STAT5 phosphorylation studies were performed in triplicate and iterated at least 2 times to ensure reproducibility. All replication attempts resulted in similar results.

In vitro B16F10 trans-presentation assay: Experiment and conditions were performed in triplicate and repeated a minimum of two times to ensure reproducibility. All replication attempts resulted in similar results.

In vitro splenocyte proliferation assay: All experiments and conditions were performed in triplicate and repeated twice. All replication attempts resulted in similar results.

Safety study: The experiment was performed once with enough subjects per group. Findings were supported by additional experiments testing constructs at other doses.

In vivo CD8 expansion experiments: Experiment and conditions were repeated a minimum of two times to ensure reproducibility. All replication attempts resulted in similar results.

Melanoma in vivo survival experiments: Experiment and conditions were repeated a minimum of two times to ensure reproducibility.

Melanoma re-challenge experiment: Experiment was not repeated, but multiple subjects were included in each experimental group.

CAR-T cell experiments: experiment was not repeated, but multiple subjects were included in each experimental group.

Randomization

In vitro B16F10 trans-presentation assay: Randomization not relevant as established cell lines were used.

In vitro splenocyte proliferation assays: Splenocytes obtained from randomly selected mice purchased from the Jackson Laboratory.

Safety study: Mice were purchased from the Jackson Laboratory and randomly allocated into experimental groups prior to start of specific treatments.

In vivo CD8 expansion experiments: FoxP3 mice were purchased from the Jackson Laboratory and bred in-house. The mice were randomly allocated into experimental groups prior to start of specific treatments.

Melanoma in vivo survival experiments: Mice used in the in vivo experiments were purchased from the Jackson Laboratory. They were given tumors and then randomized after tumor injection into experimental groups prior to start of specific treatments.

Melanoma re-challenge experiment: Mice in the re-challenge cohort were pooled from survivors from different experiments that received TA99 + anti-PD-L1 splits treatment. Mice in the control cohort were randomly selected from large selection of mice purchased from the Jackson Laboratory. 5 melanoma cured mice and 5 melanoma naïve mice were pooled, quickly inoculated with tumors in a random order, and then randomly separated into two boxes. The melanoma cured mice were already earmarked, which allowed for tracking of the experimental groups overtime.

In vitro Stat5 experiments: Randomization not relevant because established cell lines were used.

CAR-T cell experiments: NSG mice used in the in vivo experiments were acquired from the Jackson laboratory. They were given tumors and then randomized after tumor injection into experimental groups prior to start of specific treatments.

Blinding

Investigators were not blinded to the treatment groups because the investigators administering daily dosing were the same ones analyzing the results.

Reporting for specific materials, systems and methods

We require information from authors about some types of materials, experimental systems and methods used in many studies. Here, indicate whether each material, system or method listed is relevant to your study. If you are not sure if a list item applies to your research, read the appropriate section before selecting a response.

Materials & experimental systems

n/a	Involved in the study
☐	☒ Antibodies
☐	☒ Eukaryotic cell lines
☒	☐ Palaeontology and archaeology
☐	☒ Animals and other organisms
☐	☒ Human research participants
☒	☐ Clinical data
☒	☐ Dual use research of concern

Methods

n/a	Involved in the study
☒	☐ ChIP-seq
☐	☒ Flow cytometry
☒	☐ MRI-based neuroimaging

Antibodies

Antibodies used

In vitro Stat5 experiments:
- Alexa Fluor® 647-conjugated mouse anti-STAT5 pY694 (BD Biosciences, 612599, Clone 47/Stat5(pY694)). 1:50 dilution.
- BV421-conjugated anti-CD132 antibody (BD Biosciences, 566222, Clone AG184). 1:50 dilution.
- P-stat5-APC (A17016B.Rec, Biolegend, Cat # 936902) used at 1:200 dilution.

In vivo CD8 expansion experiments (Biolegend Antibodies):
- APC anti-CD45 (clone 30-F11, catalog no. 103111). Used at 1:100 dilution.
- Brilliant Violet 421 anti-CD4 (GK1.5, 100443). Used at 1:100 dilution.
- PE anti-CD8a (53-6.7, 100707). Used at 1:100 dilution.

In vitro aCD8 split splenocyte assays (Biolegend Antibodies):
- APC anti-CD45 (clone 30-F11, catalog no. 103111). Used at 1:100 dilution.
- Zombie NIRTM Fixable Viability Kit (catalog no. 423105). Used at 1:100 dilution.
- Pacific Blue anti- B220 (RA3-6B2,103227). Used at 1:100 dilution.
- Brilliant Violet 421 anti-CD4 (GK1.5, 100443). Used at 1:100 dilution.

In vitro B16 Trans presentation assays (Biolegend Antibodies):
- Pacific Blue anti-CD8a (clone 53-6.7, catalog no. 100728). Used at 1:100 dilution.
- Alexa Fluor ® anti-CD25 (clone PC61, 102017). Used at 1:100 dilution.
- PE anti-CD69 (clone H1.2F3, 104508). Used at 1:100 dilution.

In vivo aPDL1 melanoma experiments (Biolegend Antibodies):
- Alexa Fluor ® 488 anti-CD25 (clone PC61, catalog no. 102018). Used at 1:100 dilution.
- PE/Cyanine 7 anti-CD69 (H1.2F3, 104511). Used at 1:100 dilution.
- Pacific Blue anti-CD8a (53-6.7, 100728). Used at 1:100 dilution.
- Brilliant Violet 510 anti-CD4 (RM4-5, 100553). Used at 1:100 dilution.
- Brilliant Violet 711 anti-CD45 (30-F11, 103147). Used at 1:100 dilution.

In vivo safety Study
- Alexa Fluor ® 488 anti-CD25 (clone PC61, catalog no. 102018). Used at 1:100 dilution.
- PE anti-CD69 (H1.2F3, 104508). Used at 1:100 dilution.
- Pacific Blue anti-CD4 (GK1.5, 100428). Used at 1:100 dilution.
- Brilliant Violet 711 anti-CD45 (30-F11, 103147). Used at 1:100 dilution.
- Brilliant Violet 785 anti-CD8a (53-6.7, 100749). Used at 1:100 dilution.

Surface receptor quantification:
- human EGFR (AY13, Biolegend, catalogue no. 352903) 1:200 dilution.
- human EpCAM (9C4, Biolegend, catalogue # 324205), 1:200 dilution.
- human HER2 (24D2, Biolegend, catalogue # 324405), 1:200 dilution.
- murine PD-L1 (MIH7, Biolegend, catalogue # 155404), 1:200 dilution.

Validation

All listed antibodies were validated by the respective manufacturer as can be observed on the manufacturer's website.

In vitro Stat5 experiments: Alexa Fluor® 647-conjugated mouse anti-STAT5 pY694 (BD Biosciences, 612599, Clone 47/Stat5(pY694)). Antibody was used at a saturating concentration of 1:50 based on titrations centered around manufacturer recommendations. The antibody was validated using appropriate positive (cytokine-stimulated cells) and negative (unstimulated cells) controls, consistent with manufacturer validation on the product website: https://www.bdbiosciences.com/en-us/products/reagents/flow-cytometry-reagents/research-reagents/single-color-antibodies-ruo/alexa-fluor-647-mouse-anti-stat5-py694.612599.

- BV421-conjugated anti-CD132 antibody (BD Biosciences, 566222, Clone AG184)
Antibody was used at a saturating concentration of 1:50 based on titrations centered around manufacturer recommendations. The antibody was validated using appropriate positive ([illegible]c-expressing) and negative (common gamma-nonexpressing) controls, consistent with manufacturer validation on the website: https://www.bdbiosciences.com/en-us/products/reagents/flow-cytometry-reagents/research-reagents/single-color-antibodies-ruo/bv421-mouse-anti-human-cd132.566222
P-stat5-APC (A17016B.Rec, biolegend) used at 1:200.
Antibody was used at a saturating concentration of 1:200 based on titrations centered around manufacturer recommendations. The antibody was validated using human CD8 T cells that were serum starved for 3 hours and post stimulation with 100IUs of IL2 for

12mins or without IL2 treatment (negative control). The antibody staining was consistent with manufacturer's product information stated on the website: https://www.biolegend.com/en-us/products/apc-anti-stat5-phospho-tyr694-antibody-20812

In vivo CD8 expansion experiments (Biolegend Antibodies):
- APC anti-CD45 (clone 30-F11, catalog no. 103111)
Antibody was used at a 1:100 dilution. The antibody was validated using appropriate positive (CD45+ cells) and negative (CD45- cells) controls as well as with a concentration-matched isotype control, consistent with manufacturer validation on the product website: https://www.biolegend.com/en-us/punchout/search-results/apc-anti-mouse-cd45-antibody-97?GroupID=GROUP20

- Brilliant Violet 421 anti-CD4 (GK1.5, 100443)
Antibody was used at a 1:100 dilution. The antibody was validated using appropriate positive (CD4+ cells) and negative (CD4- cells) controls as well as with a concentration-matched isotype control, consistent with manufacturer validation on the product website: https://www.biolegend.com/en-us/punchout/punchout-products/product-detail/brilliant-violet-421-anti-mouse-cd4-antibody-7142

- PE anti-CD8a (53-6.7, 100707)
Antibody was used at a 1:100 dilution. The antibody was validated using appropriate positive (CD8+ cells) and negative (CD8- cells) controls as well as with a concentration-matched isotype control, consistent with manufacturer validation on the product website: https://www.biolegend.com/en-us/punchout/punchout-products/product-detail/pe-anti-mouse-cd8a-antibody-155

In vitro aCD8 split splenocyte assays (Biolegend Antibodies):
- APC anti-CD45 (clone 30-F11, catalog no. 103111)
Antibody was used at a 1:100 dilution. The antibody was validated using appropriate positive (CD45+ cells) and negative (CD45- cells) controls as well as with a concentration-matched isotype control, consistent with manufacturer validation on the product website: https://www.biolegend.com/en-us/punchout/search-results/apc-anti-mouse-cd45-antibody-97?GroupID=GROUP20

- Zombie NIRTM Fixable Viability Kit (catalog no. 423105)
Antibody was used at a 1:100 dilution. The antibody was validated using appropriate positive (live and proliferating cells) and negative (fixed cells) controls as well as with a concentration-matched isotype control, consistent with manufacturer validation on the product website:
https://www.biolegend.com/en-us/punchout/punchout-products/product-detail/zombie-nir-fixable-viability-kit-8657

- Pacific Blue anti- B220 (RA3-6B2,103227)
Antibody was used at a 1:100 dilution. The antibody was validated using appropriate positive (B220+ cells) and negative (B220- cells) controls as well as with a concentration-matched isotype control, consistent with manufacturer validation on the product website: https://www.biolegend.com/en-us/punchout/punchout-products/product-detail/pacific-blue-anti-mouse-human-cd45r-b220-antibody-2857

- Brilliant Violet 421 anti-CD4 (GK1.5, 100443)
Antibody was used at a 1:100 dilution. The antibody was validated using appropriate positive (CD4+ cells) and negative (CD4- cells) controls as well as with a concentration-matched isotype control, consistent with manufacturer validation on the product website: https://www.biolegend.com/en-us/punchout/punchout-products/product-detail/brilliant-violet-421-anti-mouse-cd4-antibody-7142

In vitro B16 Trans presentation assays (Biolegend Antibodies):
- Pacific Blue anti-CD8a (clone 53-6.7, catalog no. 100728)
Antibody was used at a 1:100 dilution. The antibody was validated using appropriate positive (CD8+ cells) and negative (CD8- cells) controls as well as with a concentration-matched isotype control, consistent with manufacturer validation on the product website: https://www.biolegend.com/en-us/punchout/punchout-products/product-detail/pacific-blue-anti-mouse-cd8a-antibody-2856

- Alexa Fluor ® anti-CD25 (clone PC61, 102017)
Antibody was used at a 1:100 dilution. The antibody was validated using appropriate positive (cytokine-stimulated cells) and negative (unstimulated cells) controls as well as with a concentration-matched isotype control, consistent with manufacturer validation on the product website:
https://www.biolegend.com/en-us/punchout/punchout-products/product-detail/alexa-fluor-488-anti-mouse-cd25-antibody-2706

- PE anti-CD69 (clone H1.2F3, 104508)
Antibody was used at a 1:100 dilution. The antibody was validated using appropriate positive (cytokine-stimulated cells) and negative (unstimulated cells) controls as well as with a concentration-matched isotype control, consistent with manufacturer validation on the product website:
https://www.biolegend.com/en-us/punchout/punchout-products/product-detail/pe-anti-mouse-cd69-antibody-265

In vivo aPDL1 melanoma experiments (Biolegend Antibodies):
- Alexa Fluor ® 488 anti-CD25 (clone PC61, catalog no. 102018)
Antibody was used at a 1:100 dilution. The antibody was validated using appropriate positive (cytokine-stimulated cells) and negative (unstimulated cells) controls as well as with a concentration-matched isotype control, consistent with manufacturer validation on the product website:
https://www.biolegend.com/en-us/punchout/punchout-products/product-detail/alexa-fluor-488-anti-mouse-cd25-antibody-2706

- PE/Cyanine 7 anti-CD69 (H1.2F3, 104511)

Antibody was used at a 1:100 dilution. The antibody was validated using appropriate positive (cytokine-stimulated cells) and negative (unstimulated cells) controls as well as with a concentration-matched isotype control, consistent with manufacturer validation on the product website:
https://www.biolegend.com/en-us/punchout/punchout-products/product-detail/pe-anti-mouse-cd69-antibody-265

- Pacific Blue anti-CD8a (53-6.7, 100728)
Antibody was used at a 1:100 dilution. The antibody was validated using appropriate positive (CD8+ cells) and negative (CD8- cells) controls as well as with a concentration-matched isotype control, consistent with manufacturer validation on the product website:
https://www.biolegend.com/en-us/punchout/punchout-products/product-detail/pacific-blue-anti-mouse-cd8a-antibody-2856

- Brilliant Violet 510 anti-CD4 (RM4-5, 100553)
Antibody was used at a 1:100 dilution. The antibody was validated using appropriate positive (CD4+ cells) and negative (CD4- cells) controls as well as with a concentration-matched isotype control, consistent with manufacturer validation on the product website:
https://www.biolegend.com/en-us/punchout/punchout-products/product-detail/brilliant-violet-510-anti-mouse-cd4-antibody-10707

- Brilliant Violet 711 anti-CD45 (30-F11, 103147)
Antibody was used at a 1:100 dilution. The antibody was validated using appropriate positive (CD45+ cells) and negative (CD45- cells) controls as well as with a concentration-matched isotype control, consistent with manufacturer validation on the product website:
https://www.biolegend.com/en-us/punchout/punchout-products/product-detail/brilliant-violet-711-anti-mouse-cd45-antibody-10439

In vivo Toxicity Study
- Alexa Fluor ® 488 anti-CD25 (clone PC61, catalog no. 102018)
Antibody was used at a 1:100 dilution. The antibody was validated using appropriate positive (cytokine-stimulated cells) and negative (unstimulated cells) controls as well as with a concentration-matched isotype control, consistent with manufacturer validation on the product website:
https://www.biolegend.com/en-us/punchout/punchout-products/product-detail/alexa-fluor-488-anti-mouse-cd25-antibody-2706

- PE anti-CD69 (H1.2F3, 104508)
Antibody was used at a 1:100 dilution. The antibody was validated using appropriate positive (cytokine-stimulated cells) and negative (unstimulated cells) controls as well as with a concentration-matched isotype control, consistent with manufacturer validation on the product website:
https://www.biolegend.com/en-us/punchout/punchout-products/product-detail/pe-anti-mouse-cd69-antibody-265

- Pacific Blue anti-CD4 (GK1.5, 100428)
Antibody was used at a 1:100 dilution. The antibody was validated using appropriate positive (CD4+ cells) and negative (CD4- cells) controls as well as with a concentration-matched isotype control, consistent with manufacturer validation on the product website:
https://www.biolegend.com/en-us/punchout/punchout-products/product-detail/pacific-blue-anti-mouse-cd4-antibody-3316

- Brilliant Violet 711 anti-CD45 (30-F11, 103147)
Antibody was used at a 1:100 dilution. The antibody was validated using appropriate positive (CD45+ cells) and negative (CD45- cells) controls as well as with a concentration-matched isotype control, consistent with manufacturer validation on the product website:
https://www.biolegend.com/en-us/punchout/punchout-products/product-detail/brilliant-violet-711-anti-mouse-cd45-antibody-10439

- Brilliant Violet 785 anti-CD8a (53-6.7, 100749)
Antibody was used at a 1:100 dilution. The antibody was validated using appropriate positive (CD8+ cells) and negative (CD8- cells) controls as well as with a concentration-matched isotype control, consistent with manufacturer validation on the product website:
https://www.biolegend.com/en-us/punchout/punchout-products/product-detail/brilliant-violet-785-anti-mouse-cd8a-antibody-7957

PE human EGFR (AY13, Biolegend, cat # 352903)
Antibody was used at a saturating concentration of 1:XXX based on titrations centered around manufacturer recommendations. The antibody was validated by comparing staining on human T cells that were transduced to overexpress EGFR and untransduced human T cells (negative control).

PE human Her2 (24D2, Biolegend, cat # 324405)
Antibody was used at a saturating concentration of 1:200 based on titrations centered around manufacturer recommendations. The antibody was validated by comparing staining on human T cells that were transduced to overexpress Her2 and untransduced human T cells (negative control).

PE Human Epcam (9C4, Biolegend, catalogue # 324205)
Antibody was used at a saturating concentration of 1:200 based on titrations centered around manufacturer recommendations. The antibody was validated by comparing staining on NCI-1975 cells, which are published to express EPCAM, against negative isotype controls and human T cells.

PE Murine PD-L1 (MIH7, Biolegend, cat # 155404)
Antibody was used at a saturating concentration of 1:200 based on titrations centered around manufacturer recommendations. The antibody was validated by comparing staining on B16F10 murine cells that were transduced to overexpress murine PD-L1 and untransduced B16F10 cells.

Eukaryotic cell lines

Policy information about cell lines

Cell line source(s)	In vitro Stat5 experiments: YT-1 cells were provided by Yodoi (Yodoi, J. et al. TCGF (IL 2)-receptor inducing factor(s). I. Regulation of IL 2 receptor on a natural killer-like cell line (YT cells). J. Immunol. 134, 1623–1630 (1985)). K562 cells were purchased from ATCC. K562 cell lines engineered to express EGFR-iRFP, Her2-eGFP, or both were generated as described previously (Lajoie, M. J. et al. Designed protein logic to target cells with precise combinations of surface antigens. Science 369, 1637–1643 (2020)).<br><br>In vitro & in vivo B16 experiments: B16 F10 cells were purchased from ATCC. These cells were used to generate the PD-L1 overexpressing B16 cells and stored in house as described in Xie, Y. J. et al. Nanobody-based CAR T cells that target the tumor microenvironment inhibit the growth of solid tumors in immunocompetent mice. Proc. Natl. Acad. Sci. U. S. A. 116, 7624–7631 (2019).<br><br>CAR T cell experiments: Primary human CD8 T cells were obtained from healthy donors with written informed consent for research protocols approved by the Institutional Review Board of the Fred Hutchinson Cancer Research Center. Raji cells were purchased from ATCC and transduced with a lentiviral vector encoding the FLuc-eGFP fusion gene.<br><br>NCI-H1975, SKOV3, OVCAR8 and JIMT-1 cells were purchased from ATCC.<br><br>293-6E (human embryonic kidney) cell lines were provided by Proteos, Inc. and cultured in F17 supplemented with 0.1% Pluronic F-68, 4 mM GlutaMAX, 25 µg/mL G418.
Authentication	ATCC provided authentication for cell lines. Expression of transduced receptors fused to fluorescent proteins was monitored by flow cytometry. PD-L1 overexpression was validated by flow cytometry. Raji ff-luc cells were validated by measuring GFP expression by flow cytometry. H1975 cells were purchased from ATCC, evaluated for the right morphology by microscopy and grown in RPMI+5% FBS+ 10U/ml of pen/strep. Cells were trypsinized and split every three days to avoid confluency. The YT-1 cells were phenotyped by quantifying IL-2Ralpha, IL-2Rbeta, and common gamma expression compared to previous studies (1,2). Primary CAR-T cells or untransduced were validated by flow cytometry for truncated Her2 expression (transduction marker) or lack of expression prior to experiments.<br><br>B16F10 cell lines were obtained from American Type Culture Collection (ATCC). These cells were used at limited passage and were authenticated by morphology in tissue culture and by growth kinetics and appearance after inoculation into mice.<br><br>Mouse Trp-1 specific CD8 T cells are validated through flow cytometry staining of the peripheral blood with anti-CD8 and Trp-1 tetramers.<br><br>(1) Mitra, S. et al. Interleukin-2 activity can be fine tuned with engineered receptor signaling clamps. Immunity 42, 826–838 (2015).<br>(2) Ring, A. M. et al. Mechanistic and structural insight into the functional dichotomy between IL-2 and IL-15. Nat. Immunol. 13, 1187–1195 (2012).
Mycoplasma contamination	All cells tested negative for mycoplasma contamination via PCR assay.
Commonly misidentified lines<br>(See ICLAC register)	None.

Animals and other organisms

Policy information about studies involving animals; ARRIVE guidelines recommended for reporting animal research

Laboratory animals	Safety study: Female wild-type C57BL/6J mice aged 6-8 weeks were purchased from The Jackson Laboratory and used in the experiments. Mice were housed in rooms with temperatures between 68 to 75 degrees F. Humidity levels are maintained between 30 and 70 percent. Animal rooms are on 12:12 light:dark cycles.<br><br>In vivo CD8 expansion experiments: FoxP3-GFP mice were purchased from The Jackson Laboratory and used to breed mice in-house. Female mice aged 6-8 weeks were used in the experiments. Mice were housed in rooms with temperatures between 68 to 75 degrees F. Humidity levels are maintained between 30 and 70 percent. Animal rooms are on 12:12 light:dark cycles.<br><br>Melanoma in vivo survival experiments: Female wild-type C57BL/6J mice aged 6-8 weeks were purchased from The Jackson Laboratory and used in the experiments. Mice were housed in rooms with temperatures between 68 to 75 degrees F. Humidity levels are maintained between 30 and 70 percent. Animal rooms are on 12:12 light:dark cycles.<br><br>Melanoma re-challenge experiment: Female wild-type C57BL/6J mice were purchased from The Jackson Laboratory and used in the experiments. Mice were 12-20 weeks of age. Mice were housed in rooms with temperatures between 68 to 75 degrees F. Humidity levels are maintained between 30 and 70 percent. Animal rooms are on 12:12 light:dark cycles.<br><br>Car-T cell in vivo experiments: Six-to-eight-week-old female NSG mice were obtained from the Jackson Laboratory. Five mice were housed per cage. Temperature of the housing room was between 68 to 75 F degrees and humidity was between 40 to 60%. Mice were provided with cellulose pellets bedding (Performance BioFresh™), irradiated diamond twists as enrichment & nesting material

(Envigo), and irradiated lab diet #5058. Water bottles were filled in house with acidified water. Animal rooms are on 12:12 light:dark cycles.

Wild animals	None.
Field-collected samples	None.
Ethics oversight	All mouse procedures were ethically approved by the Institutional Animal Care and Use Committee (IACUC) at the Dana Farber Cancer Institute and the Fred Hutchinson Cancer Institute and The FHCRC Institutional Animal Care and Use Committee approved all mouse experiments conducted at the Fred Hutchinson Cancer Research Center

Note that full information on the approval of the study protocol must also be provided in the manuscript.

Human research participants

Policy information about studies involving human research participants

Population characteristics

Primary human CD8 T cells were obtained from healthy donors for the CAR T cell experiments.

Male or female ≥ 18 years of age, whom did not:

- 1) Weigh less than 110 pounds
- 2) Have donated blood or white blood cells in the past 2 months
- 3) Are anemic, pregnant or nursing
- 4) Have hepatitis or HIV
- 5) Are taking blood thinning or immunosuppressive medication
- 6) Have a cold, flu, or other infection
- 7) Have low blood pressure
- 8) Are participating in other studies such as vaccine trials

Recruitment

Potential donors are recruited by flyers or donor referrals. Study volunteers will go through a screening process to determine eligibility. Eligible donors will be assigned a unique de-identified code when added to the donor database upon study enrollment. We do not anticipate potential recruitment biases to impact results as long as the participant met the criteria outlined in the population characteristics.

Ethics oversight

The Fred Hutchinson Cancer Center Institutional Review Board

Note that full information on the approval of the study protocol must also be provided in the manuscript.

Flow Cytometry

Plots

Confirm that:

☒ The axis labels state the marker and fluorochrome used (e.g. CD4-FITC).

☒ The axis scales are clearly visible. Include numbers along axes only for bottom left plot of group (a 'group' is an analysis of identical markers).

☒ All plots are contour plots with outliers or pseudocolor plots.

☒ A numerical value for number of cells or percentage (with statistics) is provided.

Methodology

Sample preparation

In vitro cell-targeting experiments: The four K562 cell lines (Engineered K562 tumor cell lines transduced for expression of Her2+/eGFP+, EGFR+/iRFP+ or Her2+/eGFP+ and EGFR+/iRFP+ surface markers) were mixed in equivalent ratios. The cell mixtures were washed with flow buffer (20 mM Tris pH 8.0, 150 mM NaCl, 1 mM MgCl2, 1 mM CaCl2 and 1% BSA) and aliquoted into V-bottom plates with 200,000 cells/well. Serially diluted Split Neo-2/15 fusion proteins were made from concentrated stocks, and then added to the cells in a 50 µl volume and incubated for 30 minutes. Subsequently, the cells were washed with 150 µl of flow buffer and incubated with a mixture of biotinylated human IL-2Rγ (Acro Biosystems Cat no. ILG-H85E8), human IL-2Rβ (Acro Biosystems Cat no. CD2-H5221) and a fluorescent streptavidin-phycoerythrin conjugate (SA-PE, Invitrogen) for 15 minutes. Data were acquired on a LSRII (BD Biosciences) or a FACSCelesta (BD Biosciences).

In-vitro Stat5 experiments: Approximately 2x10^5 YT-1 cells were plated in each well of a 96-well plate and re-suspended in RPMI complete medium containing serial dilutions of targeted or untargeted intact or split Neo-2/15 proteins. Cells were stimulated for 20 min at 37ºC and immediately fixed by addition of formaldehyde to 1.5% and 10 min incubation at room temperature. After fixation, cells were permeabilized by resuspension in ice-cold 100% methanol for 30 min on ice. Fixed and permeabilized cells were washed twice with PBSA buffer (phosphate-buffered saline [PBS] pH 7.2 containing 0.1% bovine serum albumin) and then incubated with Alexa Fluor® 647-conjugated anti-STAT5 pY694 antibody (BD Biosciences) diluted 1:50 in PBSA buffer for 2 hr at room temperature. Cells were washed twice in PBSA buffer and Alexa Fluor® 647 was measured on a CytoFLEX flow cytometer (Beckman-Coulter). For co-culture assays, Approximately 2x10^5 YT-1 cells and the indicated ratio (K562:YT-1=20:1, 6:1, or 2:1) of K562 cells (Her2+/EGFR+ or Her2-/EGFR-) were used in each well for trans-activation studies. K562 cells were plated in each well of a 96-well plate and re-suspended in RPMI complete medium

containing serial dilutions of split Neo-2/15 proteins. Cells were incubated for 30 min at 37ºC and then washed once with PBSA buffer. YT-1 cells were added to each well and co-cultured with K562 cells for 30 min at 37ºC. Cells were stained with BV421-conjugated anti-CD132 antibody (BD Biosciences) for 30 min at 4ºC and washed once with PBSA buffer. After the wash, immediately fixed by addition of formaldehyde to 1.5% and 10 min incubation at room temperature. After fixation, cells were permeabilized by resuspension in ice-cold 100% methanol for 30 min on ice. Fixed and permeabilized cells were washed twice with PBSA buffer (phosphate-buffered saline [PBS] pH 7.2 containing 0.1% bovine serum albumin) and then incubated with Alexa Fluor® 647-conjugated anti-STAT5 pY694 antibody (BD Biosciences) diluted 1:50 in PBSA buffer for 2 hr at room temperature. Alexa Fluor® 647 fluorescence on YT-1 (gated on the anti-CD132 antibody) cells was measured on a CytoFLEX flow cytometer (Beckman-Coulter).
In vitro B16 trans-presentation assay: Cells were gently pipetted off, washed once with PBS, the cell pellet was resuspended and stained in PBS with 2% inactivated fetal calf serum for 20 min, washed once more with PBS, and analyzed.

In vitro splenocyte assay: Cells were gently pipetted off, washed once with PBS, the cell pellet was resuspended and stained in 2% inactivated fetal calf serum for 20 min, washed once more with PBS, and analyzed.

Safety study: Cell suspensions from spleens were washed once with PBS, the cell pellet was resuspended and stained in 2% inactivated fetal calf serum for 20 min, washed once more with PBS, and analyzed.

In vivo CD8 expansion experiments: Cell suspensions from lymph nodes and ACK lysed spleens were washed once with PBS, the cell pellet was resuspended and stained in 2% inactivated fetal calf serum for 20 min, washed once more with PBS, and analyzed.

Melanoma in vivo survival experiments: Cell suspensions from spleens, lymph nodes were washed once with PBS, the cell pellet was resuspended and stained in 2% inactivated fetal calf serum for 20 min, washed once more with PBS, and analyzed. Peripheral blood was collected in ACK lysis buffer, quenched with PBS after 1 min, washed, resuspended in ACK lysis buffer again for 1 min, washed once, and then resuspended and stained in 2% inactivated fetal calf serum for 20 min, washed once more with PBS, and analyzed.

CAR-T cell in vitro pSTAT5 experiment: CAR-T cells or T cells were starved with media without IL-2 for 4 hours and stimulated with Neo2/15 variants for 12min. Cells were then fixed with BD Fix buffer I at 37degrees for 10min then permeabilized with BD PERM buffer III on ice for 30min. Cells were washed twice and stained with pSTAT-PE antibody at 1:250 dilution for 30min at 4degrees. Cells were washed twice and resuspended in 50ul of wash buffer for flow cytometry.

Instrument

In vitro K562 targeting experiments data were acquired on a LSR II Flow cytometer (BD Biosciences). In vitro SKOV3, OVCAR8, JIMT1 and B16F10 targeting experiments data were acquired on a FACSCelesta cytometer (BD Biosciences). Data for In-vitro Stat5 phosphorylation experiments were acquired with a CytoFLEX Flow cytometer (Beckman-Coulter). Flow cytometry data for B16 melanoma studies were collected on a SP6800 Spectral Analyzer (Sony Biotechnology). Data for in vitro CAR-T cell in vitro Stat5 phosphorylation were acquired with LSRFortessa™ (BD Biosciences).

Software

In-vitro Stat5 experiments: BD FACSDiva or CytExpert Software were used for sample collection, FlowJo v10 was used to analyze data and Graphpad Prism 8 was used to plot the results.

Cell population abundance

CAR-T cell in vitro pSTAT5 experiment: CAR-T cells were sorted two days prior to assay based on truncated Her2 expression. Her2+ cells were about 50% of the entire live population.

Gating strategy

In vitro cell-targeting experiments: Cell populations were identified based on forward/side scatter profiles and singlets were discriminated using forward scatter area versus height plots. The four engineered K562 tumor cell lines transduced for expression of EGFR-iRFP, Her2-eGFP, both, or neither were identified by gating the cells for expression of eGFP and iRFP. Gating examples are shown in the supplementary information figures.

In-vitro Stat5 experiments: Cell populations were identified based on forward/side scatter profiles and singlets were discriminated using forward scatter area versus height plots. in co-culture assays, YT-1 cells were identified as iRFP-/eGFP-/CD132+. Gating examples are shown in the supplementary information figures.

In vitro B16 trans-presentation assay: T cells were identified based on forward/side scatter profiles and singlets were discriminated using forward scatter area versus height plots. After further confirmation via CD8+ gating, their CD25 and CD69 expression were quantified.

In vitro splenocyte assay: Live cells were identified based on forward/side scatter profiles and lack of Zombie NIR dead dye staining. B220+ cells were gated out, and on the remaining B220- cells, TCRbeta and CD4 were plotted to identify CD4 (+/+) and CD8 (+/-) populations, which were then quantified.

In vivo aCD8 and aPDL1 split mouse experiments: Lymphocytes were identified based on forward/side scatter profiles and singlets were discriminated using forward scatter area versus height plots. After CD45+ gating, the fraction of CD4 and CD8 cells were quantified. If relevant, CD25 and CD69 expression of the CD4 and CD8 populations were quantified.

CAR-T cell in vitro pSTAT5 experiment: Cell populations were identified based on forward/side scatter profiles and singlets were discriminated using forward scatter area versus height plots. T cells were gated based on standard lymphocyte gating. pSTAT5 positive cells were gated based on unstimulated control.

☒ Tick this box to confirm that a figure exemplifying the gating strategy is provided in the Supplementary Information.
